# Gut microbiome of helminth-infected indigenous Malaysians is context dependent

**DOI:** 10.1186/s40168-022-01385-x

**Published:** 2022-12-07

**Authors:** Mian Zi Tee, Yi Xian Er, Alice V. Easton, Nan Jiun Yap, Ii Li Lee, Joseph Devlin, Ze Chen, Kee Seong Ng, Poorani Subramanian, Angelina Angelova, Oyebola Oyesola, Shushan Sargsian, Romano Ngui, Daniel P. Beiting, Christopher Chiong Meng Boey, Kek Heng Chua, Ken Cadwell, Yvonne Ai Lian Lim, P’ng Loke, Soo Ching Lee

**Affiliations:** 1 Department of Biomedical Science, Faculty of Medicine, Universiti Malaya, Kuala Lumpur, Malaysia; 2 Department of Parasitology, Faculty of Medicine, Universiti Malaya, Kuala Lumpur, Malaysia; 3 Department of Microbiology, New York University Grossman School of Medicine, New York, NY USA; 4 Kulliyyah of Medicine and Health Sciences, University Islam Antarabangsa Sultan Abdul Halim Mu’adzam Shah, 09300 Kuala Ketil, Kedah Malaysia; 5 Department of Gastroenterology, Faculty of Medicine, Universiti Malaya, Kuala Lumpur, Malaysia; 6 Bioinformatics and Computational Biosciences Branch, Office of Cyber Infrastructure and Computational Biology, National Institute of Allergy and Infectious Diseases, National Institutes of Health, Bethesda, MD USA; 7 Type 2 Immunity Section, Laboratory of Parasitic Diseases, National Institute of Allergy and Infectious Diseases, National Institute of Health, Bethesda, MD USA; 8 Kimmel Center for Biology and Medicine at the Skirball Institute, New York University Grossman School of Medicine, New York, NY USA; 9 Department of Pathobiology, School of Veterinary Medicine, University of Pennsylvania, Philadelphia, PA USA; 10 Department of Paediatrics, Faculty of Medicine, Universiti Malaya, Kuala Lumpur, Malaysia; 11 Division of Gastroenterology, Department of Medicine, New York University Langone Health, New York, NY USA

**Keywords:** Helminth, Microbiome, Metagenomic sequencing, Indigenous population, Albendazole

## Abstract

**Background:**

While microbiomes in industrialized societies are well characterized, indigenous populations with traditional lifestyles have microbiomes that are more akin to those of ancient humans. However, metagenomic data in these populations remains scarce, and the association with soil-transmitted helminth infection status is unclear. Here, we sequenced 650 metagenomes of indigenous Malaysians from five villages with different prevalence of helminth infections.

**Results:**

Individuals from villages with higher prevalences of helminth infections have more unmapped reads and greater microbial diversity. Microbial community diversity and composition were most strongly associated with different villages and the effects of helminth infection status on the microbiome varies by village. Longitudinal changes in the microbiome in response to albendazole anthelmintic treatment were observed in both helminth infected and uninfected individuals. Inference of bacterial population replication rates from origin of replication analysis identified specific replicating taxa associated with helminth infection.

**Conclusions:**

Our results indicate that helminth effects on the microbiota were highly dependent on context, and effects of albendazole on the microbiota can be confounding for the interpretation of deworming studies. Furthermore, a substantial quantity of the microbiome remains unannotated, and this large dataset from an indigenous population associated with helminth infections is a valuable resource for future studies.

Video Abstract

**Supplementary Information:**

The online version contains supplementary material available at 10.1186/s40168-022-01385-x.

## Introduction

Industrialization is associated with reduced diversity of the microbiome in the human population [1], which could influence a range of physiological processes including nutrition, metabolism, immunity, neurochemistry, and drug metabolism [2]. Traditional indigenous populations have substantially greater microbial diversity than individuals living in industrialized societies. Nonetheless, our current knowledge of the human gut microbiome [3] is overrepresented by data available from industrialized countries and does not fully address the undersampling of indigenous populations.

Throughout evolution, helminths have coexisted with the gut microbiota in their mutual host [4], and the reduced prevalence of helminth infections from industrialized societies could contribute to the “hygiene hypothesis” [5]. While the effects of helminth colonization on the human gut microbiota have been studied, the results reported have been inconsistent. Some studies found that helminth colonization changes gut microbial diversity and composition and/or a shift in abundance of certain bacterial taxa [6–12], while others showed no apparent changes in gut microbial profiles [13, 14]. These divergent conclusions could be attributed to different confounders from different geographical locations (e.g., Malaysia [6, 12], Indonesia [9], Liberia [9], Tanzania [11], Western Kenya [10], and Ecuador [13]); different prevalence of helminth species (e.g., *Trichuris* sp. [13], hookworm [14], *Ascaris* sp. [10], *Strongyloides* sp. [8], and *Schistosoma* spp. [15]), as well as different approaches taken (natural or experimental infection, types of sequencing method, and analysis approaches). Additionally, the direct impact of anthelmintic treatment on the gut microbiome is unclear. While some studies found differences following deworming treatment [10, 12], others have found no impact of treatment on gut microbiota profiles [13]. Other studies that examined anthelmintic albendazole effects on the gut microbiota utilize primarily 16S rRNA sequencing [9, 10, 13, 16, 17]. Hence, a large study incorporating metagenomic sequencing with helminth infection status, albendazole treatment, and additional control groups may provide greater insights into these complex interactions.

Most of the helminth studies mentioned above utilized 16S rRNA sequencing to characterize the gut microbiota, while shotgun metagenomic approaches enable higher taxonomic resolution, at the species or strains level, and can identify not only bacteria but also archaea, fungi, and viruses [18, 19]. However, incomplete reference databases make it a challenge to profile uncharacterized microorganisms. Recently, an approach to assembling sequencing reads into contigs and binning them into putative genomes, known as metagenome-assembled genomes (MAGs), has enabled retrieving semi-complete genomes directly from samples without the need of culturing organisms [20, 21]. The Unified Human Gastrointestinal Genome (UHGG) established an integrated catalog of prokaryotic genomes containing 204,938 nonredundant genomes that represent 4644 prokaryotic species [3] by combining recent studies with large-scale assembly of MAGs from human microbiome data [3, 21, 22] as well as two culture-based studies that sequenced genomes from cultivated human gut bacteria [23, 24]. The Human Reference Gut Microbiome (HRGM) catalog expanded on UHGG to include underrepresented Asian metagenomes from Korea, India, and Japan [25] and added 780 new species from the newly assembled genomes [25]. However, Southeast-Asian countries remain underrepresented.

In this study, we generated shotgun metagenomics data from 650 Malaysian stool samples to investigate helminth-gut microbiome interactions by both cross-sectional and longitudinal analyses. The large sample size allowed us to examine these interactions in five different villages from different locations with different lifestyles. Examination of anthelmintic-treated uninfected individuals in the longitudinal phase enabled assessment of albendazole effects on the gut microbiome independent of helminths. Metagenomic data enabled investigation on the replication rates of individual bacterial species under different conditions. Since a substantial quantity of the microbiome remains undescribed, this large dataset from indigenous populations with traditional lifestyles from the underrepresented South East Asian region provides new insights into helminth-gut microbiome interactions and more comprehensive metagenomic sequences for future human gut microbiome studies.

## Results

### Gut microbiome analysis of indigenous Malaysians and urban controls

To identify and characterize helminth-associated microbiome effects, this study consisted of a cross-sectional component that compares urban individuals (*n* = 56) living in Kuala Lumpur (KL) with indigenous Orang Asli (OA) (*n* = 351) from five different villages (Figs. S1 and S2), as well as a longitudinal component to examine changes to the microbiome after anthelmintic (albendazole) treatment. A total of 650 fecal samples (including longitudinal samples) were processed for metagenomic sequencing, resulting in 11,480,206,516 paired reads after quality control and contamination removal (Supplementary Fig. S3). We compared different OA villages, which have different prevalence of soil-transmitted helminth infections (Supplementary Figs. S1 and S2). In the longitudinal phase of the study, consented OA subjects were treated with 400 mg albendazole for 3 consecutive days after collection of the first fecal sample. At 21 and 42 days following treatment, additional fecal samples were collected; however, this phase of the study was disrupted by the COVID-19 pandemic, reducing the number of paired samples available. KL subjects were not treated with albendazole, and they provided only one sample. Questionnaire data were collected and analyzed for some of the study subjects (*n* = 340).

**Fig. 1:**
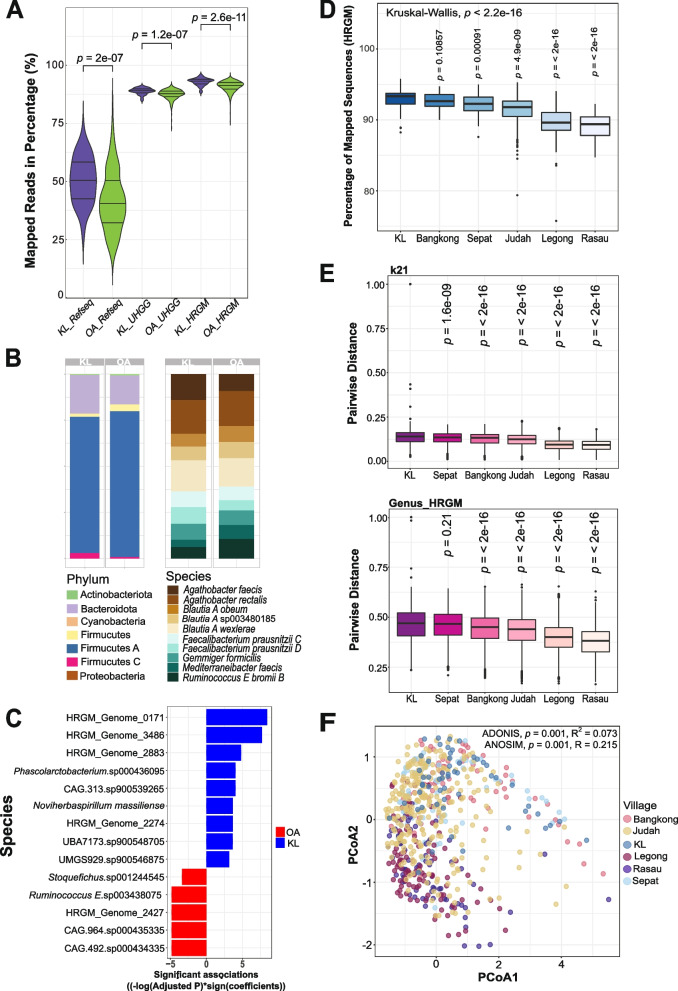
Variation in the gut microbiome of 650 Malaysians from Orang Asli (OA) villages and Kuala Lumpur (KL). **A** Violin plots illustrating the percentage of mapped reads with RefSeq (i.e., bacteria, protozoa, fungi, viral, archaea), Unified Human Gastrointestinal Genome (UHGG), and human reference gut microbiome (HRGM) databases between OA (green) and KL (purple) samples. **B** Relative abundance of phyla from the 237 species of the core gut microbiota of OA and KL populations (left). The relative abundance of the main species from Firmicute A (right). **C** Bar plot shows the bacterial species that are differentially abundant between Orang Asli and Urban cohort from Kuala Lumpur based on the output of the microbiome multivariable association with linear models 2 (MaAsLin2). The length of the bar corresponds to the value of the significant association. Red color represents the bacterial species associated with the Orang Asli subjects, whereas blue color represents the bacterial species associated with the urban cohort. **D** The percentage of mapped reads to the HRGM database for samples from different OA villages and KL. **E** Comparison of pairwise beta diversity at species level within group to the KL cohort, assessed by Jaccard distance based on the distance of nucleotide *k*-mer sketches *k* = 21 (top) and genus-level classification (bottom). **F** Principal coordinates analysis (PCoA) of Jaccard distance based on the gut metagenomic profiles (species levels) in all samples, with individuals from different geographical locations denoted by specific color (ADONIS: *p* = 0.001, *R*^2^ = 0.073; ANOSIM: *p* = 0.001, *R* = 0.215). The *p*-values for A, D, and E are computed using Wilcoxon rank-sum test

When we first mapped the metagenomic sequences to RefSeq (i.e., bacteria, protozoa, fungi, viral, archaea genomes), we observed a very low percentage of mapped reads (median: 41.6%). However, when we mapped the sequences to databases that incorporate MAGs (i.e., HRGM [25] and UHGG [3], the percentage of sequencing reads mapped to HRGM (median: 91.5%) and UHGG (median: 87.9%) was much higher than RefSeq (Fig. 1A). Additionally, the percentage of mapped reads to all three databases was higher in KL subjects than the OA population (HRGM: *p* = 2.6e^−11^; UHGG: *p* = 1.2e^−07^; RefSeq: *p* = 2e^−07^) (Fig. 1A), indicating that there are more unknown microbial genomes in the OA population.

Utilizing HRGM, we determined the core microbiota for the Malaysian population and found that 237 core bacterial species were 100% shared among the subjects (Fig. 1B; Supplementary Figs. S4 A–E and S5 A–C). The most abundant phylum was Firmicutes A, the majority of which were uncultured species [3] (Fig. 1B). *Agathobacter rectalis*, *Blautia_A wexlerae*, and *Agathobacter faecis* were the main species from Firmicutes A (Fig. 1B). Using a cross-validated random forest model to identify core microbiota species driving the variation between OA vs KL subjects, we achieved a mean prediction accuracy of 98.05% at a kappa of 96.06% (out-of-bag error = 1.8%). *Megamonas funiformis*, *Phocaeicola plebeius A*, *Bacteroides stercoris*, *Phocaeicola massiliensis*, and HRGM Genome 3145 were the top five predictors between OA and KL subjects (Supplementary Fig. S6 A–C). Of these, HRGM Genome 3145, *Gemmiger* sp900539695, and *Blautia A* sp000436615 were more abundant in OA subjects, while *Megamonas funiformi*, *Phocaeicola plebeius A*, and *Bacteroides stercoris* were more abundant in KL subjects (Supplementary Fig. S6 A–C). The bacterial species with the largest variation (cutoff 6.0 for the coefficient of variation) among the core gut microbiota is shown in Supplementary Fig. S7A. To control for covariates, we utilized (MaAsLin2) to identify bacterial taxa differentially abundant between OA and KL subjects that are independent of village, age, and sex. Fourteen bacterial species, of which many are uncharacterized, including HRGM_Genome_2427 (*p* = 0.009), CAG 964.sp000435335 (*p* = 0.009), and *Ruminococcus_E* sp003438075 (*p* = 0.009), are more abundant in OA subjects, whereas HRGM_Genome_0171 (*p* = 2e^−4^) and HRGM_Genome_3486 (*p* = 0.009) are more abundant in KL subjects (Fig. 1C and Supplementary Table S1).

The Orang Asli live in different geographical settings and have distinctive cultures and lifestyles. We found that KL subjects have higher mapped reads than all OA villages (Fig. 1D; Supplementary Fig. S7 B and C), and the percentage of mapped reads from both villages Rasau (*p* = 2e^−16^) and Legong (*p* = 2e^−16^) was markedly lower compared to KL (Fig. 1D; Supplementary Fig. S7 B and C). To compare pairwise beta diversity at the species level within each village group to the KL cohort and to use a reference independent strategy as an alternative approach, we assessed Jaccard distances using 21 nucleotide *k*-mers and genus-level annotations from HRGM, which showed similar results (Fig. 1E). In addition, we observed that Rasau and Legong had the highest beta diversity and nucleotide dissimilarity compared to KL (Fig. 1E). Moreover, comparison of bacterial communities at species level across geographical locations using Jaccard distance revealed substantial differences between villages (ADONIS: *p* = 0.001, *R*^2^ = −0.073; analysis of similarity [ANOSIM]: *p* = 0.001, *R* = 0.215) (Fig. 1F, Supplementary Table S2). From the principal coordinate analysis (PCoA) plot (Fig. 1F), we observed clustering of the samples from Rasau and Legong. Conversely, the samples from Bangkong and Sepat were clustered together with KL, while Judah exhibited a more dispersed distribution. Hence, OA subjects in Rasau and Legong were more similar in gut microbial composition and were different from KL and other villages. Equivalent beta-diversity results were observed with other *k*-mers sketches (31 and 51) and at the species level (Fig. S8 A–G).

### Village-dependent effects of helminth infection on the gut microbiome

**Fig. 2:**
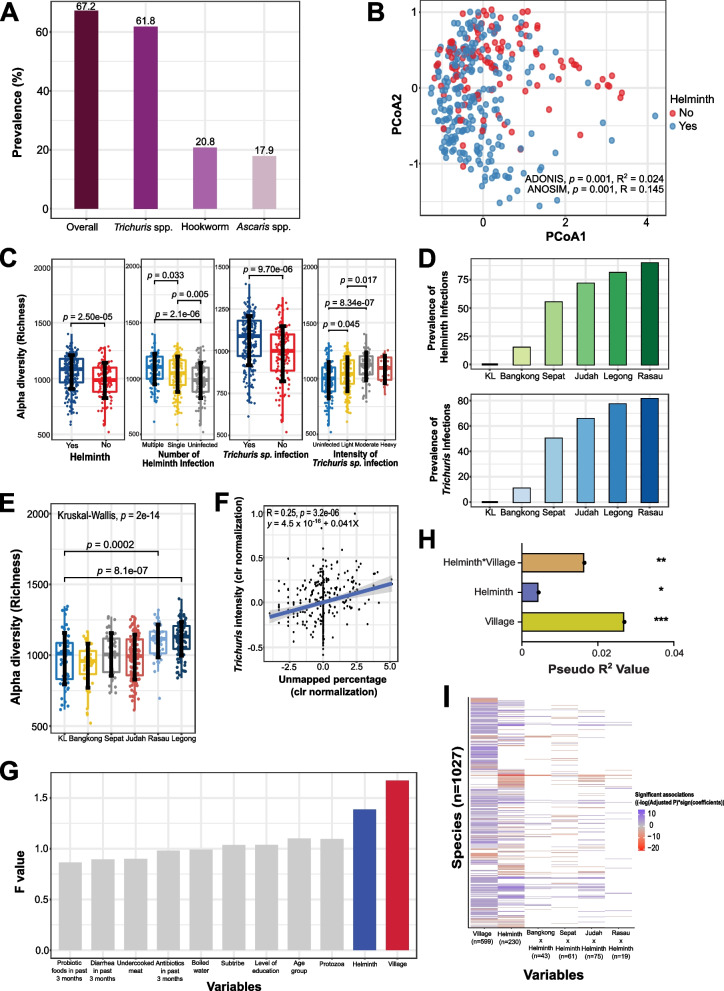
Effects of intestinal helminth infection status on gut microbial diversity and composition for the 351 Orang Asli individuals. **A** The prevalence of intestinal helminth infection in the OA cohort based on overall infection status, as well as specific intestinal helminth infection (i.e., trichuriasis, ascariasis, and hookworm infection). **B** Principal coordinates analysis (PCoA) of Jaccard distances based on gut microbiota profiles (species levels) of the OA cohort. The individuals infected and uninfected with intestinal helminths are denoted by blue and red, respectively (ADONIS: *p* = 0.001, *R*^2^ = 0.024; ANOSIM: *p* = 0.001, *R* = 0.145). **C** Alpha-diversity box plot of species richness based on different status of intestinal helminth infection, number of intestinal helminth infection, *Trichuris* infection, and intensity of *Trichuris* infection. Wilcoxon rank-sum test is used for two independent variables, while the Kruskal-Wallis test is used for more than two comparison groups. **D** The prevalence of intestinal helminth infection (top) and *Trichuris* infection (bottom) by different geographical locations. **E** Comparison of alpha diversity (species richness) between individuals from KL and specific OA villages. **F** Spearman correlation between the intensity of *Trichuris* infection and percentage of unmapped reads to the HRGM database (*p* = 3.200e^−6^, *R* = 0.250). The blue line represents the linear regression between intensity of *Trichuris* infection and percentage of unmapped reads. **G** Bar plot of the F statistic values from ADONIS analysis of variables that contribute to the gut microbiota composition. Colored bars indicate the variables that show significant effects on gut microbiota variation (*p* < 0.05). **H** Bar plot of effect size of the variables (village [*p* = 2.610e^−08^, pseudo *R*^2^ = 0.027], helminth infection [*p* = 0.029, pseudo *R*^2^ = 0.004], and interactions between helminth village [*p* = 0.002, pseudo *R*^2^ = 0.016]) that contribute significantly to the variance of the microbiota based on MDMR analysis. **I** Heatmap of bacterial species associated with village, helminth infections, and interactions from MaAsLin2. Blue for positive association and red for negative association

We determined the infection intensity and the prevalence of intestinal helminth infection among the 351 OA subjects and found that *Trichuris* infection (61.8%, *n* = 217) was the most predominant, followed by hookworm (20.8%, 73) and *Ascaris* (17.9%, 63) infections (Fig. 2A). The distribution of age and gender of these subjects is shown in Fig. S9 A and B. The overall prevalence of helminth infection was 67.2% (*n* = 236) (Fig. 2A), and infection intensity was summarized in Fig. S9C. For beta diversity at species level, based on PCoA, there were differences in gut microbiome between infected and uninfected individuals; however, statistically, the effect size was small (ADONIS: *p* = 0.001, *R*^2^ = 0.024; ANOSIM: *p* = 0.001, *R* = 0.145) (Fig. 2B, Supplementary Table S2), which was also the case for Bray-Curtis distance and nonmultidimensional scaling (NMDS) ordination (Supplementary Fig. S10 A–C).

For alpha diversity at species level, we observed higher species richness in the samples from infected subjects (*p* = 2.50e^−5^) (Fig. 2C). This relationship was confirmed by a linear mixed model analyses controlling for village as a random effect (*p* = 1.18 × 10^−6^). Individuals infected with either single (*p* = 0.005) or multiple species of helminths (*p* = 0.033) had higher species richness (Fig. 2C). *Trichuris*-infected OA (*p* = 9.70e^−06^) had higher species richness than uninfected (Fig. 2C), including those infected at light (eggs per gram [epg] < 999; *p* = 0.045) and moderate (*epg* < 9,999; *p* = 8.34e^−07^) intensities (Fig. 2C). Other alpha-diversity indices (i.e., Shannon and Simpson, at species level as well) are shown in Supplementary Fig. S11 A–H, and results for each village are shown in Supplementary Fig. S12 A–E. The prevalence of helminth infection varied according to village and was highest in Rasau (89.6%, *n* = 43 of 48), followed by Legong (81.0%, 81 of 100), Judah (71.6%, 83 of 116), Sepat (55.0%, 22 of 40), and Bangkong (14.9%, 7 of 47) (Fig. 2D). As *Trichuris* was the predominant helminth, the prevalence of *Trichuris* was similar for Rasau (81.3%, 39 of 48), Legong (77.0%, 77 of 100), Judah (65.6%, 76 of 116), Sepat (50.0%, 20 of 40), and Bangkong (10.6%, 5 of 47) (Fig. 2D). There was no infected individual with helminths in KL. The two villages with the highest prevalence, Rasau (*p* = 2.0e^−4^) and Legong (*p* = 8.1e^−07^), showed higher species richness compared to KL (Fig. 2E). Also, we observed that species richness appeared to be greater when helminth infections in the villages were more prevalent, which was similar to the order of villages for unmapped reads shown in Fig. 1D. To determine if *Trichuris* infection intensity was associated with unmapped reads, we performed a Spearman correlation test and found that the intensity of *Trichuris* infection was positively correlated (*p* = 3.2e^−06^, *R* = 0.25) with the percentage of unmapped reads to the HRGM database (Fig. 2F). These results indicated that helminth infections were associated with underrepresentation in the catalog of bacterial genomes.

We next determined the relative contribution of village and helminth infection status on the gut microbiome in relation to other factors (e.g., whether they had probiotic food, diarrhea, or antibiotics in the past 3 months, different age groups, and protozoa infection). ADONIS analysis at species level indicated that only village (*p* =1.000e^−4^, *F*-value = 1.672, *R*^2^ = 0.025) and helminth status (*p* = 0.028, *F*-value = 1.387, *R*^2^ = 0.010) had significant effects on the gut microbiome composition (Fig. 2G). Since village has the largest effect size on gut microbiome composition, we next used MaAsLin2 [26] to identify bacterial species that were differentially abundant between *Trichuris* infected and uninfected individuals from specific villages. Importantly, we found that the bacterial species that were most differentially abundant between infected and uninfected subjects were unique to each village (Supplementary Fig. S13A). For example, *Haemophilus_A.parahaemolyticus* and *Corynebacterium provencense* were different in Bangkong and Rasau, whereas *Prevotella*.sp900316565 was different in Sepat, C941.sp004557565 and UBA10281.HRGM_Genome_2392 in Judah, *Prevotella.*sp900546575 and *Prevotella.*HRGM_Genome_3676 in Legong, and UBA1829.sp900549045 and F082.HRGM_Genome_5331 in Rasau (Supplementary Fig. S13A). Similar patterns of results were obtained with ANCOM-BC (Supplementary Fig. S13B). These results indicated that helminth infections may have different effects on the gut microbiome in different villages.

To specifically test the hypothesis that effects of helminth infection on the microbiome are highly dependent on village, we used multivariate distance matrix regression (MDMR) [27] to test for statistical interactions between helminth infection and village and to calculate relative effect sizes on microbiota variation at species level. We find that there was a significant interaction between village and helminth infection, and that the effect of village is greater than helminth infection status after accounting for the effects of this interaction (Fig. 2H).

To identify bacterial features that are significant in helminth-village interactions, as well as independent of these covariates, we used MaAsLin2 with helminth and village as fixed effects, to identify bacteria that are independent and associated with the interaction between helminth and village. Of the 230 helminth-associated bacteria, more than 55% (*n* = 135) were associated with village (Fig. 2I). Hence, most of the effects of helminth-associated bacteria are village dependent, and in different villages, there are different bacteria associated with helminth infections. Several *Lactobacillus* species, including *Lactobacillus gasseri* and *Lactobacillus crispatus*, were associated with helminth infection independent of village (Supplementary Fig. S14 and Table S3).

### Dynamic changes to the gut microbiome after anthelmintic treatment

**Fig. 3:**
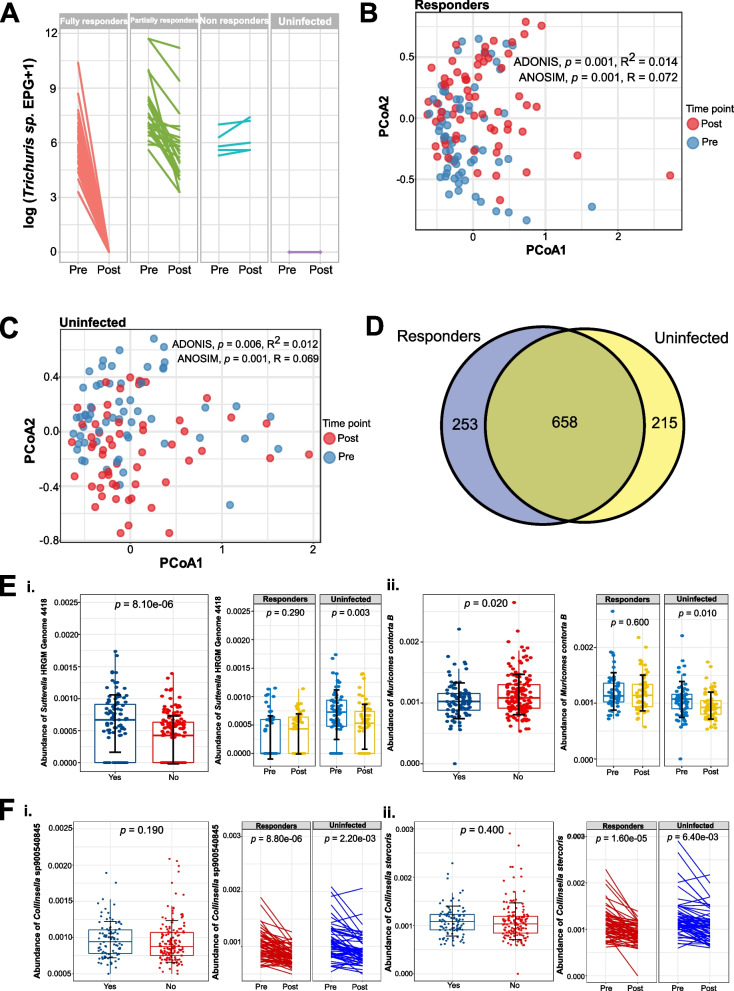
Dynamic changes to the gut microbiota of 129 Orang Asli after albendazole treatment. **A** Line plots show changes of the infection intensity of *Trichuris* pre and post response to anthelmintic drugs stratified by full responders (*n* = 43), partial responders (*n* = 23), nonresponders (*n* = 5), and uninfected individuals (*n* = 58). **B** Principal coordinates analysis (PCoA) plot of Jaccard distances based on gut microbiota profiles (species levels) of responders (ADONIS: *p* = 0.001, *R*^2^ = 0.014; ANOSIM: *p* = 0.001, *R* = 0.072), with pre-anthelmintic treatment (blue) and post-anthelmintic treatment (red). **C** Principal coordinates analysis (PCoA) plot of Jaccard distances based on gut microbiota profiles (species levels) of uninfected subjects (ADONIS: *p* = 0.006, *R*^2^ = 0.012; ANOSIM: *p* = 0.001, *R* = 0.069) (Fig. 3C, Supplementary Table S2), with pre-anthelmintic treatment (blue) and post-anthelmintic treatment (red). **D** Venn diagram depicting the number of shared and exclusive bacteria species that are found to be differentially abundant (pre and post) between responders and uninfected individuals. The blue area includes 253 bacteria that are altered only in responders, while the yellow and mixed color area indicates the 873 bacteria that are altered in uninfected individuals. **E** Box plots show the bacterial taxa that are altered by deworming treatment in responders but not in nonresponders (host response). The differences in the abundance of (i) *Sutterella HRGM Genome 4418* and (ii) *Muricomes contorta_B* in helminthic infections (left) and different groups of response (right), namely uninfected and responders in pre and post. **F** Box plots and line plots show the bacterial taxa that are associated with deworming treatment in both responders and nonresponders (drug response). The differences in the abundance of (i) *Collinsella* sp900540485 and (ii) *Collinsella stercoris* in helminthic infections (left) and different groups of response (right), namely uninfected and responders in pre and post. The *p*-values for E and F are computed using Wilcoxon signed-rank test (responders vs nonresponders) and Wilcoxon rank-sum test (helminthic infections)

Longitudinal interventional approaches provide stronger assessment of cause-and-effect relationships. Fecal samples analyzed at pre- and post-anthelmintic treatment provided insights into the effects of deworming on the gut microbiome. Individual subjects were grouped into four categories (i.e., full responders [*n* = 43 paired; from 26–33,099 to 0 epg], partial responders [*n* = 23 paired; from 281–119,875 to 26–71,579 epg], nonresponders [*n* = 5 paired; from 204–1097 to 281–1632 epg], and uninfected [*n* = 58 paired]), based on the *Trichuris* infection intensity before and after deworming (Fig. 3A). While mixed infection was present in some individuals, hookworm and *Ascaris* infection were always cured after deworming (Supplementary Fig. S15A).

First, we compared pre and post samples for responders, which include both full and partial responders. PCoA based on Jaccard distances showed that there are differences in gut microbiota composition at species level between pre and post treatment, but the effect size was small (ADONIS: *p* = 0.001, *R*^2^ = 0.014; ANOSIM: *p* = 0.001, *R* = 0.072) (Fig. 3B, Supplementary Table S4). Since albendazole may have a direct effect on the microbiota, we next compared the gut microbiota profile pre and post treatment for uninfected individuals. Similar to the responders, PCoA based on Jaccard distances also indicated differences in gut microbiota composition at species level between pre and post samples, with a small effect size (ADONIS: *p* = 0.006, *R*^2^ = 0.012; ANOSIM: *p* = 0.001, *R* = 0.069) (Fig. 3C, Supplementary Table S4). NMDS ordination, Bray-Curtis distance matrix, and beta-dispersion analysis showed similar results (Supplementary Figs. S16 A–E and S17 A–E, Table S4), and there were no significant changes to alpha diversity at species level between pre and post treatment (i.e., Richness, Shannon, Simpson) (Supplementary Fig. S15 B and C).

Using MaAsLin2 for differential abundance testing, we found changes of 911 bacterial species at pre and post treatment among responders. However, there was substantial overlap with changes found in pre and post treatment samples for uninfected individuals (658 species, 72.2%) (Fig. 3D and Supplementary Fig. S18A), with only 253 taxa which were specific to the responders. For example, in both responders and uninfected individuals, the relative abundance of *Collinsella* sp003466125 (*p* = 1.52e^−08^; *p* = 3.66e^−07^, respectively) and RUG013.sp001486445 (*p* = 2.53e^−07^; *p* = 3.80e^−06^) was reduced after deworming, while the relative abundance of *Bilophila* sp900550745 increased (*p* = 1.33e^−08^; *p* = 5.81e^−05^) (Supplementary Fig. S18 B and C). To assess the longitudinal effects of albendazole treatment, we also used MaAsLin2 to identify taxa altered by treatment response, controlling for infection status and village as fixed effects. Of the 576 species that were identified to be associated with these covariates, the majority were associated with village (*n* = 305) and with infection status (*n* = 200), and only 69 species were associated with treatment response, of which only four species were independent of village and infection status (Supplementary Fig. S19A). Of the four, only one (CAG.245.sp900552135) showed a statistically significant (Supplementary Fig. S19B) association with treatment response but independent of village or infection status (Supplementary Table S5).

Next, we used MaAsLin2 to identify bacterial taxa that were associated with drug response and helminth status, correcting for village as a covariate. There were a total of 293 bacteria species that were associated with drug response, only six of which were associated with host response (Supplementary Fig. S19C). There were only two taxa (*Sutterella* HRGM Genome 4418 and *Muricomes contorta* B) associated with deworming treatment in responders but not in nonresponders in this model. The *Sutterella* HRGM Genome 4418 (*p* = 8.10e^−06^) was more abundant in helminth-infected individuals, whereas *Muricomes contorta* B (*p* = 0.024) was more enriched in nonhelminth-infected individuals (Fig. 3E). In contrast, there were many more taxa (*n* = 295) that are associated with deworming treatment in both responders and nonresponders like *Collinsella* sp900540845 and *Collinsella stercoris* (Fig. 3F), which indicates that the effects of albendazole were greater on the bacterial communities than helminth infection itself (Supplementary Table S6). Hence, albendazole may have a substantial effect on the microbiota that may be an important confounding factor for deworming studies.

In some individuals, we conducted a follow-up study 42 days post-anthelmintic treatment. There were no differences in alpha diversity on day 42 (Supplementary Fig. S20A), and although beta-diversity analysis at species level showed significant differences between three timepoints (i.e., pre, 21 days, and 42 days) (Supplementary Fig. S20 B–C, Supplementary Table S7), these differences are driven by the pre-treatment samples (Supplementary Fig. S20D). Therefore, the changes in the gut microbiome in both responders and uninfected individuals after albendazole treatment remain stable by day 42.

### Bacterial replication in the context of helminth infection

**Fig. 4:**
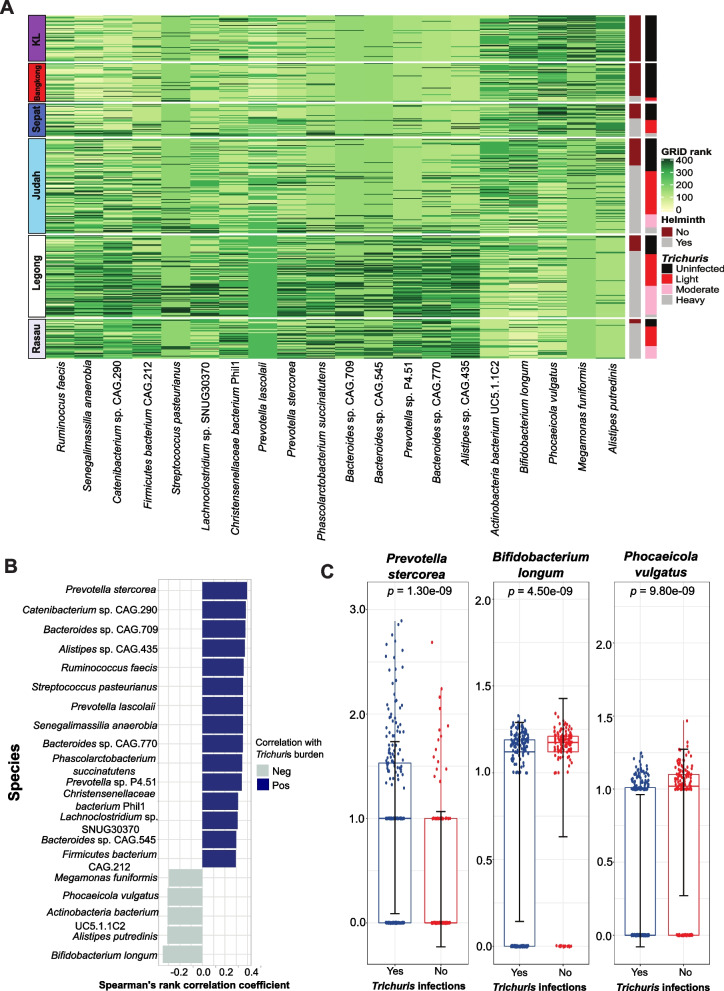
Gut bacterial replication in the context of intestinal helminth infection. **A** Heatmap of the growth rate index (GRiD) score, which infers an index of replication for top 20 gut bacteria in relation to helminth infection status of individuals based on Spearman correlation test followed by false discovery rate (FDR) correction. Samples are shown in rows, by village, whereas the GRiD score of each bacterium is shown in columns. The first vertical side bar color codes the intestinal helminth infection status, while the second side bar indicates the infection intensity of *Trichuris*. **B** GRiD score correlation between bacterial species with the infection intensity of *Trichuris*. The bar chart shows the Spearman’s rank correlation coefficient. Blue and gray colors represent the positive and negative correlations respectively. **C** Box plots of GRiD score for *Prevotella stercorea* (left), *Bifidobacterium longum* (middle), and *Phocaeicola vulgatus* (right) in *Trichuris* infected and uninfected individuals. The GRiD scores of these species between *Trichuris* infected and uninfected individuals were tested using Wilcoxon rank-sum test

Actively replicating bacteria can be identified by calculating the index of replication based on coverage trends of bidirectional genome replication from a single origin of replication. We used the algorithm growth rate index (GRiD) to estimate the growth rate of gut bacteria in relation to helminth infection status. Spearman correlation analysis on *Trichuris* egg burden with the growth rate of the bacteria identified 350 bacterial species with growth rate associated with *Trichuris* egg burden (Fig. 4 A and B, Supplementary Fig. S21, and Supplementary Table S8). *Prevotella stercorea* replication was most positively associated (*p* = 1.58e^−14^, *R* = 0.39) with egg burden, while *Bifidobacterium longum* (*p* = 1.30e-11, *R* = −0.35) and *Phocaeicola vulgatus* (*p* = 3.45e^−9^, *R* = −0.31) were negatively associated with egg burden. Using a linear mixed model of *Trichuris* egg burden while controlling for village, *Prevotella stercorea* and *Bifidobacterium longum* were significantly associated with egg burden (*p* = 4.89e^−06^ and *p* = 7.99e^−08^, respectively). The predicted replication rate of *Prevotella sterorea* was higher in *Trichuris*-infected individuals (*p* = 1.30e^−09^), while the predicted replication rate of *Bifidobacterium longum* and *Phocaeicola vulgatus* was notably lower in *Trichuris*-infected individuals (*p* = 4.50e^−09^and *p* = 9.80e^−09^, respectively) (Fig. 4C).

For the longitudinal deworming component of the study, we observed that the growth rate of 93 bacterial species was different between pre and post treatment samples among the responders. Among these bacterial species, slightly more than one-third of them (*n* = 33) were also identified from the cross-sectional analysis (Supplementary Fig. S22A). Spearman correlation analysis demonstrated that the growth rate of uncultured *Oscillibacter* sp. (*p* = 6.000e^−04^) and *Phocaeicola vulgatus* (*p* = 0.006) was significantly correlated with *Trichuris burden* (Supplementary Fig. S22B and Supplementary Table S9). After we verified the results by building a linear mixed model controlling for village, both *uncultured Oscillibacter* sp. (*p* = 0.033) and *Phocaeicola_vulgatus* (*p* = 0.028) were negatively associated with *Trichuris* burden, indicating more replication in responders after anti-helminthic treatment (Supplementary Fig. S22C). However, considerable portions of responder-associated taxa (32 out of 93) were also observed in the noninfected individuals (*n* = 67) (Supplementary Fig. S22D). Therefore, it could be difficult to disentangle the effects of *Trichuris* infection and direct effects of albendazole treatment on the dynamics of the microbiome. Hence, we conducted MaAsLin2 analyses to identify bacterial replication (based on GRiD score) that were associated with *Trichuris* infection while controlling for treatment group and village. *Collinsella*_sp._TF06.26 (adjusted *p* = 0.004) was positively associated with *Trichuris* infection, while *Phocaiecola vulgatus* (adjusted *p* = 0.04), *Burkholderia* sp. K4410.MGS.135 (*p* = 0.04), *Bacteroides stercoris* (adjusted *p* = 0.04), and *Phocaeicola massiliensis* (adjusted *p* = 0.04) were negatively associated with *Trichuris* infection (Supplementary Table S10).

### Functional gene profiles of the Orang Asli microbiota and the effects of albendazole treatment

We used the HUMAnN tool to investigate pathway inference and gene families with Pfam domains (Figs. S23–S25). To adjust for covariates, we used MaAsLin2 to identify the pathways and gene families that were differentially abundant between the following: (1) Orang Asli and Urban cohorts, while controlling for age and sex; (2) Helminth positive and negative individuals, while including village as a covariate; and (3) Treatment response groups while controlling for village. Using these models, we find that the L-tryptophan biosynthesis superpathway was enriched in the Orang Asli microbiome compared to urban controls from KL (Supplementary Figs. S23 and S25A). Tryptophan, an essential amino acid, and its catabolites have been suggested to affect intestinal homeostasis through the aryl hydrocarbon receptor and may be important in inflammatory bowel diseases [28]. Hence, future work on microbial metabolism in the Orang Asli may focus on this pathway.

After controlling for village, there were no significant pathways differentiating helminth positive and negative individuals. Between different villages, we found the strongest significance for the peptidoglycan biosynthesis II (Staphylococci) pathway (Supplementary Figs. S23 and S25B). Villages with high helminth prevalence have individuals enriched in this pathway, but we did not find a significant relationship between helminth infection and *Staphylococcus aureus* abundance. This pathway may be important in the generation of peptidoglycan in other gram-positive bacteria, and the significance of the geographical difference in the abundance of this pathway is still unclear. We also found that the microbiome after albendazole treatment is enriched for the L-glutamate degradation V (via hydroxyglutarate) pathway (Supplementary Figs. S23 and S25C), which is an indication that albendazole may affect the fermentation of amino acids in an anoxic environment. Additionally, in our gene family enrichment analysis, our only substantial observation is that the phosphoenolpyruvate carboxylase gene family was decreased after albendazole treatment (Supplementary Figs. S2 and S25D). This also indicates how albendazole can affect metabolic processes of the microbiome; however, the implications of these results remain unclear.

## Discussion

In this study, we examined 650 stool metagenomes from a cohort of 351 indigenous Malaysians from five villages with different prevalence rates (14.9–89.6%) of helminth infections, along with 56 urban citizens (uninfected) living in Kuala Lumpur City. To our knowledge, this is the largest study utilizing shotgun metagenomics to investigate the interactions between helminth infection and the human gut microbiome.

We found that mapping reads to the HRGM database, which incorporates MAGs, increase the quality and quantity of taxonomic classifications, compared to using from the NCBI database alone, especially for the indigenous Orang Asli. We also found that the microbiota is dominated by Firmicutes A, which is represented by mostly uncultured bacteria, highlighting the underrepresentation of cultured bacteria from indigenous groups. This could be an important caveat for most of the previous studies on helminths and the gut microbiota, which were conducted using 16s rRNA sequencing [10–12, 16, 29] with the taxonomic classification based on mapping to the reference databases Greengenes, SILVA, and Ribosomal Database Project. In a recent shotgun metagenomic study on 175 Cameroonian samples, the data was also mapped onto a reference database from NCBI [30], and the investigators noted that the classification of the relative abundance of bacteria did not correspond to data from 16S Greengenes classifications for the V4 region [30]. A different study also indicated that 16s rRNA gene sequencing only provided a portion of the gut microbiota profile compared to shotgun metagenomics [31]. Hence, we suggest that assembling a Malaysian gut microbiome reference catalog will provide substantial benefit for future microbiome studies, especially from underrepresented geographic regions, and for rural and indigenous populations.

In this metagenomic study, we found that intestinal helminth infection status was associated with higher species richness, which was consistent with our previous findings and others conducted using 16s rRNA sequencing [9, 11, 12, 16, 30, 32]. However, we did not find a significant difference at pre and post deworming, which could be because of smaller sample size and could also be confounded by the effects of albendazole. Additionally, other studies have not observed an effect of helminths on microbial diversity [7, 10, 13, 16, 17, 33]. It is important to note that each study cohort has different prevalence rates for different helminth species, as well as distinct genetics, lifestyles, and living conditions. This study has a larger sample size than our previous studies [12, 34] and has enabled us to examine the interactions of helminth infection and the gut microbiome in different villages. Indeed, village has the largest effect size on gut microbiome variation, followed by helminth infection status. Notably, villages with higher helminth prevalence rates also have higher microbial diversity, but in different villages, helminth infection is associated with differential abundances of distinct bacterial taxa. It is important to note that the different villages represent diverse environments, practicing unique lifestyles, and have different hygiene practices. Compared to other villages, Rasau and Legong villages (with higher helminth prevalence) are located near the forest with high exposure to the soil environment, which may harbor more microbes [35], and mouse experiments have shown that exposure to soil increases gut microbiota diversity [36]. From our questionnaire, a higher percentage of villagers from Rasau and Legong are plantation agricultural workers (Rasau: 30.4%; Legong: 14.8%; others: < 6.7%), lack of toilet facility (52.5%; 20.8%; < 13.0%), and practice open defecation (46.5%; 32.0%; < 7.4%) more than other villages. As *Trichuris* eggs become infective in the soil, this may increase exposure to *Trichuris*, as well as other microbes in the contaminated soil, resulting in higher microbial diversity in different settings.

We also found that deworming helminth-negative individuals can influence the gut microbiome that overlaps substantially with changes in individuals responding to drug treatment by having reduced worm burdens. This indicates that albendazole may directly affect the microbiome, or that there are population effects that can influence uninfected people. There are four previous studies on albendazole [9, 10, 13, 16]. The first study conducted among Ecuador school children did not find any difference in bacterial composition among both *Trichuris* infected and uninfected groups after a combination of albendazole and ivermectin treatment [13]. In contrast, the second study in Indonesia found an increase of *Actinobacteria* and decrease in *Bacteroidetes* with albendazole treatment versus placebo in individuals that remained helminth-infected post treatment, but not in uninfected individuals [16]. In addition, Rosa et al. demonstrated that the gut bacterial composition was altered in a helminth-uninfected group in Indonesia after 2 years [9]. Another study in Kenya found significantly reduced Chao richness in uninfected individuals after deworming treatment, suggesting an effect of albendazole [10]. Albendazole is a prodrug that metabolizes rapidly to albendazole sulfoxide (the active anthelmintic compound) and albendazole sulphone (the inactive compound). Some bacterial species (*Enterobacter aerogenes* NCIM 2695, *Klebsiella aerogenes* NCIM 2258, *Pseudomonas aeruginosa* NCIM 2074, and *Streptomyces griseus* NCIM 2622) could be involved in metabolizing albendazole to albendazole sulfoxide and albendazole sulphone [37]. Albendazole can also be metabolized by the resident microbiota in gut rumens in sheep and cattle [38]. Hence, the gut microbiota could play a crucial role in metabolizing albendazole and influence drug bioavailability and efficacy on infected individuals. Why albendazole has lower efficacy against *Trichuris* infection than hookworm and *Ascaris* warrants further investigation [39]. Future studies could apply metabolomics profiling to investigate metabolite differences between response groups to better understand the underlying mechanisms. The observation that the L-glutamate degradation V (via hydroxyglutarate) pathway was enriched after albendazole treatment and that the phosphoenolpyruvate carboxylase gene family was depleted after albendazole treatment indicates that albendazole can affect metabolic processes, and future work should focus on the effects of albendazole on microbial metabolism.

## Conclusions

We find that this metagenomic study of rural indigenous populations required reference databases that included MAGs to improve taxonomic and functional classification of sequencing reads. However, unmapped reads remain a challenge as villages with higher prevalence of helminth infections have more unmapped reads. Hence, this large metagenomic dataset from five different villages in Malaysia with different helminth infection prevalences should facilitate further characterization of microbiome-parasite associations in other nonindustrialized populations. Helminth effects on the microbiome were village dependent, and albendazole treatment had a substantial effect on the microbiome. These results may explain some of the discrepancies from previous studies on helminth-microbiota interactions.

## Methods

### Study design and sample collection

This study consists of both cross-sectional and longitudinal phases. Cross-sectional comparisons were made on the OA and between OA and urban cohorts (KL) living in the capital city of Malaysia, Kuala Lumpur. Within the Orang Asli community, we studied five Orang Asli villages: (1) Rasau village (Perak state); (2) Judah village (Selangor state); (3) Sepat village (Selangor state); (4) Bangkong village (Selangor state); and (5) Legong village (Kedah state). The locations of each village are displayed on a map using ArcGIS (version 10.7.1) together with other information including states, tribes, and subtribes (Supplementary Fig. S1). A total number of 351 samples were collected from Orang Asli subjects and 56 samples from KL subjects in this cross-sectional component (aged 4 years and older) (Supplementary Fig. S2).

For the longitudinal phase, Orang Asli subjects who provided consent were treated with 400 mg albendazole for 3 consecutive days after the first stool sample collection. Stool samples were collected from the treated subjects at 21 days and 42 days following anthelmintic treatment. However, due to the restriction during the COVID-19 pandemic, only four Orang Asli villages were included in this phase, excluding Legong village. There was no follow-up for urban controls after the cross-sectional phase because they were not treated with albendazole. Sample selection for analysis was based on a complete set of paired stool samples (pre [pre-anthelmintic treatment] and post [21-day post-anthelmintic treatment]) (*n* = 129) and three timepoints stool samples collection (pre, 21 days and 42 days; *n* = 110). Four subject samples were removed from the longitudinal analysis due to incomplete data collection. Then, subjects were categorized into three groups for comparison: responders, nonresponders, and uninfected, based on their infection status before and after the albendazole treatment. Responders (*n* = 66 paired samples) refer to individuals who were positive at baseline and became negative or showed reduction of infection intensity after deworming. Nonresponders (*n* = 5 paired samples) refer to individuals who were positive at baseline and showed increment or maintain of egg counts after deworming. Uninfected (*n* = 58 paired samples) refers to negative individuals before and after the treatment. Nonresponders were not included in the gut metagenome analysis due to insufficient sample size. The detailed number of samples collected at each timepoints was shown in Supplementary Fig. S2.

### Fecal sample preparation and analysis

All the stool samples collected were divided into two portions: (i) preserved in 2.5% potassium dichromate and stored at 4 °C for intestinal helminth infection screening and (ii) aliquoted in 1.5 ml cryovial tube, frozen immediately in dry ice, and kept at −80 °C for shotgun metagenomic analysis (Supplementary Fig. S26). To detect and quantify helminth infections, Kato-Katz was performed. A thick smear was prepared from the fresh stool according to the manufacturer’s instructions (Kato-Katz kit, Mahidol University, Thailand) [40]. Infection intensity was stratified into light, moderate, or heavy according to WHO cutoffs [41]. Formalin ether sedimentation was performed according to Chin et al. (2016) [42]. Stool samples were considered positive if any soil-transmitted helminths were detected from any of these two methods. DNA was extracted from stool samples using Qiagen DNeasy PowerSoil Pro Kit (Qiagen, Hilden, Germany). DNA library was prepared using Illumina TruSeq DNA Nano Library kit (Illumina, USA). Paired-end metagenomic sequencing was performed on the NovaSeq 6000 S4 platform to generate an average of 20 million paired-end reads per sample (range 13–35 million paired-end reads), with a read length of 150 bp and insert size of 350 bp.

### Sequencing analysis pipeline

The overall bioinformatic analysis workflow from preprocessing to downstream analysis is shown in Supplementary Fig. S3. In brief, the whole process of quality filtering and trimming of the raw sequence reads was performed by using KneadData (version 0.7.4) integrated with Trimmomatic [42], Bowtie [43], and FastQC [44] tools. Sequence reads were trimmed by using Trimmomatic with default settings, based on a sliding window trimming approach (SLIDINGWINDOW:4:20) when average base Phred quality score over four reads dropped below 33 (PHRED 33). Next, sequence reads were mapped against reads mapping to the reference genome (hg37) using Bowtie2 with default parameters (very sensitive end-to-end alignment) to remove human host genome. The filtered reads were then used for the downstream analyses. Additionally, FastQC was used to perform quality checks on the raw metagenomic reads before preprocessing and after preprocessing to ensure high-quality metagenomic reads for downstream analysis.

For taxonomic classification, Kraken2 (version 2.1.0) [45], a *k*-mer matching algorithm classifier, was used for assigning taxonomic labels to the trimmed reads. The trimmed reads were mapped using Kraken2 against (1) RefSeq database (bacterial, protozoa, fungi, viral, and archaeal) and two MAGs integrated databases: (2) HRGM database, with 232,098 reference genomes [25], and the UHGG database, with 204,938 reference genomes [3] using default settings. After taxonomic classification by Kraken2, Bayesian Re-estimation of Abundance with KrakEN (Bracken2) (version 2.6.0) [46] was used to compute the relative abundance of bacteria for each taxa (from phylum to species level) using default settings with a read length parameter of 150. The mapped reads of the OA and KL cohorts were then plotted into a violin plot using ggplot2 package [47] to access which databases provide better taxonomic classification. The distribution of the mapped reads was determined using the Shapiro test from the rstatix package [48]. Then, the difference between the mapped reads of OA and KL was determined using the Wilcoxon rank-sum test from ggplot2 package [47]. The data generated from Bracken2 were exported in the form of BIOM (Biological Observation Matrix) table and analyzed using R programming language (version 4.0.5, R Studio, Inc., Boston, MA, USA). The BIOM table was imported and filtered using the phyloseq package [49]. Only those taxa with a minimum prevalence of 20% across all the samples and a minimum coefficient of variation of 3.0 were included in the following analysis (Supplementary Fig. S27). In general, ggplot2 [47] and ggpubr package [50] were used to create visualization plots.

In order to confirm our findings, we performed reference independent strategy by using Sourmash (version 4.0.0) [51] to compute *k*-mer sketches. To discard erroneous *k*-mers, the low abundance of *k*-mers was trimmed using “trim-low-abun” from k-mer project, with a *k*-mer abundance cutoff of 3.0 and trimming coverage of 18. Signatures were generated for each sample using “sourmash compute” with a compression ratio of 10,000 (−scaled 10,000) and *k*-mer lengths of 21, 31, and 51 (−*k*21, −*k*31, −*k*51). A signature output was generated for Jaccard distance comparisons. Before the *k*-mer comparison, “sourmash index” was used to create a Sequence Bloom Tree database from a collection of signatures. Lastly, “sourmash compare” was used with default settings to compare the signatures at each length of *k*.

The core microbiota was determined by including taxa present across all samples (i.e., prevalence of 100% across all the samples). Then, the core microbiota was visualized using bar chart to compare the heterogeneity between OA and KL cohorts as well as the heterogeneity across different villages. Alpha diversity, in terms of species richness [52], Shannon [53], and Simpson index [54], was analyzed using the microbiomeSeq package [55]. Beta-diversity analysis was performed on both the Jaccard and Bray-Curtis dissimilarity matrix calculated from the taxon abundance data standardized using Hellinger. Differences in beta diversity between groups (i.e., different OA villages and different helminth infection status) or between different timepoints (pre vs post) were displayed with principal coordinates analysis (PCoA) plots and NMDS plots.

Metagenomes were annotated for functional genes and pathways using HUMAnN v3.0 and its UniRef 50, Pfam, and MetaCyc pathway databases using read sequences that were trimmed and quality filtered using KneadData [56]. Read counts were normalized for gene length (reads per kilobase), transformed by centered log ratio, and filtered to remove very low prevalent features before statistical analyses were carried out with MaAsLin2 v1.10 using the same linear-mixed effects models as for the taxonomic comparisons [26].

Growth rate index (GRiD) (version 1.3) was used to evaluate the growth rate of microbial species in metagenomic samples [57]. Samples were mapped to a GRiD database (ftp://ftp.jax.org/ohlab/GriD_environ_specific_database/stool_microbes.tar.gz), a stool-specific database created based on microbes mostly found in stool. GRiD score > 1.02 indicates bacteria are in growth phase, whereas GRiD score < 1.02 indicates that bacteria are in stationary or lag phase. The downstream analysis was conducted as described in Supplementary Fig. S28.

### Statistical analysis

Multiple efforts were done for the differential abundance analysis of the bacteria from OA and KL cohorts. Random forest (randomForest package) was used to identify microbiome taxa predictive of OA and KL [52] groups. We generated a “SMOTE” (Synthetic Minority Oversampling Technique) (consisting of 280 OA and 336 KL) dataset using the package DMwR [58] to address the imbalance number of samples between OA (*n* = 594) and KL (*n* = 56) samples [59]. SMOTE algorithm is a technique to address the imbalanced datasets by oversampling the minority class. A new data of the minority class was created artificially using the nearest neighbors of these cases and hence leading to a more balanced dataset [60]. Then, the random forest model was built based on this “SMOTEd” data set and tuned with the methods described by Brwonlee (2016) [61], followed by the significant testing using the methods described by Douglas (2020) [62]. Another more detail analysis via MaAsLin2 (Microbiome Multivariable Association with Linear Models2) from MaAsLin2 package of R was conducted to identify microbiome taxa predictive of OA and KL groups while controlled for village, age, and genders.

For alpha diversity, Wilcoxon rank-sum test [63] was performed to compare groups statistically in the cross-sectional study (i.e., helminth-infected vs Uninfected and OA villages versus the KL), whereas Wilcoxon signed-rank test was used for paired samples in longitudinal study (i.e., pre vs post for both responders and uninfected). We also conducted a linear mixed model to examine the impact of helminth on alpha diversity while controlling the village location [64].

As for beta diversity, the comparison on pairwise distance of the samples between OA villages and KL was conducted using Wilcoxon rank-sum test. This same analysis was also applied to the output generated from *k*-mers sketches. Permutational multivariate analysis of variance (PERMANOVA) under ADONIS function [65] from the vegan package was conducted with 10,000 permutations on both the Jaccard and Bray-Curtis dissimilarity matrix. This analysis was first performed on specific variables of interest (i.e., different geographical locations, helminth status, and pre vs post). ADONIS was used to assess the effect of multiple variables on the gut microbial composition (e.g., if they had probiotic food, diarrhea, and antibiotics drug in the past 3 months, different age groups, subtribes, and protozoa infections), as well as analysis of similarity (ANOSIM) [66]. To test for multivariate dispersions among groups, the permutation multivariate analysis of dispersion (PERMDISP) [65] was performed via the betadisper function and Tukey’s test under the vegan package [67].

The MDMR was used to specifically test for “statistical interactions” between helminth status and village location and to calculate the relative effect sizes on microbiome variation [27]. Differential abundance analysis was performed using MaAsLin2 [26] for both cross-sectional (i.e., helminth positive and negative individuals while including village as a covariate) and longitudinal data (i.e., between host and drug response while controlling for village as well as between responders and uninfected while controlling for treatment and helminthic infections). Analysis of composition microbiomes with bias correction (ANCOM-BC) [68] was also conducted to validate the output generated from MaAsLin2, for cross-sectional data only.

For GRiD analysis, Spearman’s rank correlation test was conducted to examine the association between the bacterial species growth rate with *Trichuris* infection intensity for cross-sectional study. Then, the results were corrected using Benjamini-Hochberg with a false discovery rate (FDR) of 5% [69]. As for the longitudinal study, comparison of bacterial species growth rate between pre and post treatment of both responders and uninfected was computed using Wilcoxon rank-sum test and corrected using Benjamini-Hochberg with a FDR of 5%. A linear mixed model was built via the lmertest package [64] to determine the impact of the *Trichuris* intensity on the specific microbial growth rate by controlling for the village location for both the longitudinal and cross-sectional studies. Besides, multivariate association with linear models 2 (MaAsLin2) [26] was used to determine the microbial growth rate with differentially abundance in helminthic infections while controlling for village location and treatments.

Overall, the Wilcoxon rank-sum test and Wilcoxon signed-rank test from the rstatix package [48] were used to determine the *p*-value between groups for specific taxa in cross-sectional and longitudinal study respectively.

### Key resources table

List of the bioinformatic tools and R packages used is displayed in the table below:

**Table:** 

Tasks/analysis	Name	Source	Identifier
**Bioinformatic tools**			
Quality control, filtering, and trimming of raw sequence	KneadData	No publication	https://github.com/biobakery/kneaddata
	Trimmomatic	Bolger et al., 2014 [70]	https://github.com/biobakery/kneaddata
	Bowtie2	Langmead et al., 2012 [43]	https://github.com/biobakery/kneaddata
	FastQC	Andrew, 2017 [44]	https://www.bioinformatics.babraham.ac.uk/projects/fastqc/
Taxonomy assignment	Kraken2	Wood et al., 2019 [45]	https://github.com/DerrickWood/kraken2/wiki#downloads
Estimate relative abundance of species or genera	Bracken2	Lu et al., 2017 [46]	https://github.com/jenniferlu717/Bracken
Compute hash sketches from DNA sequence	Sourmash	Brown et al., 2016 [51]	https://sourmash.readthedocs.io/en/latest/
Functional analysis	bioBakery 3	Beghini et al., 2021 [56]	https://github.com/biobakery/biobakery/wiki
Replication of bacterial species	GRiD	Emiola et al., 2018 [57]	https://github.com/ohlab/GRiD
	Pathoscope 2.0	Hong et al., 2014 [71]	https://github.com/PathoScope/PathoScope
**R packages**			
Data import, filtering, and processing	phyloseq	McMurdie et al., 2013 [49]	https://joey711.github.io/phyloseq/index.html
Core microbiota analysis	DMwR	Amunategui, 2014 [58]	http://amunategui.github.io/smote/
	randomForest	Breiman et al., 2018 [72]	https://cran.r-project.org/web/packages/randomForest/index.html
Alpha diversity	phyloseq	McMurdie et al., 2013 [49]	https://joey711.github.io/phyloseq/index.html
	microbiomeSeq	Ssekagiri et al., 2017 [55]	https://github.com/umerijaz/microbiomeSeq
	rstatix	Kassambara, 2021 [48]	https://cran.r-project.org/web/packages/rstatix/index.html
Beta diversity	phyloseq	McMurdie et al., 2013 [49]	https://joey711.github.io/phyloseq/index.html
Differential abundance analysis	MaAsLin2	Malick et al., 2021 [26]	https://huttenhower.sph.harvard.edu/maaslin/
	ANCOMBC	Lin et al., 2020 [68]	http://www.bioconductor.org/packages/release/bioc/vignettes/ANCOMBC/inst/doc/ANCOMBC.html
ADONIS and ANOSIM	vegan	Oksanen et al., 2020 [67]	https://cran.r-project.org/web/packages/vegan/index.html
Interaction between covariates	MDMR	McArtor, 2018 [27].	https://cran.r-project.org/web/packages/MDMR/index.html
	lmerTest	Kuznetsova et al., 2020 [64]	https://cran.r-project.org/web/packages/lmerTest/index.html
Correlation test	psych	Revelle, 2022 [69]	https://cran.r-project.org/web/packages/psych/index.html

## Supplementary Information

**Additional file 1: Table S1.** MaAsLin2 results of the bacterial taxa differentially abundant between OA and KL subjects independent of village, age, and sex as covariates. **Table S2.** Relative impact of village, helminth infection and *Trichuris* infection on gut microbiome dissimilarity across samples (ADONIS, ANOSIM, and Betadisper; permutation = 999) in the cross-sectional analysis. **Table S3.** MaAsLin2 results of the bacterial taxa that are independent and associated with the interaction between helminth and village. **Table S4.** Relative impact of deworming on gut microbiome dissimilarity of different group of Orang Asli samples based on their microbiome data in pre and post anthelmintic treatment (ADONIS, ANOSIM, and Betadisper; permutation = 999). **Table S5.** MaAsLin2 results of the bacterial taxa that are altered by treatment response, controlling for infection status and village as fixed effects. **Table S6.** MaAsLin2 results of the bacterial taxa that are associated with treatment response, associated with helminth status or not, correcting for village as a covariate. **Table S7.** Relative impact of the deworming on gut microbiome dissimilarity of different group of Orang Asli samples based on their gut microbiome data in pre, 21-day, and 42-day post- anthelmintic treatment (ADONIS, ANOSIM, and Betadisper; permutation = 999). **Table S8.** Spearman correlation analysis on the growth rate (GRiD score) of the bacterial species with *Trichuris* burden among the pre-treatment samples. **Table S9.** Spearman correlation analysis on the growth rate (GRiD score) of the bacterial species with *Trichuris* burden among the Responders. **Table S10.** MaAsLin2 results of the bacterial replication (based on GRiD score) that are associated with *Trichuris* infection while controlling for treatment group and villages.

**Additional file 2: Figure S1.** A geographic map showing the locations of each village and the Kuala Lumpur city in Peninsular Malaysia (stars and numbers) together with a table with other information including states, tribes and subtribes. **Figure S2.** A flow diagram of the total number of subjects (Orang Asli and urban citizens from Kuala Lumpur) involved in both the pre-anthelmintic and post-anthelmintic of this study. **Figure S3.** A flow diagram summarizing the bioinformatic analysis from raw reads, 1) Quality filtering, remove human reads and adapter (KneadData), taxonomic classification (Kraken2 and Bracken2), 3) *K*-mer based approach (Sourmash), 4) Estimation of bacterial growth rate (GRiD) to downstream analysis (A–C) such as beta diversity, alpha diversity, effect size estimation and differential abundance, and 5) Functional genes and pathways analysis using HUMAnN v3.0 and its UniRef 50, Pfam, and MetaCyc pathway databases. **Figure S4.** Difference in the composition of core microbiota between Orang Asli cohort and KL cohort in different taxonomic rank, which include: A Class, B Order, C Family, D Genus, and E Species. **Figure S5.** Difference in the composition of core microbiota between different geographical location in different taxonomic rank, which include A Family, B Genus, and C Species. **Figure S6.** A Bar plot of the top 20 species that best predict the difference of the core gut microbiota between Orang Asli (OA) cohort and Kuala Lumpur (KL) cohort using a Random Forest classification model B and C box plots displaying the selected core microbial species that have high variation between Orang Asli (OA) cohort and Kuala Lumpur (KL) cohort based on the Random Forest analysis. The relative abundances of core microbial species between Orang Asli cohort and KL cohort were tested using Wilcoxon rank sum test. B Species with significant higher abundance in Orang Asli cohort than KL cohort, which include (from left to right): HRGM Genome 3145, *Gemmiger sp900539695*, and *Blautia A sp00043661,* respectively. C Species with significant higher abundance in KL cohort than the Orang Asli cohort, which include (from left to right): *Megamonas funiformis*, *Phocaeicola plebeius A*, and *Bacteroides stercoris,* respectively. **Figure S7.** Effects of geographical location on core gut microbiota and the percentage of unmapped reads in the microbiome. A The core gut microbial species showing the largest variation (cut-off 6.0 for the coefficient of variation) between Orang Asli and Kuala Lumpur cohort in Malaysia across 650 samples. Box plots illustrate the percentage of mapped reads in B RefSeq (i.e., Bacteria, protozoa, fungi, viral, archaea) database, and C Unified Human Gastrointestinal Genome (UHGG) database in different geographical locations. Pairwise comparison between each village and the KL cohort was tested using Wilcoxon rank sum test whereas the comparison for all groups was tested using Kruskal-Wallis. **Figure S8.** Beta diversity of 650 samples [Orang Asli (OA) and Kuala Lumpur (KL) cohort]. Comparison of pairwise beta diversity of all villages to KL cohort, assessed by Jaccard distance based on distance of A nucleotide *k*-mer sketches (*k* = 51), B *k*-mer sketches (*k* = 31), and C species level. Pairwise comparison between each village against the KL cohort was tested using Wilcoxon rank sum test. Principal Coordinates Analysis (PCoA) of Jaccard distance based on D genus, E *k*-mer sketches = 21, F *k*-mer sketches = 31, and G *k*-mer sketches = 51 in OA and KL cohort. The individuals from different geographical locations were denoted by different colors. **Figure S9.** Epidemiology data of the Orang Asli (OA) and Kuala Lumpur (KL) cohort. A Distribution of the age group from OA and KL cohort, the OA and KL cohort were denoted by purple and pink color, respectively. B Distribution of the gender from OA and KL cohort, the female and male cohorts were denoted by blue and yellow color, respectively. C The prevalence of different types of helminthiases, which include *Trichuris* infection, *Ascaris* infection and hookworm infection, the heavy, moderate and light infection were detonated by different purple color intensity. **Figure S10.** Beta diversity comparing the gut microbiome between intestinal helminth-infected and uninfected Orang Asli cohorts at species level. The results were visualized using Non-metric multidimensional scaling (NMDS) plot of A Bray-Curtis (ADONIS: *p* = 0.001, *R*^2^ = 0.035; ANOSIM: *p* = 0.001, *R* = 0.149) and B Jaccard distance (ADONIS: *p* = 0.001, *R*^2^ = 0.024; ANOSIM: *p* = 0.001, *R* = 0.948) and C Principal Coordinates Analysis (PCoA) of Jaccard distance (ADONIS: *p* = 0.001, *R*^2^ = 0.024; ANOSIM: *p* = 0.001, *R* = 0.948). The individuals infected and uninfected with intestinal helminths denoted by blue and red color, respectively. **Figure S11.** Box plots showing alpha diversity of gut microbiome profile at species level using A Shannon diversity and B Simpson diversity index on individuals infected and uninfected with intestinal helminths; C Shannon diversity and D Simpson diversity index on different numbers of intestinal helminth infection; E Shannon diversity and F Simpson diversity index on individuals infected and uninfected with *Trichuris* sp. infection; and G Shannon diversity and H Simpson diversity index on different villages. The statistical difference between two groups was tested using the Wilcoxon rank sum test whereas more than two groups was tested using Kruskal-Wallis. **Figure S12.** Box plots showing alpha diversity (i.e., Richness, Shannon and Simpson diversity index) at species level of gut microbiome profile on Orang Asli who are infected and uninfected with intestinal helminths from A Rasau, B Legong, C Judah, D Sepat, and E Bangkong. The comparison of the alpha-diversity index between helminth-infected and noninfected samples is tested using the Wilcoxon rank sum test. **Figure S13.** Bubble plot shows bacterial species that are differentially abundant between *Trichuris* infected and uninfected groups in all samples, as well as specific villages based on the output of the A Multivariate Association with Linear Models (MaAsLin2) and B Analysis of Compositions of Microbiomes with Bias Correction (ANCOM-BC). The size of the bubble is negatively proportional to the *p*-value. The larger the bubble size displaying the lower *p*-value. **Figure S14.** Boxplot shows the differences in the abundance of A *Lactobacillus gasseri* and B *Lactobacillus crispatus* in helminthic infections (left) and different villages (right). The statistical difference between two groups was tested using the Wilcoxon rank sum test whereas more than two groups (Village) was tested using Kruskal-Wallis. **Figure S15.** Bar chart shows the changes in the prevalence of different types of helminthic infections in (left), and the prevalence of the number of helminthic infections (right) among the pre-anthelmintic, 21-day, and 42-day post-anthelmintic. Boxplot showing alpha diversity (i.e., Richness, Shannon and Simpson diversity index) of gut microbiome profile at species level on the B Responder, and C Uninfected. The comparison of the alpha-diversity index between helminth-infected and noninfected samples is tested using the Wilcoxon signed-rank test. **Figure S16.** Beta diversity comparing the gut microbiome at species level between pre-anthelmintic (blue) and post-anthelmintic (red) among the Responders, visualized using Non-metric multidimensional scaling (NMDS) plot of A Jaccard distance (ADONIS: *p* = 0.001, *R*^2^ = 0.014; ANOSIM: *p* = 0.001, *R* = 0.072) and B Bray-Curtis (ADONIS: *p* = 0.001, *R*^2^ = 0.020; ANOSIM: *p* = 0.001, *R* = 0.072). Principal Coordinates Analysis (PCoA) coordinate plot showing the beta-dispersion of C Jaccard distance and D Bray-Curtis distance based on gut microbiota profile of the Responders, with pre-anthelmintic (red) and post-anthelmintic (black). E PCoA plot showing the beta diversity of Bray-Curtis distances based on gut microbiota profile of Responders. **Figure S17.** Beta diversity comparing the gut microbiome at species level between pre-anthelminthic (blue) and post-anthelmintic (red) among the Uninfected, visualized using Non-metric multidimensional scaling (NMDS) plot of A Jaccard distance (ADONIS: *p* = 0.005, *R*^2^ = 0.012; ANOSIM: *p* = 0.001, *R* = 0.069) and B Bray-Curtis (ADONIS: *p* = 0.003, *R*^2^ = 0.015; ANOSIM: *p* = 0.001, *R* = 0.069). Principal Coordinates Analysis (PCoA) coordinate plot showing the beta-dispersion of C Jaccard distance and D Bray-Curtis distance based on gut microbiota profile of the Uninfected, with pre-anthelmintic (red) and post-anthelmintic (black). E PCoA plot showing the beta diversity of Bray-Curtis distances based on gut microbiota profile of the Uninfected. **Figure S18.** A Bubble plots of the top 10 bacterial species that differentially abundant between pre-anthelmintic and post-anthelmintic in Responders as well as the Uninfected based on the output of the Microbiome Multivariable Association with Linear Models 2 (MaAsLin2). The size of the bubble is negatively proportional to the *p*-value. The larger the bubble size displaying the lower *p*-value and, Line plots showing changes to three of the top differentially abundant bacterial species between pre and post treatment samples from B Responders and C Uninfected individuals, with *p*-values determined by the Wilcoxon signed-rank test. **Figure S19.** A Heatmap shows the bacterial species that are associated with treatment response while including infection status and village locations as covariates from MaAsLin2 analysis. Blue for positive association and red for negative association. B Boxplots show the differences in the abundance of CAG245 sp900552135 between different village (top), Helminth infections status (middle) and different response group (Uninfected or Responders) (bottom). The statistical difference between two groups was tested using the Wilcoxon rank sum test (cross-sectional) or Wilcoxon signed-rank test (longitudinal) whereas more than two groups (Village) was tested using Kruskal-Wallis test. C Heatmap shows the bacterial species that are associated with drug response while correcting helminth status from MaAsLin2 analysis. Blue for positive association and red for negative association. **Figure S20.** A Alpha diversity at species level, which visualized using the line plot of the Richness, Shannon and Simpson diversity indices of the Orang Asli (OA) in pre-anthelmintic, 21-day and 42-day post-anthelmintic groups, for Responders (green) and Uninfected (red) individuals. Alpha-diversity index of three timepoints were compared using the Friedman test whereas two timepoints was compared using the Wilcoxon signed-rank test. There are no statistical differences between groups. B Principal coordinates Analysis (PCoA), C Non-metric multidimensional scaling (NMDS), and D Beta-dispersion of Jaccard distance based on gut microbiota profile at species level of the pre-anthelmintic (purple), 21-day (green), and 42-day post-anthelmintic (gold) from the Responders (ADONIS: *p* = 0.001, *R*^2^ = 0.017; ANOSIM: *p* = 0.001, *R* = 0.053) (left) and Uninfected (ADONIS: *p* = 0.219, *R*^2^ = 0.011; ANOSIM: *p* = 0.001, *R* = 0.052) (right). **Figure S21.** Growth Rate Index (GRiD) analysis of the gut bacteria in the Orang Asli (OA) cohort. Correlation matrix of the top 20 gut microbial species that correlate with the infection intensity of *Trichuris trichiura* in A pre-treatment samples and B among Responders. **Figure S22.** A Heatmap showing the replication the gut microbial species that are associated with intestinal helminth infection among the Responders. The first vertical side bar encodes the intestinal helminth infection while the second side bar indicates the infection intensity of the *Trichuris*. B Box plots showing the two bacteria (i.e., uncultured *Oscilibacter* sp. [left] and *Phocaeicola vulgatus* [right]) that are significantly negatively correlated with the infection intensity of the *Trichuris* in Responders. The statistical difference between two groups was tested using the Wilcoxon rank sum test. C GRiD score correlation between bacterial species with the infection intensity of *Trichuris* among the Responders. The bar chart shows the Spearman’s rank correlation coefficient. D Heatmap showing the replication of the gut microbial species that are associated with albendazole treatment among the uninfected. Samples are shown in row by different timepoints (pre-anthelmintic and post-anthelmintic) whereas the rank of the GRiD score of each bacterium is shown in column. **Figure S23.** Barplot shows all the pathways that most significantly different between Orang Asli and Urban citizen from Kuala Lumpur (by controlling age and gender) (top), Village (with helminth as covariates) (middle), and Albendazole response (which include helminth and village as covariates) (bottom) based on the output of the Microbiome Multivariable Association with Linear Models 2 (MaAsLin2). The length of the bar corresponds to the value of the significant association (can be either positive or negative). **Figure S2.** Barplot shows all the gene families that most significantly different between Orang Asli and Urban cohort from Kuala Lumpur (by controlling age and gender), Village (with helminth as covariate) (middle), C Albendazole response (which include helminth and village as covariates) (bottom) based on the output of the Microbiome Multivariable Association with Linear Models 2 (MaAsLin2). The length of the bar corresponds to the value of the significant association (can be either positive or negatively associated). **Figure S25.** Box plot shows the abundance of the top pathways or gene families based on the output of the Microbiome Multivariable Association with Linear Models 2 (MaAsLin2), which include abundance of A Superpathway of L−tryptophan biosynthesis between Orang Asli and Urban cohort from Kuala Lumpur, B Peptidoglycan biosynthesis II (staphylococci) between different villages, C L-glutamate degradation V between pre- and post-albendazole treatment groups, and D Phosphoenolpyruvate carboxylase between pre- and post-albendazole treatment groups. The statistical difference between two groups was tested using the Wilcoxon rank sum test (cross-sectional) or Wilcoxon signed-rank test (longitudinal) whereas more than two groups (Village) was tested using Kruskal-Wallis test. **Figure S26.** Summary of the methodology from field work, sample collection, shotgun metagenomic sequencing, and data analysis. **Figure S27.** Flow diagram of the filtering steps before downstream analysis (beta diversity, alpha diversity, and differential abundance). **Figure S28.** Methodology for the evaluation of microbial growth rate in relation to helminth infection status in both cross-sectional and longitudinal phase using GRiD analysis.

## Data Availability

Raw data of gut metagenome has been deposited on the NCBI Sequence Read Archive with the BioProject No. PRJNA797994 and BioSample accession No. SAMN25042866-25043515. In addition, the data have been uploaded to MicrobiomeDB (microbiomedb.org) under named “Malaysia helminth study.”
