# Supplementary figures and images for: Gut microbiome of helminth-infected indigenous Malaysians is context dependent
Source: Microbiome. 2022 Dec 7;10:214. doi: 10.1186/s40168-022-01385-x (PMC9727879; doi:10.1186/s40168-022-01385-x)

Fig. S10

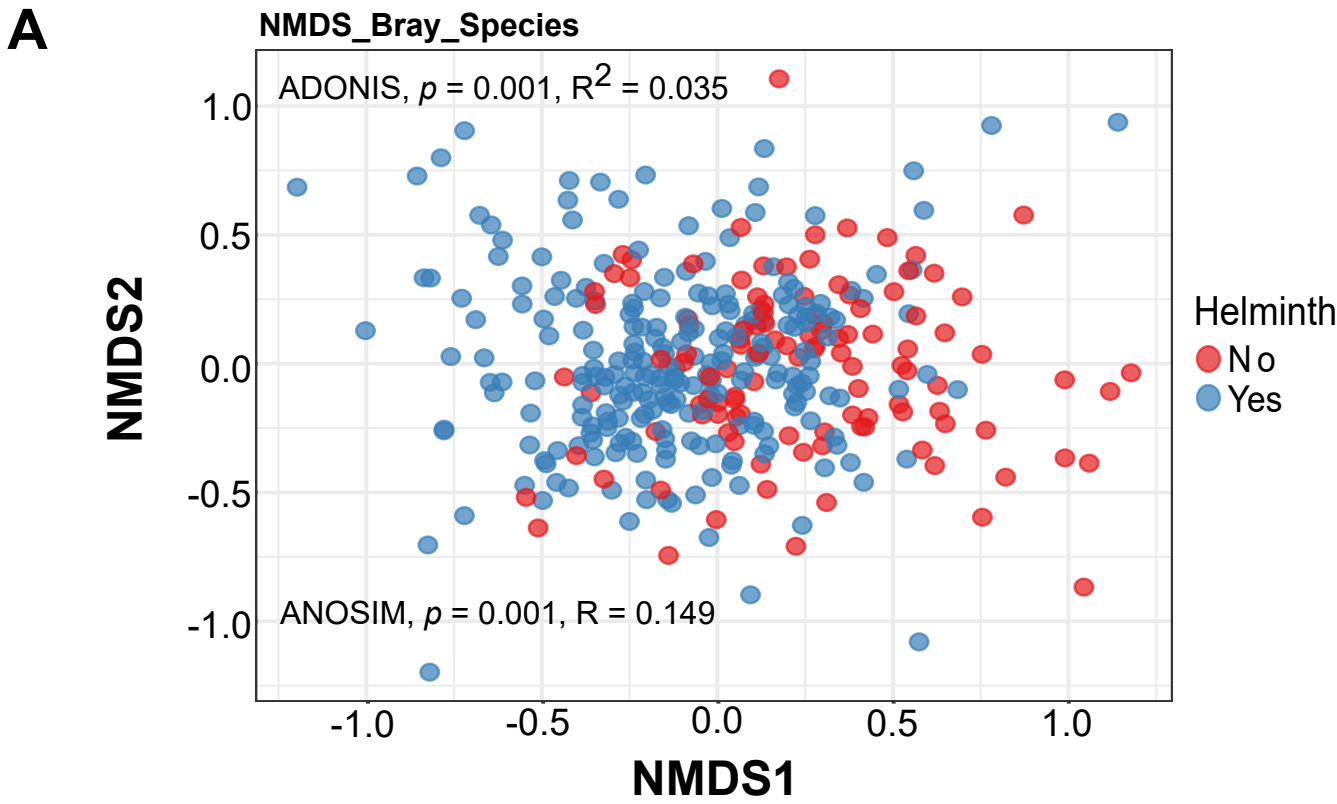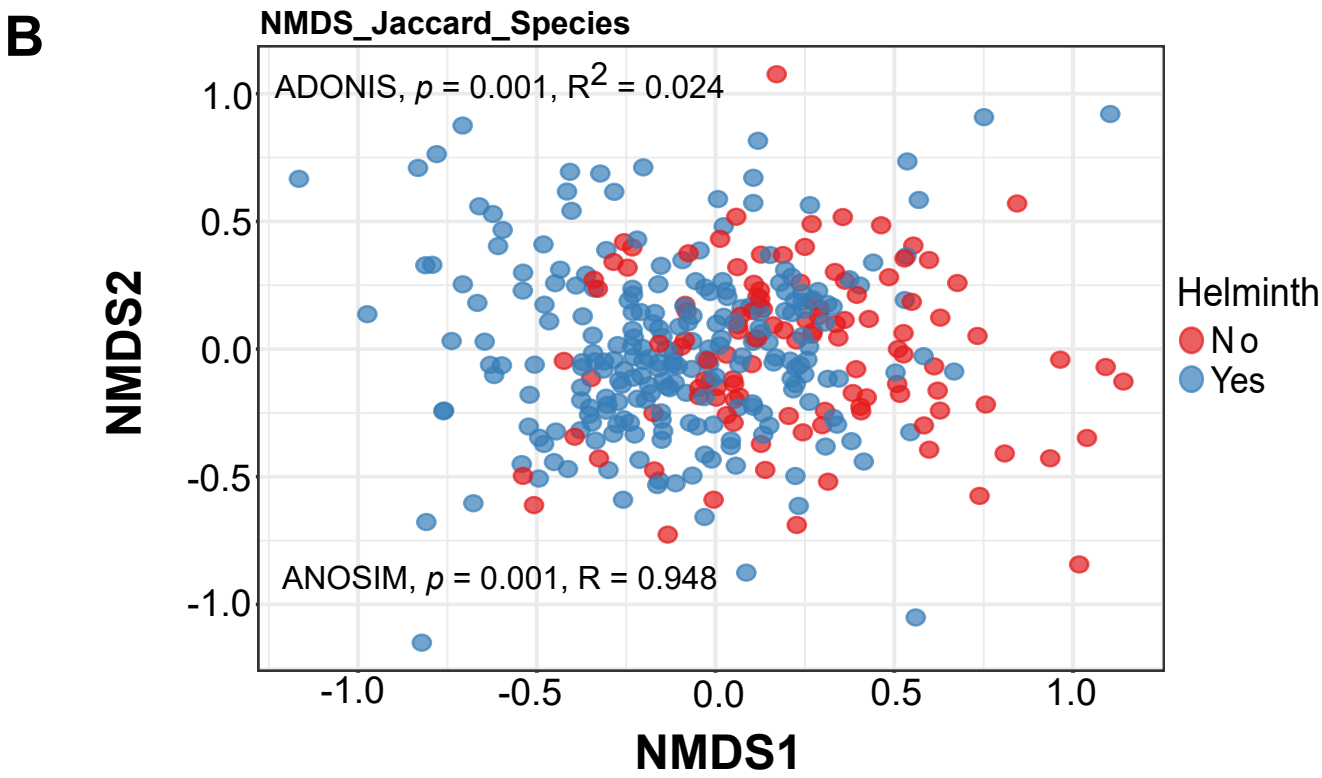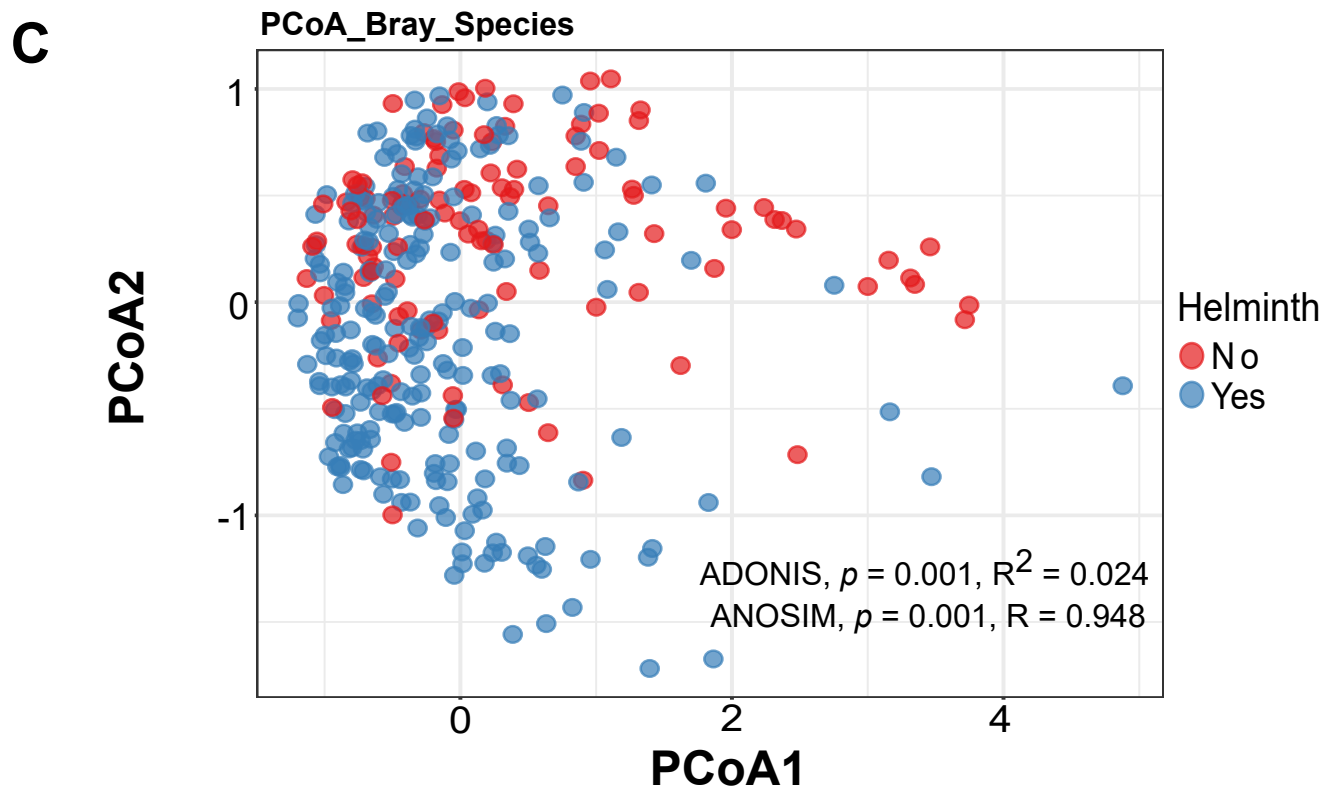

Supplement: Supplementary file 3 — Additional file 2: Figure S1. A geographic map showing the locations of each village and the Kuala Lumpur city in Peninsular Malaysia (stars and numbers) together with a table with other information including states, tribes and subtribes. Figure S2. A flow diagram of the total number of subjects (Orang Asli and urban citizens from Kuala Lumpur) involved in both the pre-anthelmintic and post-anthelmintic of this study. Figure S3. A flow diagram summarizing the bioinformatic analysis from raw reads, 1) Quality filtering, remove human reads and adapter (KneadData), taxonomic classification (Kraken2 and Bracken2), 3) K-mer based approach (Sourmash), 4) Estimation of bacterial growth rate (GRiD) to downstream analysis (A–C) such as beta diversity, alpha diversity, effect size estimation and differential abundance, and 5) Functional genes and pathways analysis using HUMAnN v3.0 and its UniRef 50, Pfam, and MetaCyc pathway databases. Figure S4. Difference in the composition of core microbiota between Orang Asli cohort and KL cohort in different taxonomic rank, which include: A Class, B Order, C Family, D Genus, and E Species. Figure S5. Difference in the composition of core microbiota between different geographical location in different taxonomic rank, which include A Family, B Genus, and C Species. Figure S6. A Bar plot of the top 20 species that best predict the difference of the core gut microbiota between Orang Asli (OA) cohort and Kuala Lumpur (KL) cohort using a Random Forest classification model B and C box plots displaying the selected core microbial species that have high variation between Orang Asli (OA) cohort and Kuala Lumpur (KL) cohort based on the Random Forest analysis. The relative abundances of core microbial species between Orang Asli cohort and KL cohort were tested using Wilcoxon rank sum test. B Species with significant higher abundance in Orang Asli cohort than KL cohort, which include (from left to right): HRGM Genome 3145, Gemmiger sp900539695, and [file 40168_2022_1385_MOESM2_ESM.zip › Supplementary_Figure_S10.pdf]

**Fig. S11**

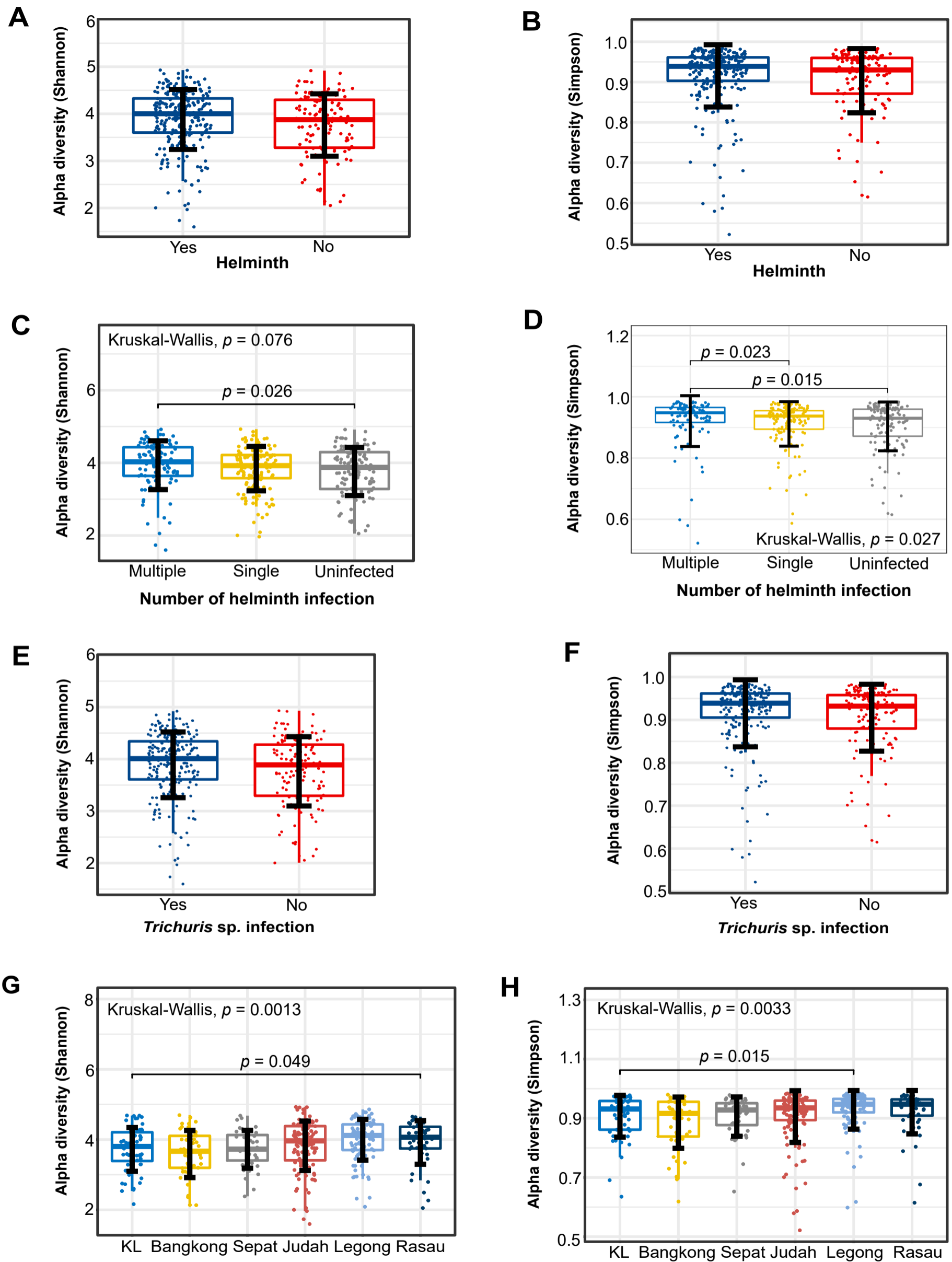

Supplement: Supplementary file 3 — Additional file 2: Figure S1. A geographic map showing the locations of each village and the Kuala Lumpur city in Peninsular Malaysia (stars and numbers) together with a table with other information including states, tribes and subtribes. Figure S2. A flow diagram of the total number of subjects (Orang Asli and urban citizens from Kuala Lumpur) involved in both the pre-anthelmintic and post-anthelmintic of this study. Figure S3. A flow diagram summarizing the bioinformatic analysis from raw reads, 1) Quality filtering, remove human reads and adapter (KneadData), taxonomic classification (Kraken2 and Bracken2), 3) K-mer based approach (Sourmash), 4) Estimation of bacterial growth rate (GRiD) to downstream analysis (A–C) such as beta diversity, alpha diversity, effect size estimation and differential abundance, and 5) Functional genes and pathways analysis using HUMAnN v3.0 and its UniRef 50, Pfam, and MetaCyc pathway databases. Figure S4. Difference in the composition of core microbiota between Orang Asli cohort and KL cohort in different taxonomic rank, which include: A Class, B Order, C Family, D Genus, and E Species. Figure S5. Difference in the composition of core microbiota between different geographical location in different taxonomic rank, which include A Family, B Genus, and C Species. Figure S6. A Bar plot of the top 20 species that best predict the difference of the core gut microbiota between Orang Asli (OA) cohort and Kuala Lumpur (KL) cohort using a Random Forest classification model B and C box plots displaying the selected core microbial species that have high variation between Orang Asli (OA) cohort and Kuala Lumpur (KL) cohort based on the Random Forest analysis. The relative abundances of core microbial species between Orang Asli cohort and KL cohort were tested using Wilcoxon rank sum test. B Species with significant higher abundance in Orang Asli cohort than KL cohort, which include (from left to right): HRGM Genome 3145, Gemmiger sp900539695, and [file 40168_2022_1385_MOESM2_ESM.zip › Supplementary_Figure_S11.pdf]

Fig. S12

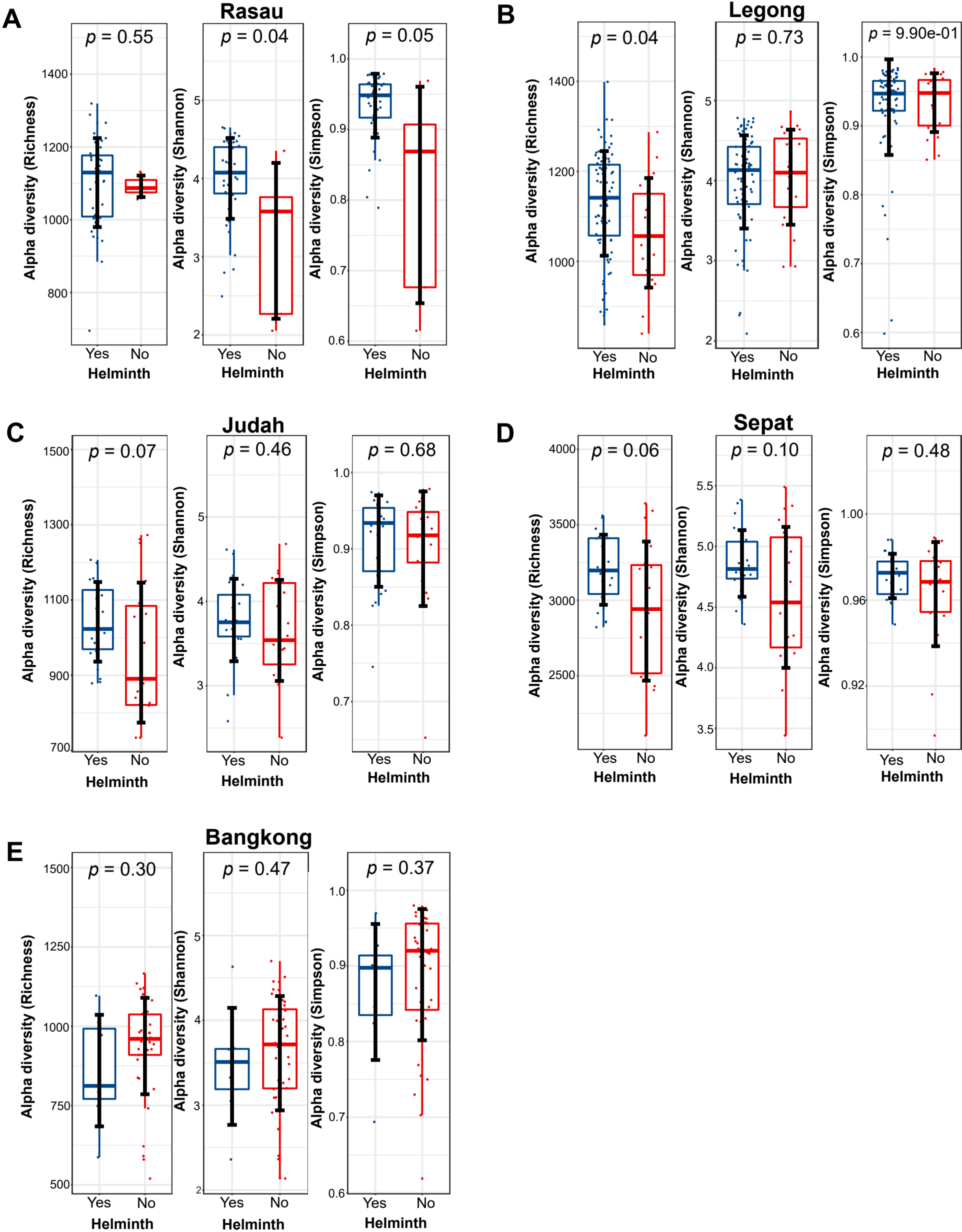

Supplement: Supplementary file 3 — Additional file 2: Figure S1. A geographic map showing the locations of each village and the Kuala Lumpur city in Peninsular Malaysia (stars and numbers) together with a table with other information including states, tribes and subtribes. Figure S2. A flow diagram of the total number of subjects (Orang Asli and urban citizens from Kuala Lumpur) involved in both the pre-anthelmintic and post-anthelmintic of this study. Figure S3. A flow diagram summarizing the bioinformatic analysis from raw reads, 1) Quality filtering, remove human reads and adapter (KneadData), taxonomic classification (Kraken2 and Bracken2), 3) K-mer based approach (Sourmash), 4) Estimation of bacterial growth rate (GRiD) to downstream analysis (A–C) such as beta diversity, alpha diversity, effect size estimation and differential abundance, and 5) Functional genes and pathways analysis using HUMAnN v3.0 and its UniRef 50, Pfam, and MetaCyc pathway databases. Figure S4. Difference in the composition of core microbiota between Orang Asli cohort and KL cohort in different taxonomic rank, which include: A Class, B Order, C Family, D Genus, and E Species. Figure S5. Difference in the composition of core microbiota between different geographical location in different taxonomic rank, which include A Family, B Genus, and C Species. Figure S6. A Bar plot of the top 20 species that best predict the difference of the core gut microbiota between Orang Asli (OA) cohort and Kuala Lumpur (KL) cohort using a Random Forest classification model B and C box plots displaying the selected core microbial species that have high variation between Orang Asli (OA) cohort and Kuala Lumpur (KL) cohort based on the Random Forest analysis. The relative abundances of core microbial species between Orang Asli cohort and KL cohort were tested using Wilcoxon rank sum test. B Species with significant higher abundance in Orang Asli cohort than KL cohort, which include (from left to right): HRGM Genome 3145, Gemmiger sp900539695, and [file 40168_2022_1385_MOESM2_ESM.zip › Supplementary_Figure_S12.pdf]

Fig. S13

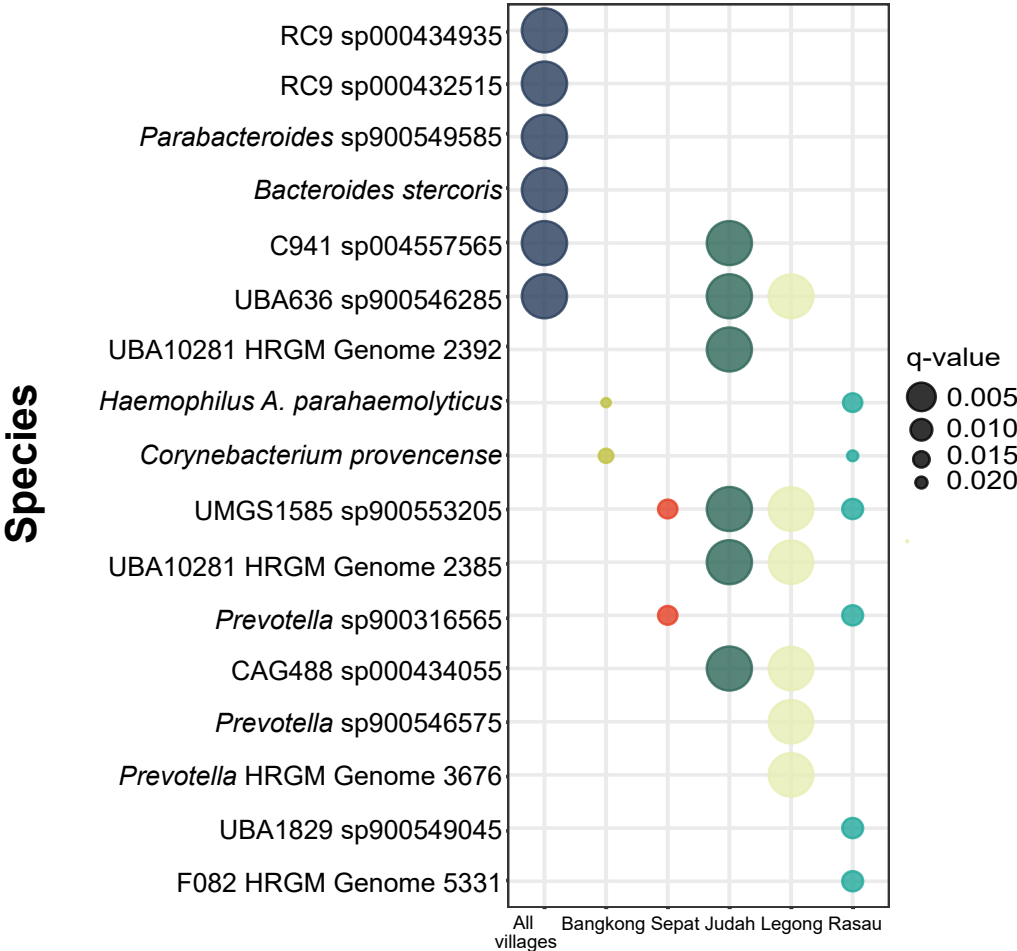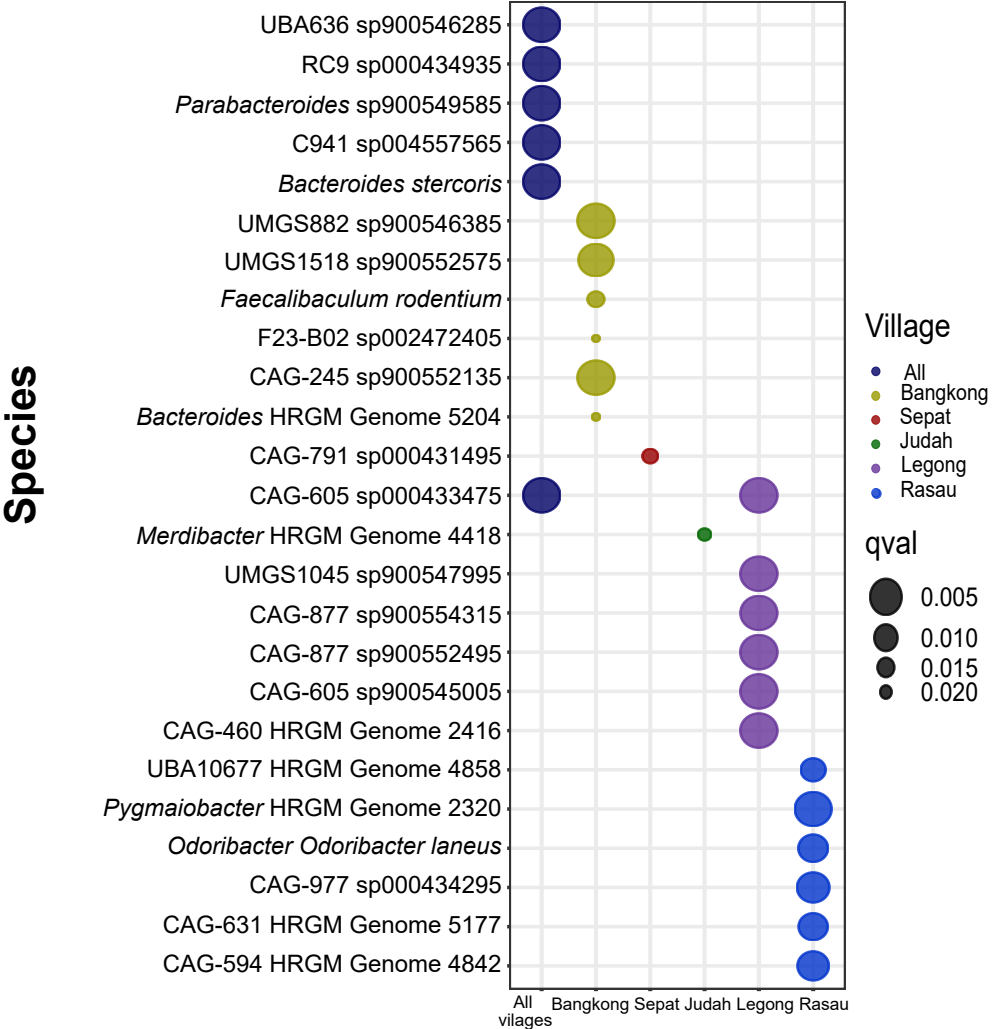

Supplement: Supplementary file 3 — Additional file 2: Figure S1. A geographic map showing the locations of each village and the Kuala Lumpur city in Peninsular Malaysia (stars and numbers) together with a table with other information including states, tribes and subtribes. Figure S2. A flow diagram of the total number of subjects (Orang Asli and urban citizens from Kuala Lumpur) involved in both the pre-anthelmintic and post-anthelmintic of this study. Figure S3. A flow diagram summarizing the bioinformatic analysis from raw reads, 1) Quality filtering, remove human reads and adapter (KneadData), taxonomic classification (Kraken2 and Bracken2), 3) K-mer based approach (Sourmash), 4) Estimation of bacterial growth rate (GRiD) to downstream analysis (A–C) such as beta diversity, alpha diversity, effect size estimation and differential abundance, and 5) Functional genes and pathways analysis using HUMAnN v3.0 and its UniRef 50, Pfam, and MetaCyc pathway databases. Figure S4. Difference in the composition of core microbiota between Orang Asli cohort and KL cohort in different taxonomic rank, which include: A Class, B Order, C Family, D Genus, and E Species. Figure S5. Difference in the composition of core microbiota between different geographical location in different taxonomic rank, which include A Family, B Genus, and C Species. Figure S6. A Bar plot of the top 20 species that best predict the difference of the core gut microbiota between Orang Asli (OA) cohort and Kuala Lumpur (KL) cohort using a Random Forest classification model B and C box plots displaying the selected core microbial species that have high variation between Orang Asli (OA) cohort and Kuala Lumpur (KL) cohort based on the Random Forest analysis. The relative abundances of core microbial species between Orang Asli cohort and KL cohort were tested using Wilcoxon rank sum test. B Species with significant higher abundance in Orang Asli cohort than KL cohort, which include (from left to right): HRGM Genome 3145, Gemmiger sp900539695, and [file 40168_2022_1385_MOESM2_ESM.zip › Supplementary_Figure_S13.pdf]

Fig. S14

**A**

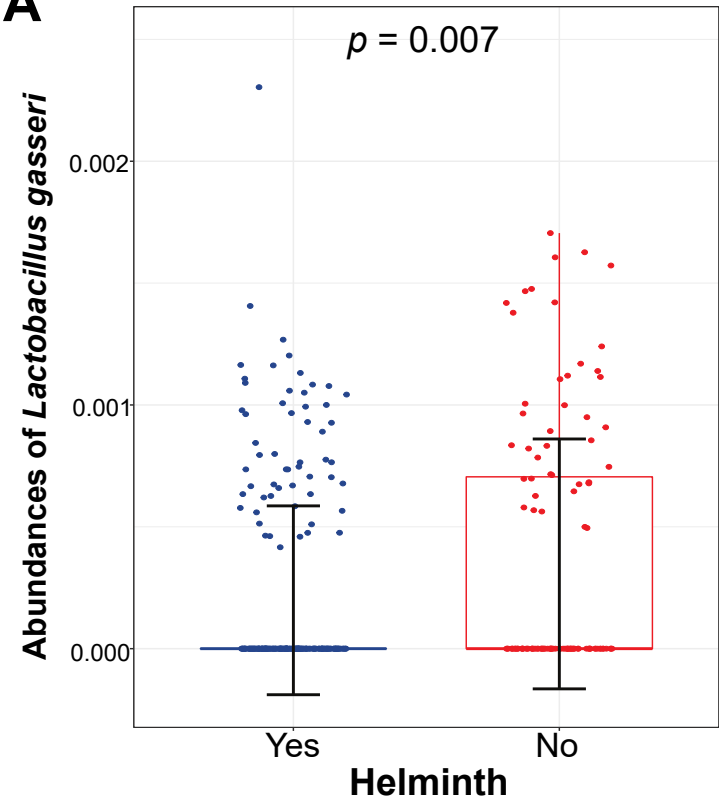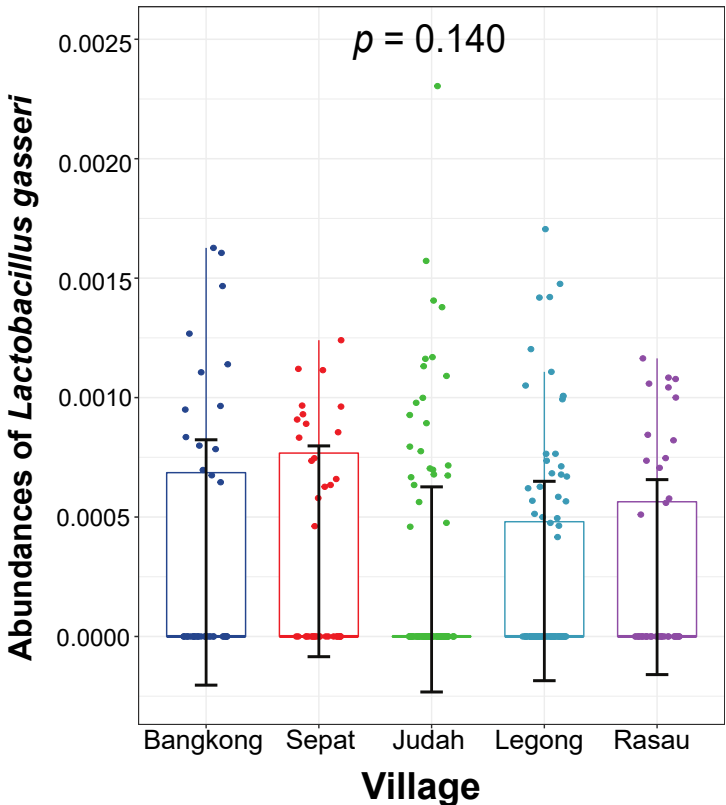

**B**

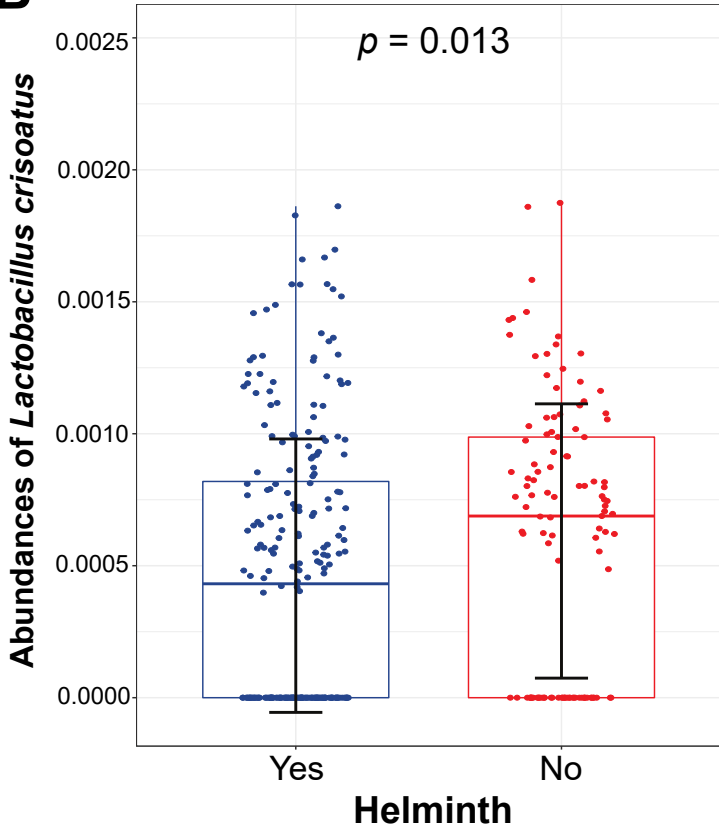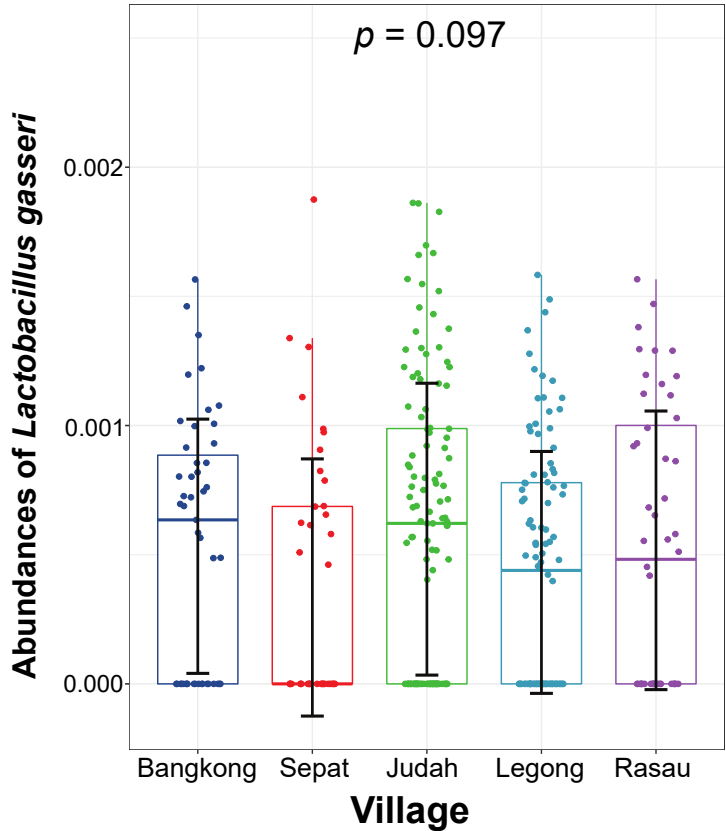

Supplement: Supplementary file 3 — Additional file 2: Figure S1. A geographic map showing the locations of each village and the Kuala Lumpur city in Peninsular Malaysia (stars and numbers) together with a table with other information including states, tribes and subtribes. Figure S2. A flow diagram of the total number of subjects (Orang Asli and urban citizens from Kuala Lumpur) involved in both the pre-anthelmintic and post-anthelmintic of this study. Figure S3. A flow diagram summarizing the bioinformatic analysis from raw reads, 1) Quality filtering, remove human reads and adapter (KneadData), taxonomic classification (Kraken2 and Bracken2), 3) K-mer based approach (Sourmash), 4) Estimation of bacterial growth rate (GRiD) to downstream analysis (A–C) such as beta diversity, alpha diversity, effect size estimation and differential abundance, and 5) Functional genes and pathways analysis using HUMAnN v3.0 and its UniRef 50, Pfam, and MetaCyc pathway databases. Figure S4. Difference in the composition of core microbiota between Orang Asli cohort and KL cohort in different taxonomic rank, which include: A Class, B Order, C Family, D Genus, and E Species. Figure S5. Difference in the composition of core microbiota between different geographical location in different taxonomic rank, which include A Family, B Genus, and C Species. Figure S6. A Bar plot of the top 20 species that best predict the difference of the core gut microbiota between Orang Asli (OA) cohort and Kuala Lumpur (KL) cohort using a Random Forest classification model B and C box plots displaying the selected core microbial species that have high variation between Orang Asli (OA) cohort and Kuala Lumpur (KL) cohort based on the Random Forest analysis. The relative abundances of core microbial species between Orang Asli cohort and KL cohort were tested using Wilcoxon rank sum test. B Species with significant higher abundance in Orang Asli cohort than KL cohort, which include (from left to right): HRGM Genome 3145, Gemmiger sp900539695, and [file 40168_2022_1385_MOESM2_ESM.zip › Supplementary_Figure_S14.pdf]

Fig. S15

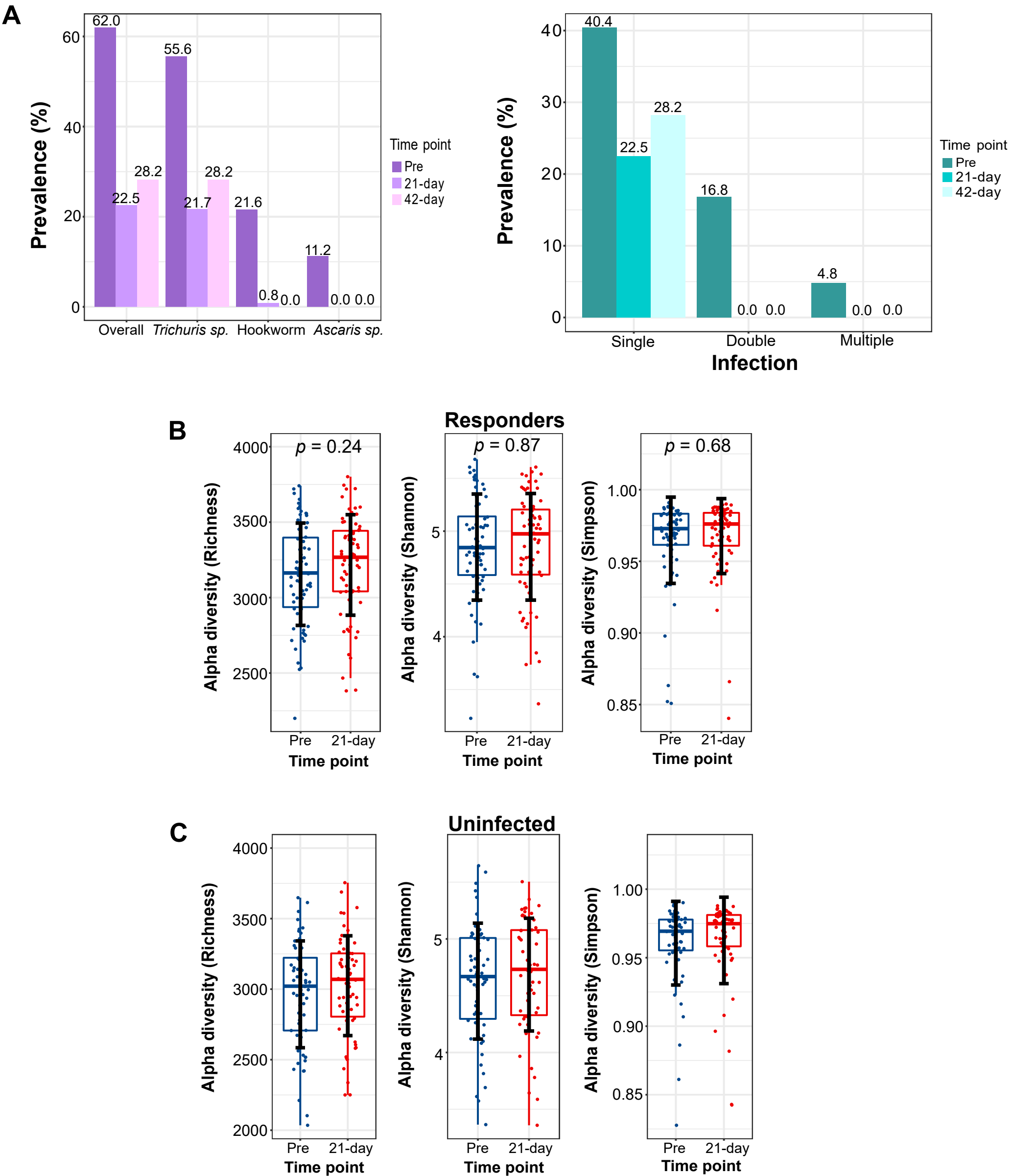

Supplement: Supplementary file 3 — Additional file 2: Figure S1. A geographic map showing the locations of each village and the Kuala Lumpur city in Peninsular Malaysia (stars and numbers) together with a table with other information including states, tribes and subtribes. Figure S2. A flow diagram of the total number of subjects (Orang Asli and urban citizens from Kuala Lumpur) involved in both the pre-anthelmintic and post-anthelmintic of this study. Figure S3. A flow diagram summarizing the bioinformatic analysis from raw reads, 1) Quality filtering, remove human reads and adapter (KneadData), taxonomic classification (Kraken2 and Bracken2), 3) K-mer based approach (Sourmash), 4) Estimation of bacterial growth rate (GRiD) to downstream analysis (A–C) such as beta diversity, alpha diversity, effect size estimation and differential abundance, and 5) Functional genes and pathways analysis using HUMAnN v3.0 and its UniRef 50, Pfam, and MetaCyc pathway databases. Figure S4. Difference in the composition of core microbiota between Orang Asli cohort and KL cohort in different taxonomic rank, which include: A Class, B Order, C Family, D Genus, and E Species. Figure S5. Difference in the composition of core microbiota between different geographical location in different taxonomic rank, which include A Family, B Genus, and C Species. Figure S6. A Bar plot of the top 20 species that best predict the difference of the core gut microbiota between Orang Asli (OA) cohort and Kuala Lumpur (KL) cohort using a Random Forest classification model B and C box plots displaying the selected core microbial species that have high variation between Orang Asli (OA) cohort and Kuala Lumpur (KL) cohort based on the Random Forest analysis. The relative abundances of core microbial species between Orang Asli cohort and KL cohort were tested using Wilcoxon rank sum test. B Species with significant higher abundance in Orang Asli cohort than KL cohort, which include (from left to right): HRGM Genome 3145, Gemmiger sp900539695, and [file 40168_2022_1385_MOESM2_ESM.zip › Supplementary_Figure_S15.pdf]

Fig. S16

Responders

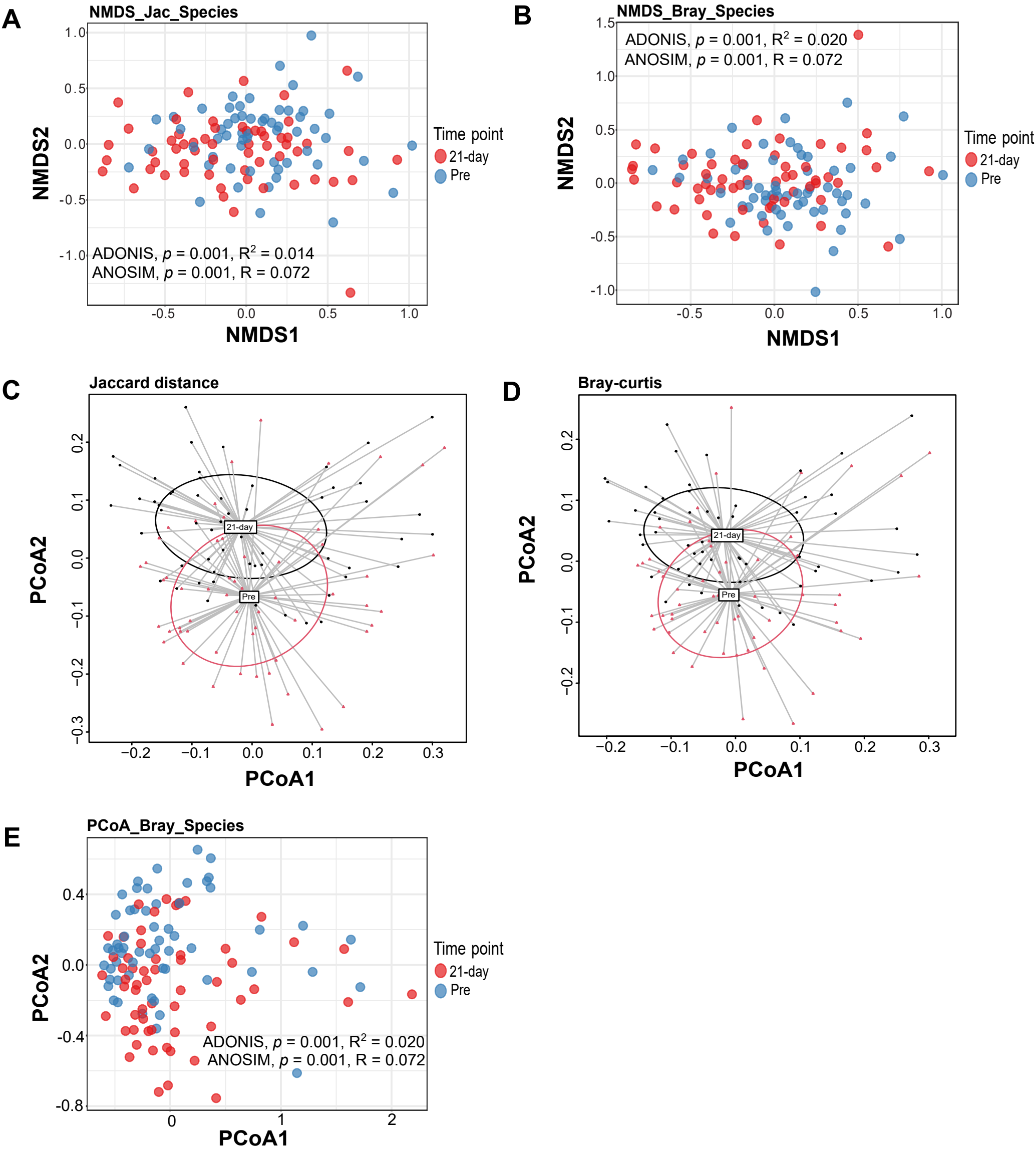

Supplement: Supplementary file 3 — Additional file 2: Figure S1. A geographic map showing the locations of each village and the Kuala Lumpur city in Peninsular Malaysia (stars and numbers) together with a table with other information including states, tribes and subtribes. Figure S2. A flow diagram of the total number of subjects (Orang Asli and urban citizens from Kuala Lumpur) involved in both the pre-anthelmintic and post-anthelmintic of this study. Figure S3. A flow diagram summarizing the bioinformatic analysis from raw reads, 1) Quality filtering, remove human reads and adapter (KneadData), taxonomic classification (Kraken2 and Bracken2), 3) K-mer based approach (Sourmash), 4) Estimation of bacterial growth rate (GRiD) to downstream analysis (A–C) such as beta diversity, alpha diversity, effect size estimation and differential abundance, and 5) Functional genes and pathways analysis using HUMAnN v3.0 and its UniRef 50, Pfam, and MetaCyc pathway databases. Figure S4. Difference in the composition of core microbiota between Orang Asli cohort and KL cohort in different taxonomic rank, which include: A Class, B Order, C Family, D Genus, and E Species. Figure S5. Difference in the composition of core microbiota between different geographical location in different taxonomic rank, which include A Family, B Genus, and C Species. Figure S6. A Bar plot of the top 20 species that best predict the difference of the core gut microbiota between Orang Asli (OA) cohort and Kuala Lumpur (KL) cohort using a Random Forest classification model B and C box plots displaying the selected core microbial species that have high variation between Orang Asli (OA) cohort and Kuala Lumpur (KL) cohort based on the Random Forest analysis. The relative abundances of core microbial species between Orang Asli cohort and KL cohort were tested using Wilcoxon rank sum test. B Species with significant higher abundance in Orang Asli cohort than KL cohort, which include (from left to right): HRGM Genome 3145, Gemmiger sp900539695, and [file 40168_2022_1385_MOESM2_ESM.zip › Supplementary_Figure_S16.pdf]

Fig. S17

Uninfected

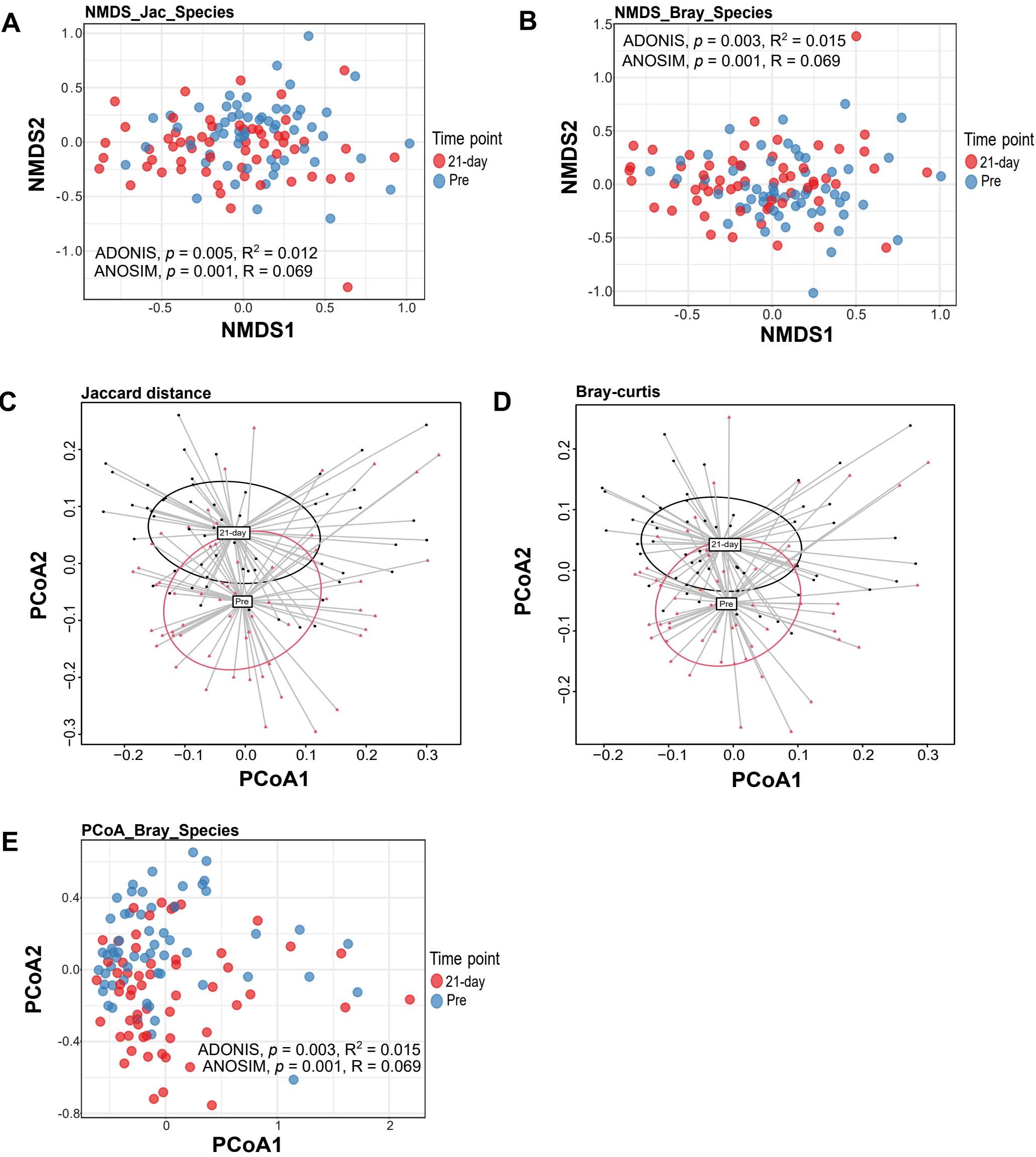

Supplement: Supplementary file 3 — Additional file 2: Figure S1. A geographic map showing the locations of each village and the Kuala Lumpur city in Peninsular Malaysia (stars and numbers) together with a table with other information including states, tribes and subtribes. Figure S2. A flow diagram of the total number of subjects (Orang Asli and urban citizens from Kuala Lumpur) involved in both the pre-anthelmintic and post-anthelmintic of this study. Figure S3. A flow diagram summarizing the bioinformatic analysis from raw reads, 1) Quality filtering, remove human reads and adapter (KneadData), taxonomic classification (Kraken2 and Bracken2), 3) K-mer based approach (Sourmash), 4) Estimation of bacterial growth rate (GRiD) to downstream analysis (A–C) such as beta diversity, alpha diversity, effect size estimation and differential abundance, and 5) Functional genes and pathways analysis using HUMAnN v3.0 and its UniRef 50, Pfam, and MetaCyc pathway databases. Figure S4. Difference in the composition of core microbiota between Orang Asli cohort and KL cohort in different taxonomic rank, which include: A Class, B Order, C Family, D Genus, and E Species. Figure S5. Difference in the composition of core microbiota between different geographical location in different taxonomic rank, which include A Family, B Genus, and C Species. Figure S6. A Bar plot of the top 20 species that best predict the difference of the core gut microbiota between Orang Asli (OA) cohort and Kuala Lumpur (KL) cohort using a Random Forest classification model B and C box plots displaying the selected core microbial species that have high variation between Orang Asli (OA) cohort and Kuala Lumpur (KL) cohort based on the Random Forest analysis. The relative abundances of core microbial species between Orang Asli cohort and KL cohort were tested using Wilcoxon rank sum test. B Species with significant higher abundance in Orang Asli cohort than KL cohort, which include (from left to right): HRGM Genome 3145, Gemmiger sp900539695, and [file 40168_2022_1385_MOESM2_ESM.zip › Supplementary_Figure_S17.pdf]

Fig. S18

A

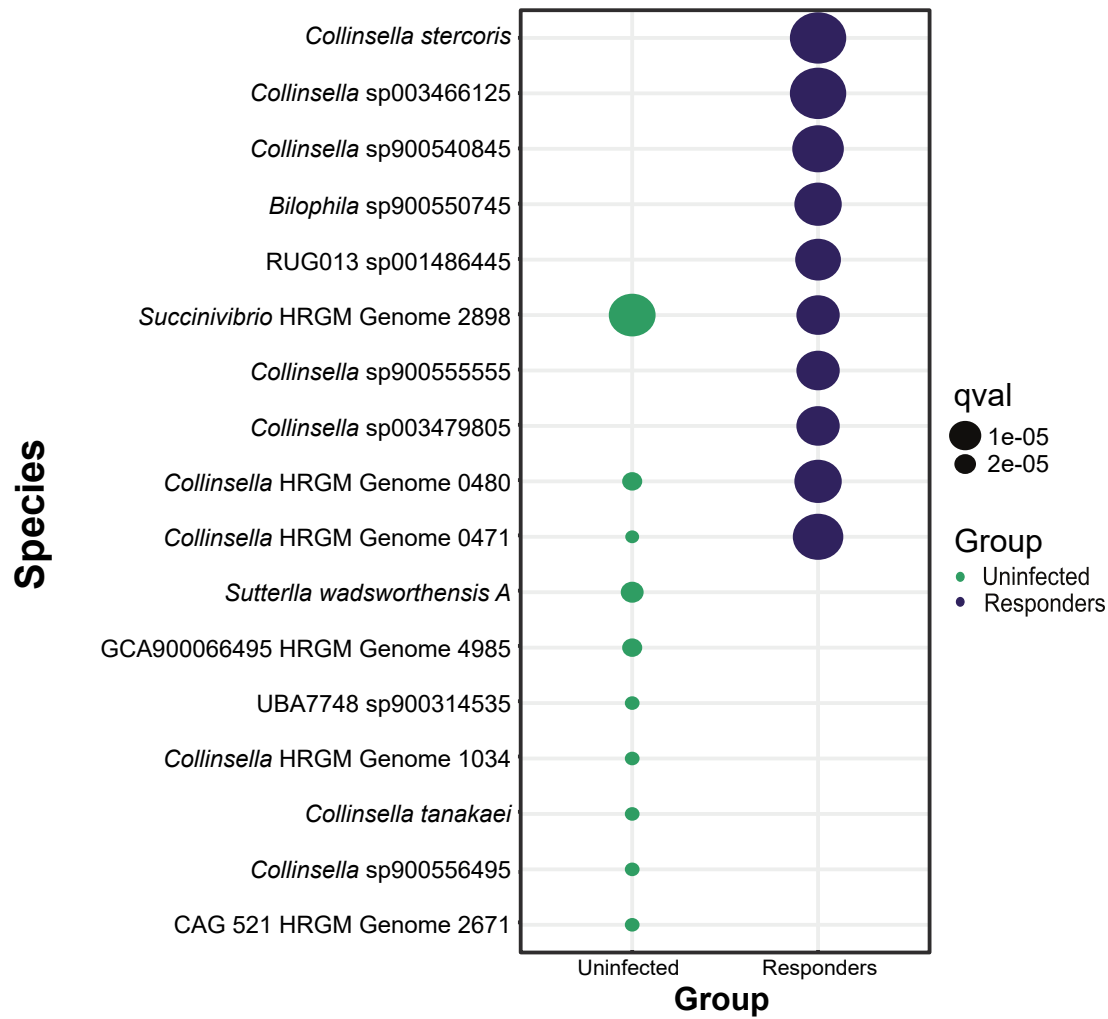

B

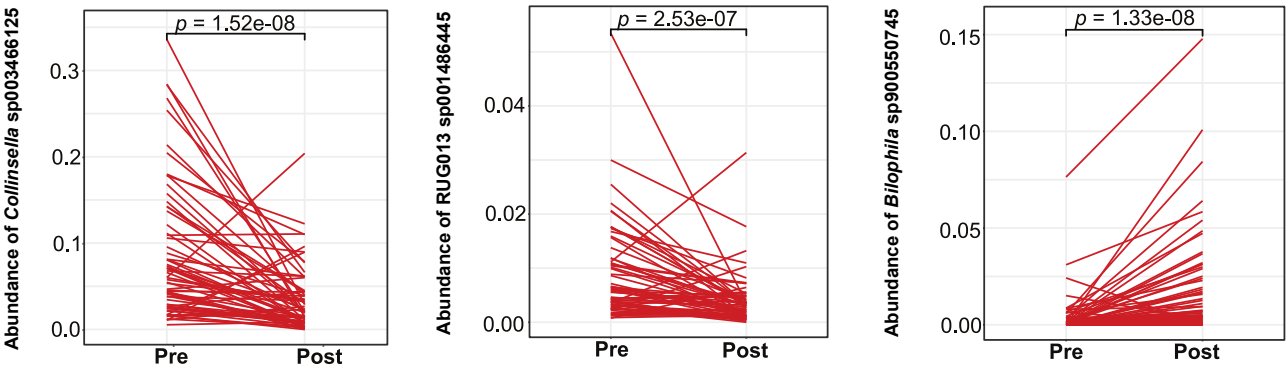

C

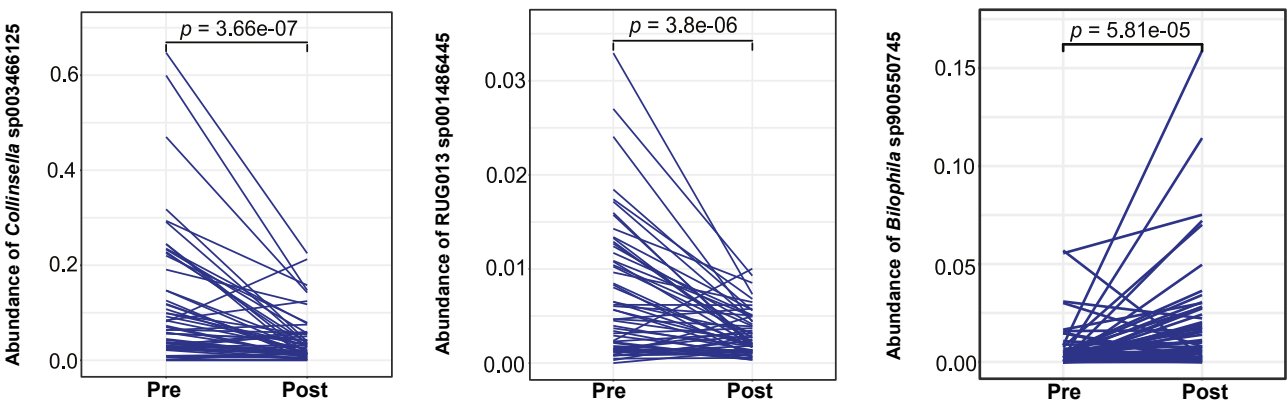

Supplement: Supplementary file 3 — Additional file 2: Figure S1. A geographic map showing the locations of each village and the Kuala Lumpur city in Peninsular Malaysia (stars and numbers) together with a table with other information including states, tribes and subtribes. Figure S2. A flow diagram of the total number of subjects (Orang Asli and urban citizens from Kuala Lumpur) involved in both the pre-anthelmintic and post-anthelmintic of this study. Figure S3. A flow diagram summarizing the bioinformatic analysis from raw reads, 1) Quality filtering, remove human reads and adapter (KneadData), taxonomic classification (Kraken2 and Bracken2), 3) K-mer based approach (Sourmash), 4) Estimation of bacterial growth rate (GRiD) to downstream analysis (A–C) such as beta diversity, alpha diversity, effect size estimation and differential abundance, and 5) Functional genes and pathways analysis using HUMAnN v3.0 and its UniRef 50, Pfam, and MetaCyc pathway databases. Figure S4. Difference in the composition of core microbiota between Orang Asli cohort and KL cohort in different taxonomic rank, which include: A Class, B Order, C Family, D Genus, and E Species. Figure S5. Difference in the composition of core microbiota between different geographical location in different taxonomic rank, which include A Family, B Genus, and C Species. Figure S6. A Bar plot of the top 20 species that best predict the difference of the core gut microbiota between Orang Asli (OA) cohort and Kuala Lumpur (KL) cohort using a Random Forest classification model B and C box plots displaying the selected core microbial species that have high variation between Orang Asli (OA) cohort and Kuala Lumpur (KL) cohort based on the Random Forest analysis. The relative abundances of core microbial species between Orang Asli cohort and KL cohort were tested using Wilcoxon rank sum test. B Species with significant higher abundance in Orang Asli cohort than KL cohort, which include (from left to right): HRGM Genome 3145, Gemmiger sp900539695, and [file 40168_2022_1385_MOESM2_ESM.zip › Supplementary_Figure_S18.pdf]

Fig. S19

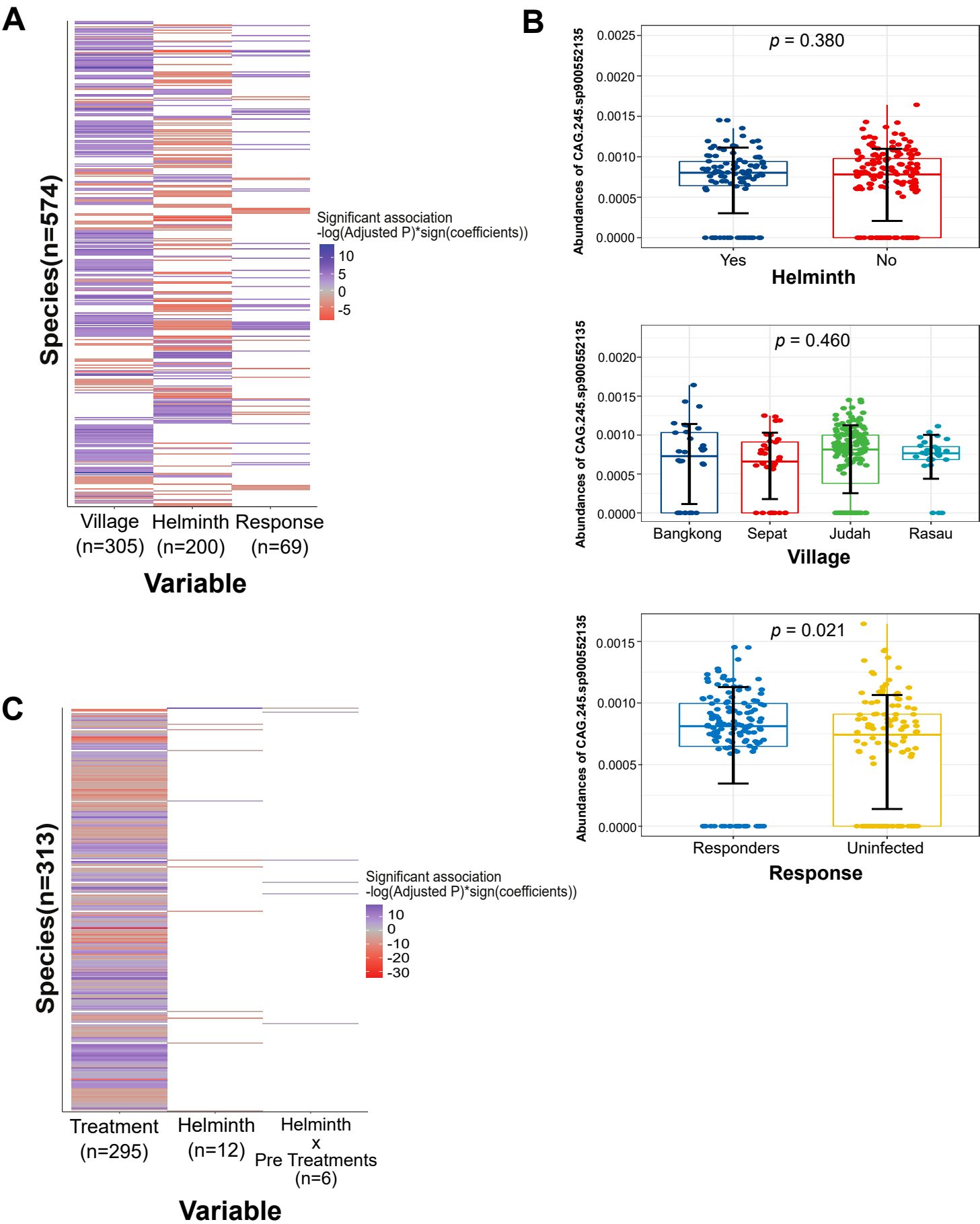

Supplement: Supplementary file 3 — Additional file 2: Figure S1. A geographic map showing the locations of each village and the Kuala Lumpur city in Peninsular Malaysia (stars and numbers) together with a table with other information including states, tribes and subtribes. Figure S2. A flow diagram of the total number of subjects (Orang Asli and urban citizens from Kuala Lumpur) involved in both the pre-anthelmintic and post-anthelmintic of this study. Figure S3. A flow diagram summarizing the bioinformatic analysis from raw reads, 1) Quality filtering, remove human reads and adapter (KneadData), taxonomic classification (Kraken2 and Bracken2), 3) K-mer based approach (Sourmash), 4) Estimation of bacterial growth rate (GRiD) to downstream analysis (A–C) such as beta diversity, alpha diversity, effect size estimation and differential abundance, and 5) Functional genes and pathways analysis using HUMAnN v3.0 and its UniRef 50, Pfam, and MetaCyc pathway databases. Figure S4. Difference in the composition of core microbiota between Orang Asli cohort and KL cohort in different taxonomic rank, which include: A Class, B Order, C Family, D Genus, and E Species. Figure S5. Difference in the composition of core microbiota between different geographical location in different taxonomic rank, which include A Family, B Genus, and C Species. Figure S6. A Bar plot of the top 20 species that best predict the difference of the core gut microbiota between Orang Asli (OA) cohort and Kuala Lumpur (KL) cohort using a Random Forest classification model B and C box plots displaying the selected core microbial species that have high variation between Orang Asli (OA) cohort and Kuala Lumpur (KL) cohort based on the Random Forest analysis. The relative abundances of core microbial species between Orang Asli cohort and KL cohort were tested using Wilcoxon rank sum test. B Species with significant higher abundance in Orang Asli cohort than KL cohort, which include (from left to right): HRGM Genome 3145, Gemmiger sp900539695, and [file 40168_2022_1385_MOESM2_ESM.zip › Supplementary_Figure_S19.pdf]

Fig. S2

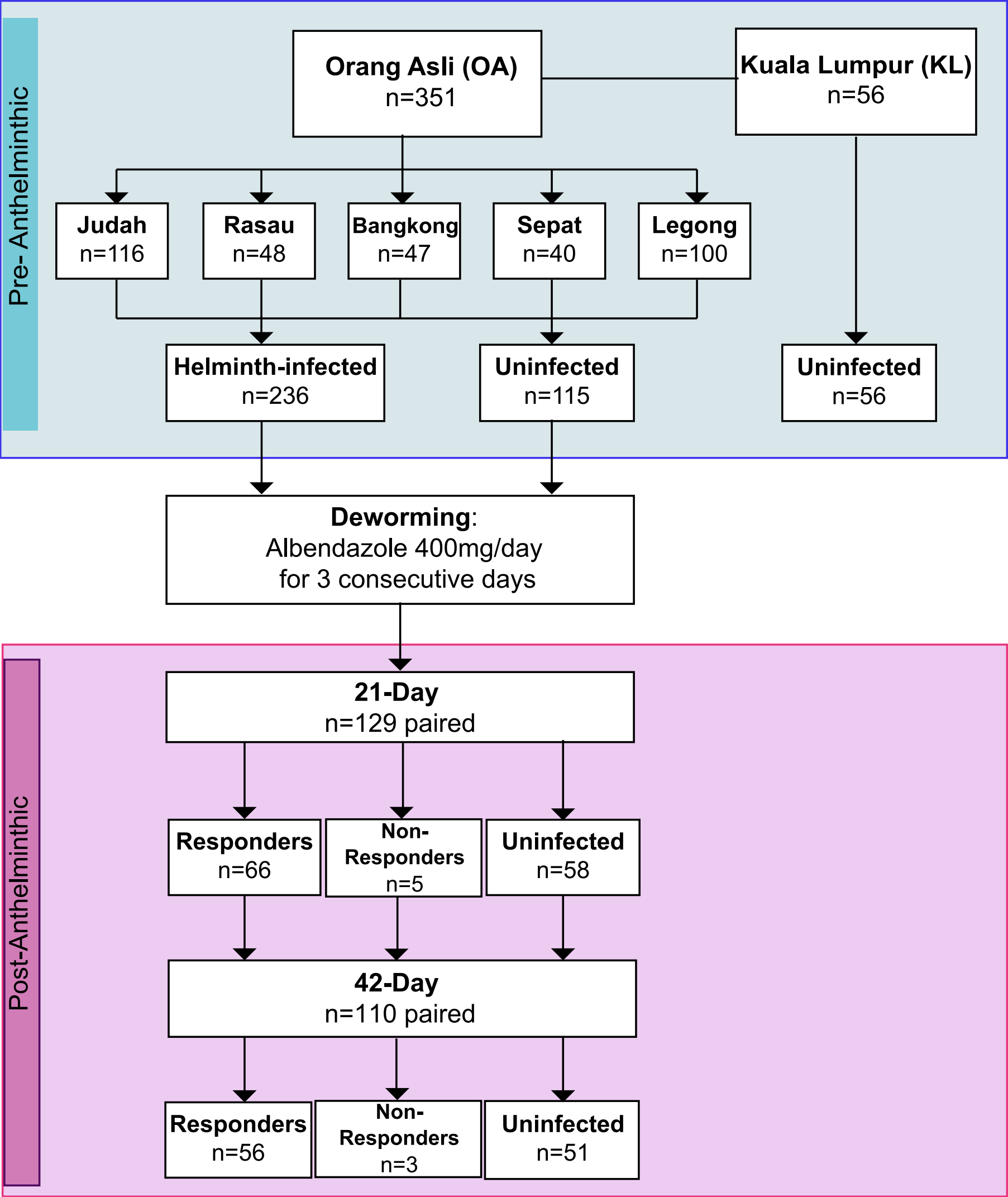

Supplement: Supplementary file 3 — Additional file 2: Figure S1. A geographic map showing the locations of each village and the Kuala Lumpur city in Peninsular Malaysia (stars and numbers) together with a table with other information including states, tribes and subtribes. Figure S2. A flow diagram of the total number of subjects (Orang Asli and urban citizens from Kuala Lumpur) involved in both the pre-anthelmintic and post-anthelmintic of this study. Figure S3. A flow diagram summarizing the bioinformatic analysis from raw reads, 1) Quality filtering, remove human reads and adapter (KneadData), taxonomic classification (Kraken2 and Bracken2), 3) K-mer based approach (Sourmash), 4) Estimation of bacterial growth rate (GRiD) to downstream analysis (A–C) such as beta diversity, alpha diversity, effect size estimation and differential abundance, and 5) Functional genes and pathways analysis using HUMAnN v3.0 and its UniRef 50, Pfam, and MetaCyc pathway databases. Figure S4. Difference in the composition of core microbiota between Orang Asli cohort and KL cohort in different taxonomic rank, which include: A Class, B Order, C Family, D Genus, and E Species. Figure S5. Difference in the composition of core microbiota between different geographical location in different taxonomic rank, which include A Family, B Genus, and C Species. Figure S6. A Bar plot of the top 20 species that best predict the difference of the core gut microbiota between Orang Asli (OA) cohort and Kuala Lumpur (KL) cohort using a Random Forest classification model B and C box plots displaying the selected core microbial species that have high variation between Orang Asli (OA) cohort and Kuala Lumpur (KL) cohort based on the Random Forest analysis. The relative abundances of core microbial species between Orang Asli cohort and KL cohort were tested using Wilcoxon rank sum test. B Species with significant higher abundance in Orang Asli cohort than KL cohort, which include (from left to right): HRGM Genome 3145, Gemmiger sp900539695, and [file 40168_2022_1385_MOESM2_ESM.zip › Supplementary_Figure_S2.pdf]

**Fig. S20**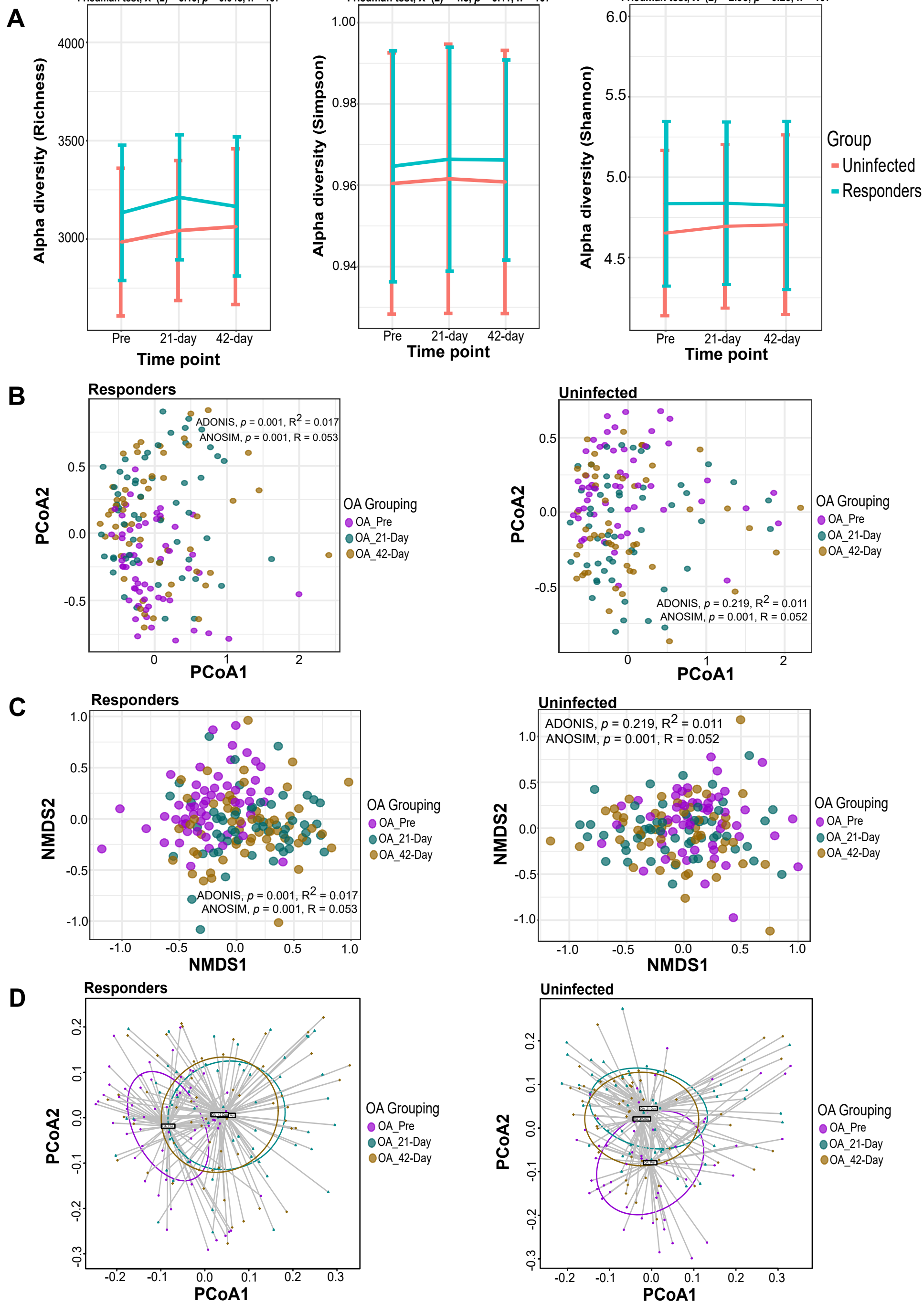

Supplement: Supplementary file 3 — Additional file 2: Figure S1. A geographic map showing the locations of each village and the Kuala Lumpur city in Peninsular Malaysia (stars and numbers) together with a table with other information including states, tribes and subtribes. Figure S2. A flow diagram of the total number of subjects (Orang Asli and urban citizens from Kuala Lumpur) involved in both the pre-anthelmintic and post-anthelmintic of this study. Figure S3. A flow diagram summarizing the bioinformatic analysis from raw reads, 1) Quality filtering, remove human reads and adapter (KneadData), taxonomic classification (Kraken2 and Bracken2), 3) K-mer based approach (Sourmash), 4) Estimation of bacterial growth rate (GRiD) to downstream analysis (A–C) such as beta diversity, alpha diversity, effect size estimation and differential abundance, and 5) Functional genes and pathways analysis using HUMAnN v3.0 and its UniRef 50, Pfam, and MetaCyc pathway databases. Figure S4. Difference in the composition of core microbiota between Orang Asli cohort and KL cohort in different taxonomic rank, which include: A Class, B Order, C Family, D Genus, and E Species. Figure S5. Difference in the composition of core microbiota between different geographical location in different taxonomic rank, which include A Family, B Genus, and C Species. Figure S6. A Bar plot of the top 20 species that best predict the difference of the core gut microbiota between Orang Asli (OA) cohort and Kuala Lumpur (KL) cohort using a Random Forest classification model B and C box plots displaying the selected core microbial species that have high variation between Orang Asli (OA) cohort and Kuala Lumpur (KL) cohort based on the Random Forest analysis. The relative abundances of core microbial species between Orang Asli cohort and KL cohort were tested using Wilcoxon rank sum test. B Species with significant higher abundance in Orang Asli cohort than KL cohort, which include (from left to right): HRGM Genome 3145, Gemmiger sp900539695, and [file 40168_2022_1385_MOESM2_ESM.zip › Supplementary_Figure_S20.pdf]

Fig. S21

A

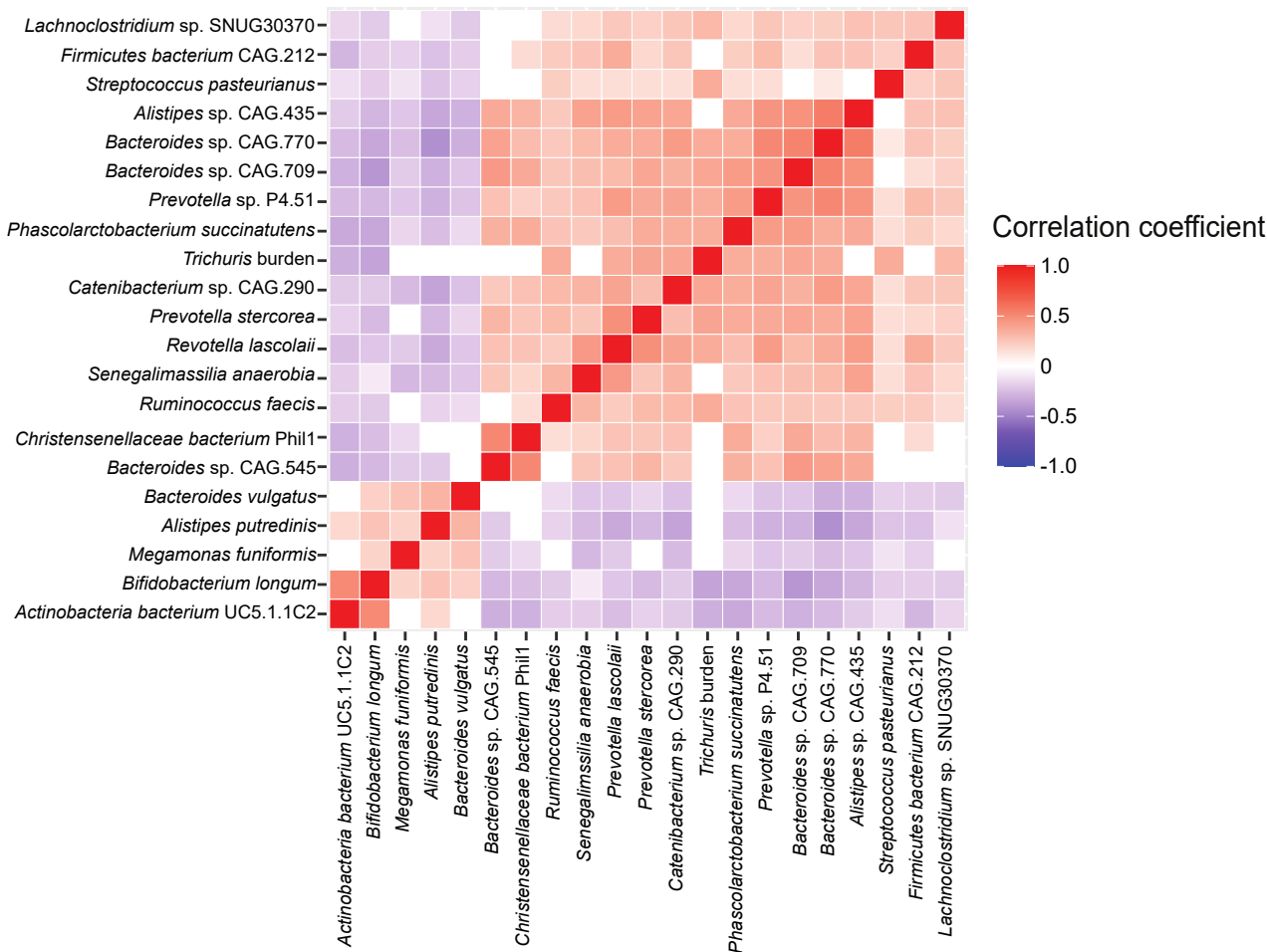

B

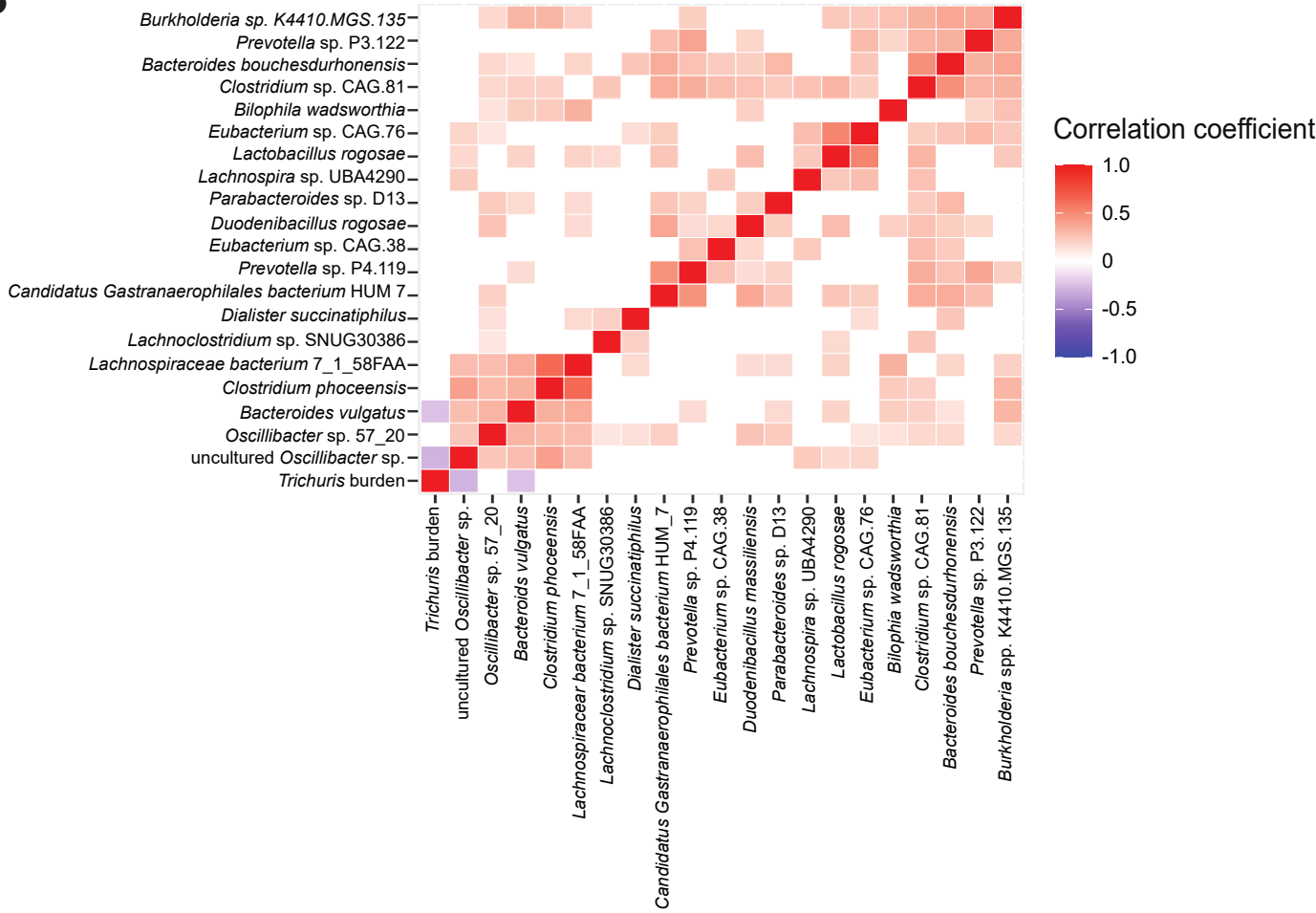

Supplement: Supplementary file 3 — Additional file 2: Figure S1. A geographic map showing the locations of each village and the Kuala Lumpur city in Peninsular Malaysia (stars and numbers) together with a table with other information including states, tribes and subtribes. Figure S2. A flow diagram of the total number of subjects (Orang Asli and urban citizens from Kuala Lumpur) involved in both the pre-anthelmintic and post-anthelmintic of this study. Figure S3. A flow diagram summarizing the bioinformatic analysis from raw reads, 1) Quality filtering, remove human reads and adapter (KneadData), taxonomic classification (Kraken2 and Bracken2), 3) K-mer based approach (Sourmash), 4) Estimation of bacterial growth rate (GRiD) to downstream analysis (A–C) such as beta diversity, alpha diversity, effect size estimation and differential abundance, and 5) Functional genes and pathways analysis using HUMAnN v3.0 and its UniRef 50, Pfam, and MetaCyc pathway databases. Figure S4. Difference in the composition of core microbiota between Orang Asli cohort and KL cohort in different taxonomic rank, which include: A Class, B Order, C Family, D Genus, and E Species. Figure S5. Difference in the composition of core microbiota between different geographical location in different taxonomic rank, which include A Family, B Genus, and C Species. Figure S6. A Bar plot of the top 20 species that best predict the difference of the core gut microbiota between Orang Asli (OA) cohort and Kuala Lumpur (KL) cohort using a Random Forest classification model B and C box plots displaying the selected core microbial species that have high variation between Orang Asli (OA) cohort and Kuala Lumpur (KL) cohort based on the Random Forest analysis. The relative abundances of core microbial species between Orang Asli cohort and KL cohort were tested using Wilcoxon rank sum test. B Species with significant higher abundance in Orang Asli cohort than KL cohort, which include (from left to right): HRGM Genome 3145, Gemmiger sp900539695, and [file 40168_2022_1385_MOESM2_ESM.zip › Supplementary_Figure_S21.pdf]

Fig. S22

A

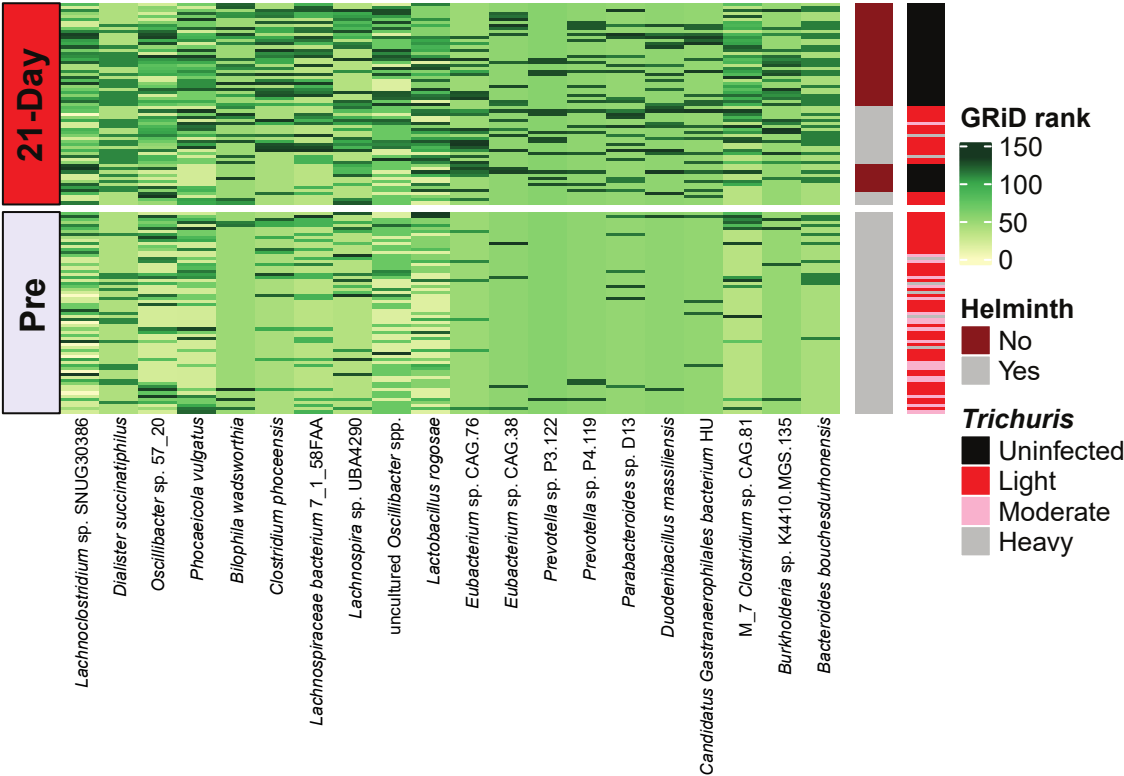

B

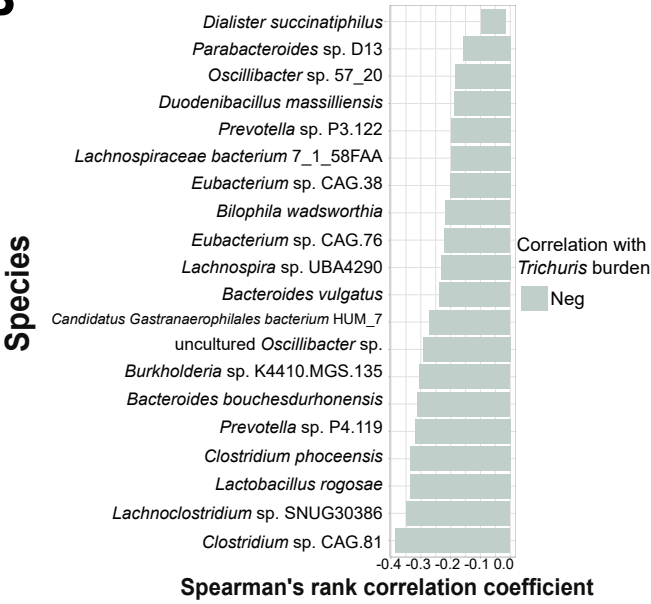

C

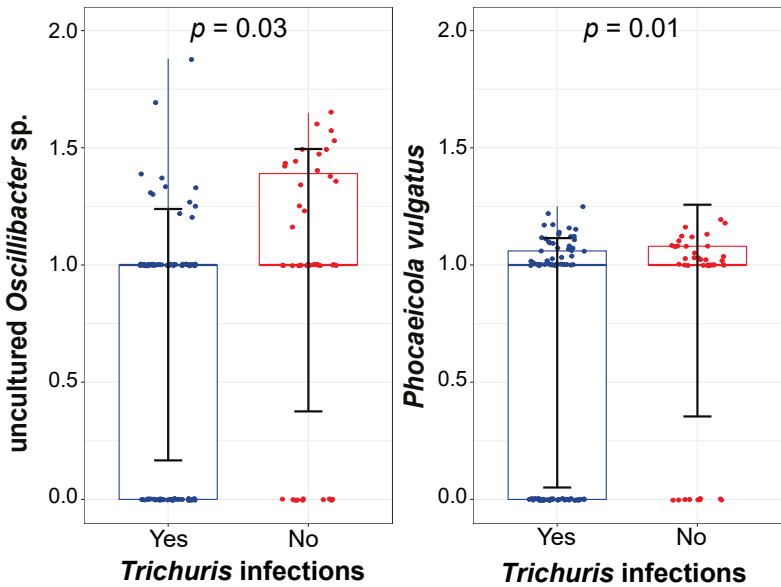

D

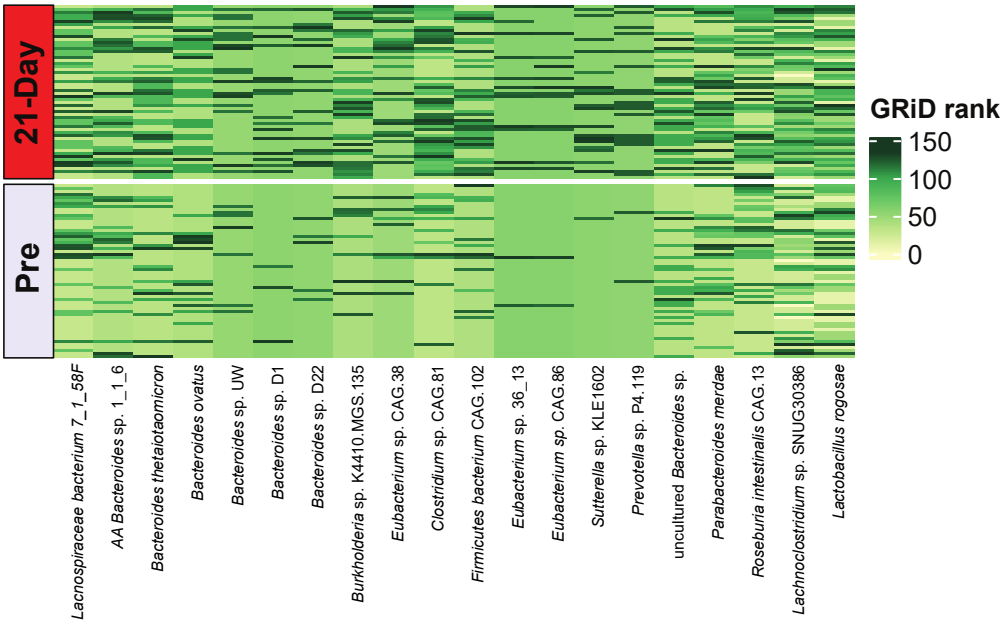

Supplement: Supplementary file 3 — Additional file 2: Figure S1. A geographic map showing the locations of each village and the Kuala Lumpur city in Peninsular Malaysia (stars and numbers) together with a table with other information including states, tribes and subtribes. Figure S2. A flow diagram of the total number of subjects (Orang Asli and urban citizens from Kuala Lumpur) involved in both the pre-anthelmintic and post-anthelmintic of this study. Figure S3. A flow diagram summarizing the bioinformatic analysis from raw reads, 1) Quality filtering, remove human reads and adapter (KneadData), taxonomic classification (Kraken2 and Bracken2), 3) K-mer based approach (Sourmash), 4) Estimation of bacterial growth rate (GRiD) to downstream analysis (A–C) such as beta diversity, alpha diversity, effect size estimation and differential abundance, and 5) Functional genes and pathways analysis using HUMAnN v3.0 and its UniRef 50, Pfam, and MetaCyc pathway databases. Figure S4. Difference in the composition of core microbiota between Orang Asli cohort and KL cohort in different taxonomic rank, which include: A Class, B Order, C Family, D Genus, and E Species. Figure S5. Difference in the composition of core microbiota between different geographical location in different taxonomic rank, which include A Family, B Genus, and C Species. Figure S6. A Bar plot of the top 20 species that best predict the difference of the core gut microbiota between Orang Asli (OA) cohort and Kuala Lumpur (KL) cohort using a Random Forest classification model B and C box plots displaying the selected core microbial species that have high variation between Orang Asli (OA) cohort and Kuala Lumpur (KL) cohort based on the Random Forest analysis. The relative abundances of core microbial species between Orang Asli cohort and KL cohort were tested using Wilcoxon rank sum test. B Species with significant higher abundance in Orang Asli cohort than KL cohort, which include (from left to right): HRGM Genome 3145, Gemmiger sp900539695, and [file 40168_2022_1385_MOESM2_ESM.zip › Supplementary_Figure_S22.pdf]

**Fig. S25**

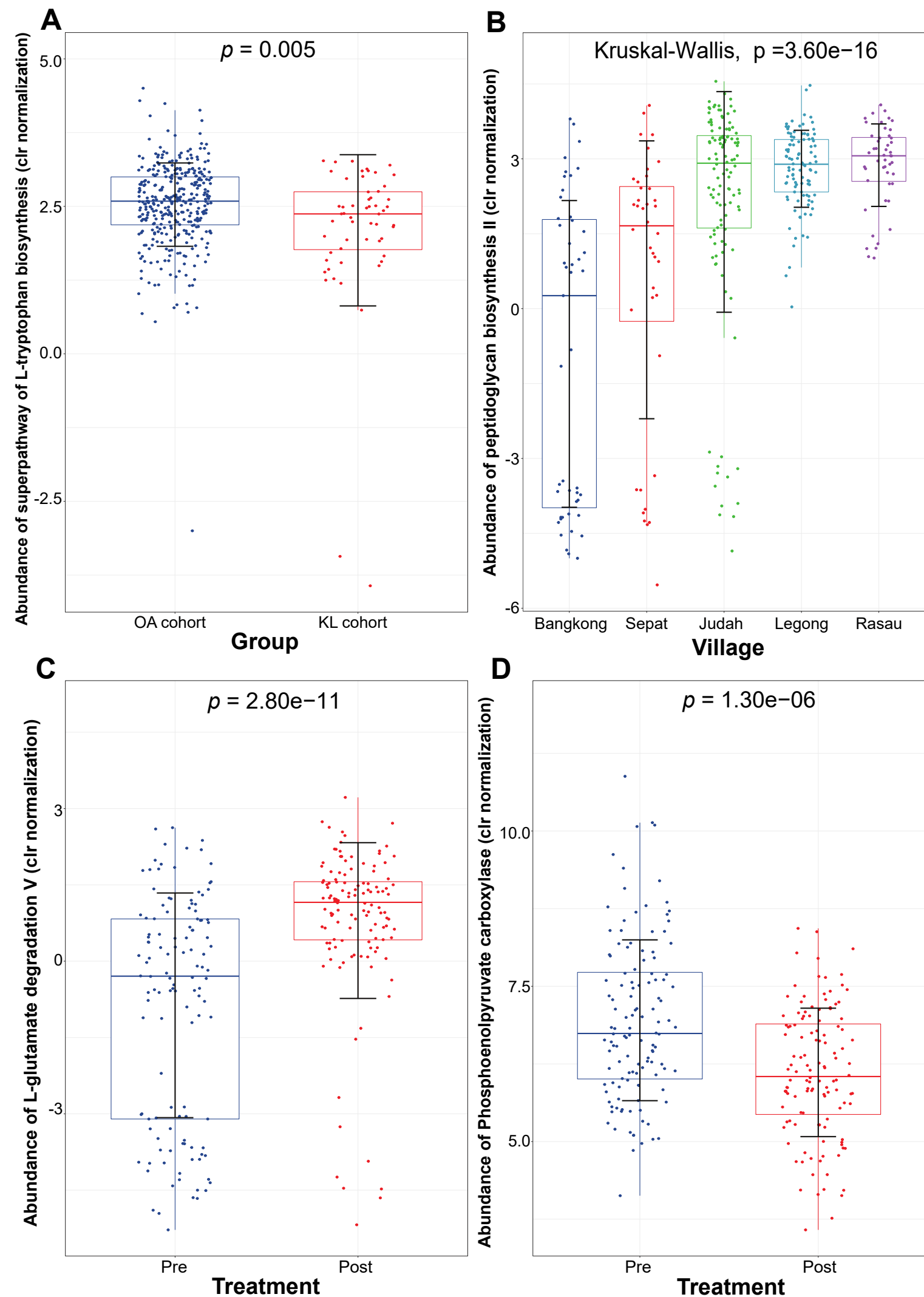

Supplement: Supplementary file 3 — Additional file 2: Figure S1. A geographic map showing the locations of each village and the Kuala Lumpur city in Peninsular Malaysia (stars and numbers) together with a table with other information including states, tribes and subtribes. Figure S2. A flow diagram of the total number of subjects (Orang Asli and urban citizens from Kuala Lumpur) involved in both the pre-anthelmintic and post-anthelmintic of this study. Figure S3. A flow diagram summarizing the bioinformatic analysis from raw reads, 1) Quality filtering, remove human reads and adapter (KneadData), taxonomic classification (Kraken2 and Bracken2), 3) K-mer based approach (Sourmash), 4) Estimation of bacterial growth rate (GRiD) to downstream analysis (A–C) such as beta diversity, alpha diversity, effect size estimation and differential abundance, and 5) Functional genes and pathways analysis using HUMAnN v3.0 and its UniRef 50, Pfam, and MetaCyc pathway databases. Figure S4. Difference in the composition of core microbiota between Orang Asli cohort and KL cohort in different taxonomic rank, which include: A Class, B Order, C Family, D Genus, and E Species. Figure S5. Difference in the composition of core microbiota between different geographical location in different taxonomic rank, which include A Family, B Genus, and C Species. Figure S6. A Bar plot of the top 20 species that best predict the difference of the core gut microbiota between Orang Asli (OA) cohort and Kuala Lumpur (KL) cohort using a Random Forest classification model B and C box plots displaying the selected core microbial species that have high variation between Orang Asli (OA) cohort and Kuala Lumpur (KL) cohort based on the Random Forest analysis. The relative abundances of core microbial species between Orang Asli cohort and KL cohort were tested using Wilcoxon rank sum test. B Species with significant higher abundance in Orang Asli cohort than KL cohort, which include (from left to right): HRGM Genome 3145, Gemmiger sp900539695, and [file 40168_2022_1385_MOESM2_ESM.zip › Supplementary_Figure_S25.pdf]

Fig. S26

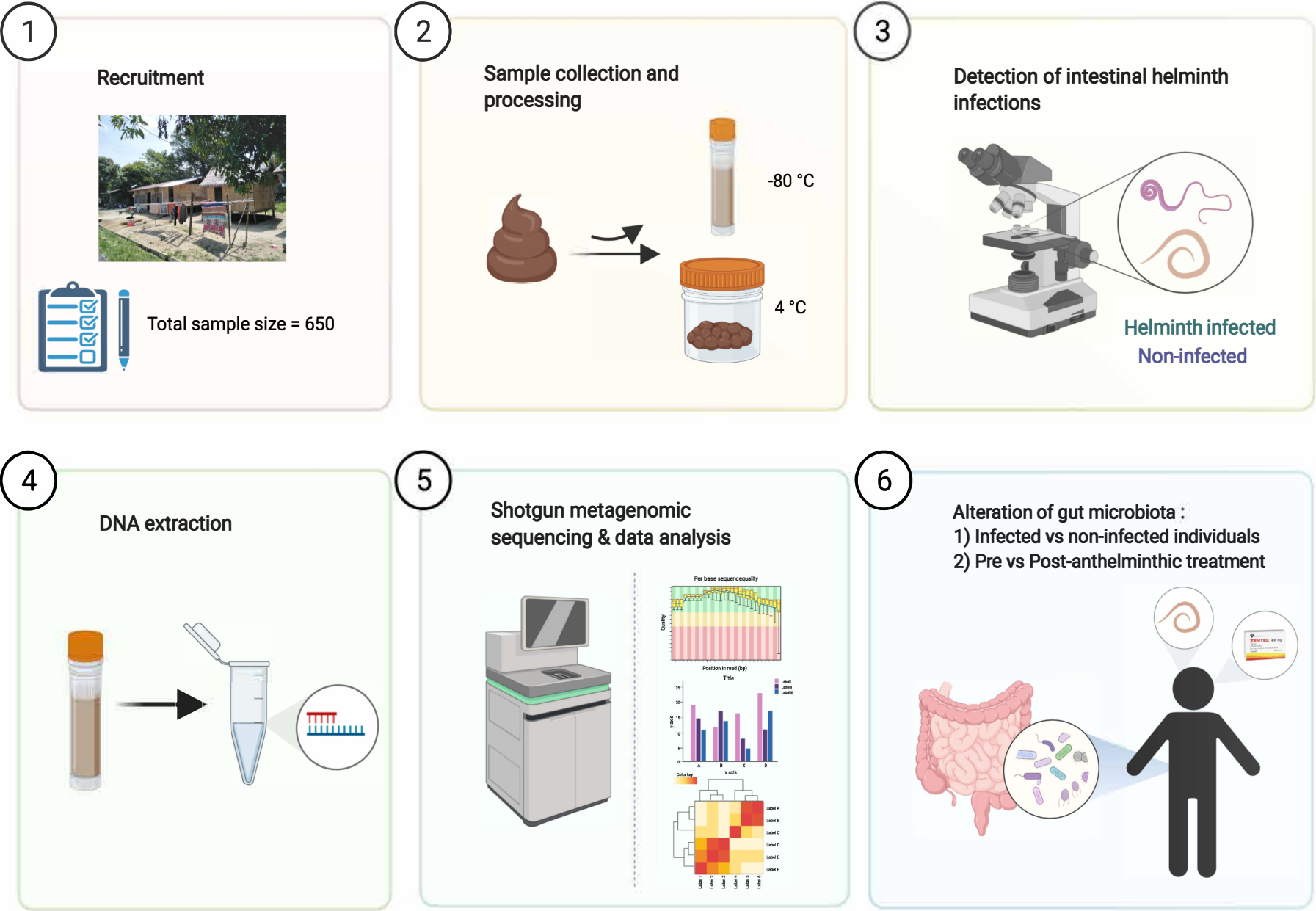

Supplement: Supplementary file 3 — Additional file 2: Figure S1. A geographic map showing the locations of each village and the Kuala Lumpur city in Peninsular Malaysia (stars and numbers) together with a table with other information including states, tribes and subtribes. Figure S2. A flow diagram of the total number of subjects (Orang Asli and urban citizens from Kuala Lumpur) involved in both the pre-anthelmintic and post-anthelmintic of this study. Figure S3. A flow diagram summarizing the bioinformatic analysis from raw reads, 1) Quality filtering, remove human reads and adapter (KneadData), taxonomic classification (Kraken2 and Bracken2), 3) K-mer based approach (Sourmash), 4) Estimation of bacterial growth rate (GRiD) to downstream analysis (A–C) such as beta diversity, alpha diversity, effect size estimation and differential abundance, and 5) Functional genes and pathways analysis using HUMAnN v3.0 and its UniRef 50, Pfam, and MetaCyc pathway databases. Figure S4. Difference in the composition of core microbiota between Orang Asli cohort and KL cohort in different taxonomic rank, which include: A Class, B Order, C Family, D Genus, and E Species. Figure S5. Difference in the composition of core microbiota between different geographical location in different taxonomic rank, which include A Family, B Genus, and C Species. Figure S6. A Bar plot of the top 20 species that best predict the difference of the core gut microbiota between Orang Asli (OA) cohort and Kuala Lumpur (KL) cohort using a Random Forest classification model B and C box plots displaying the selected core microbial species that have high variation between Orang Asli (OA) cohort and Kuala Lumpur (KL) cohort based on the Random Forest analysis. The relative abundances of core microbial species between Orang Asli cohort and KL cohort were tested using Wilcoxon rank sum test. B Species with significant higher abundance in Orang Asli cohort than KL cohort, which include (from left to right): HRGM Genome 3145, Gemmiger sp900539695, and [file 40168_2022_1385_MOESM2_ESM.zip › Supplementary_Figure_S26.pdf]

**Fig. S27**

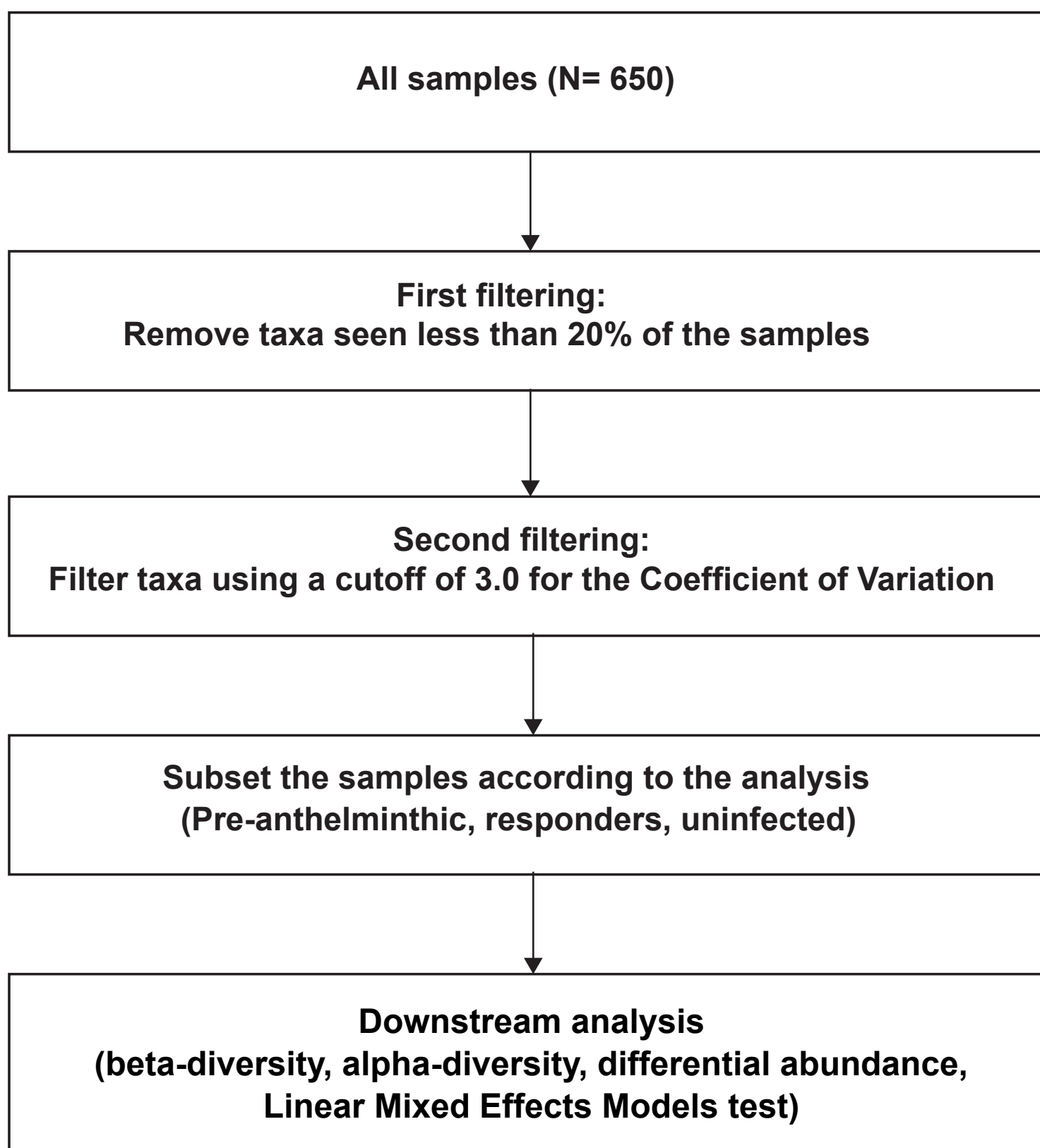

Supplement: Supplementary file 3 — Additional file 2: Figure S1. A geographic map showing the locations of each village and the Kuala Lumpur city in Peninsular Malaysia (stars and numbers) together with a table with other information including states, tribes and subtribes. Figure S2. A flow diagram of the total number of subjects (Orang Asli and urban citizens from Kuala Lumpur) involved in both the pre-anthelmintic and post-anthelmintic of this study. Figure S3. A flow diagram summarizing the bioinformatic analysis from raw reads, 1) Quality filtering, remove human reads and adapter (KneadData), taxonomic classification (Kraken2 and Bracken2), 3) K-mer based approach (Sourmash), 4) Estimation of bacterial growth rate (GRiD) to downstream analysis (A–C) such as beta diversity, alpha diversity, effect size estimation and differential abundance, and 5) Functional genes and pathways analysis using HUMAnN v3.0 and its UniRef 50, Pfam, and MetaCyc pathway databases. Figure S4. Difference in the composition of core microbiota between Orang Asli cohort and KL cohort in different taxonomic rank, which include: A Class, B Order, C Family, D Genus, and E Species. Figure S5. Difference in the composition of core microbiota between different geographical location in different taxonomic rank, which include A Family, B Genus, and C Species. Figure S6. A Bar plot of the top 20 species that best predict the difference of the core gut microbiota between Orang Asli (OA) cohort and Kuala Lumpur (KL) cohort using a Random Forest classification model B and C box plots displaying the selected core microbial species that have high variation between Orang Asli (OA) cohort and Kuala Lumpur (KL) cohort based on the Random Forest analysis. The relative abundances of core microbial species between Orang Asli cohort and KL cohort were tested using Wilcoxon rank sum test. B Species with significant higher abundance in Orang Asli cohort than KL cohort, which include (from left to right): HRGM Genome 3145, Gemmiger sp900539695, and [file 40168_2022_1385_MOESM2_ESM.zip › Supplementary_Figure_S27.pdf]

**Fig. S28**

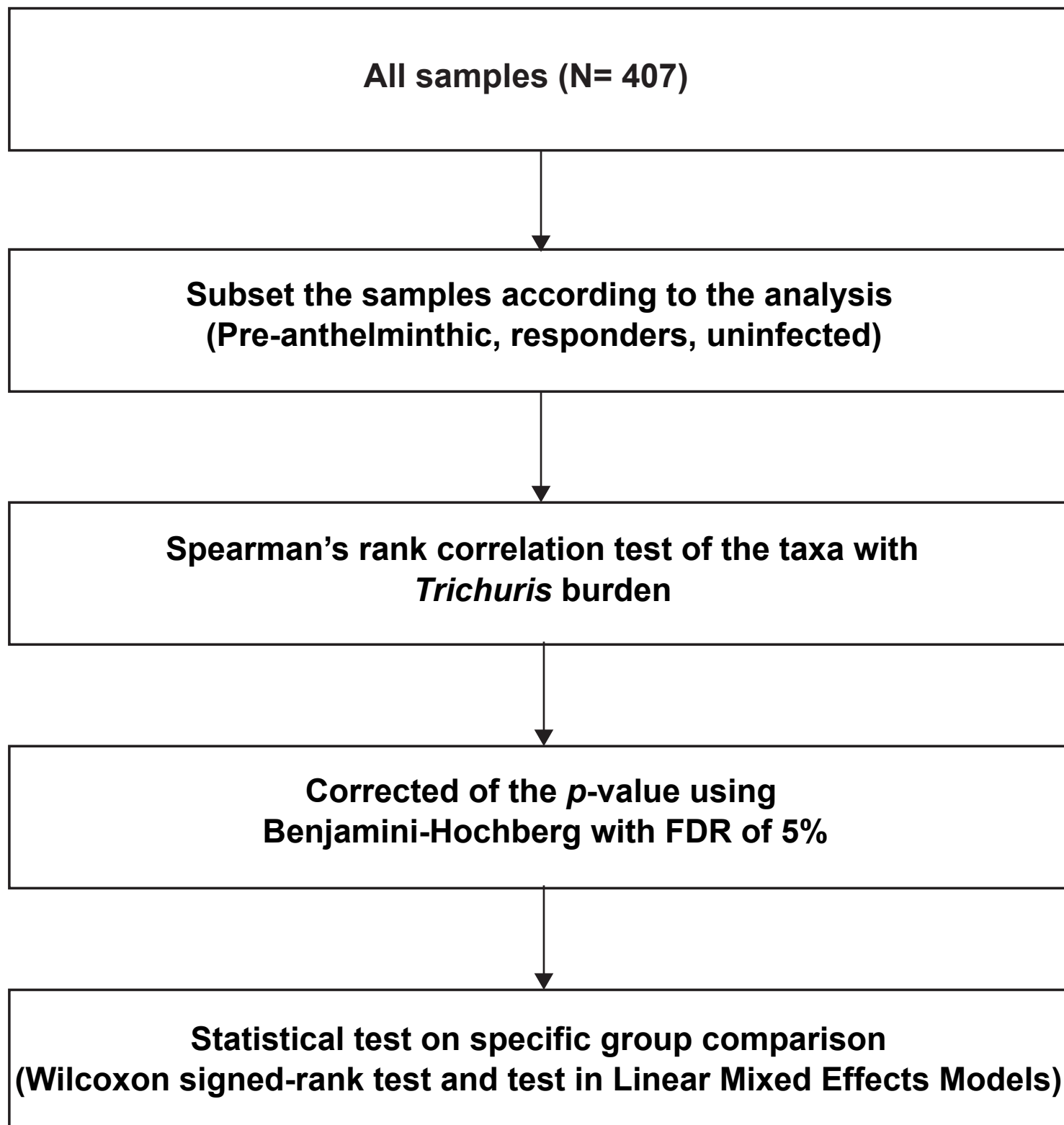

Supplement: Supplementary file 3 — Additional file 2: Figure S1. A geographic map showing the locations of each village and the Kuala Lumpur city in Peninsular Malaysia (stars and numbers) together with a table with other information including states, tribes and subtribes. Figure S2. A flow diagram of the total number of subjects (Orang Asli and urban citizens from Kuala Lumpur) involved in both the pre-anthelmintic and post-anthelmintic of this study. Figure S3. A flow diagram summarizing the bioinformatic analysis from raw reads, 1) Quality filtering, remove human reads and adapter (KneadData), taxonomic classification (Kraken2 and Bracken2), 3) K-mer based approach (Sourmash), 4) Estimation of bacterial growth rate (GRiD) to downstream analysis (A–C) such as beta diversity, alpha diversity, effect size estimation and differential abundance, and 5) Functional genes and pathways analysis using HUMAnN v3.0 and its UniRef 50, Pfam, and MetaCyc pathway databases. Figure S4. Difference in the composition of core microbiota between Orang Asli cohort and KL cohort in different taxonomic rank, which include: A Class, B Order, C Family, D Genus, and E Species. Figure S5. Difference in the composition of core microbiota between different geographical location in different taxonomic rank, which include A Family, B Genus, and C Species. Figure S6. A Bar plot of the top 20 species that best predict the difference of the core gut microbiota between Orang Asli (OA) cohort and Kuala Lumpur (KL) cohort using a Random Forest classification model B and C box plots displaying the selected core microbial species that have high variation between Orang Asli (OA) cohort and Kuala Lumpur (KL) cohort based on the Random Forest analysis. The relative abundances of core microbial species between Orang Asli cohort and KL cohort were tested using Wilcoxon rank sum test. B Species with significant higher abundance in Orang Asli cohort than KL cohort, which include (from left to right): HRGM Genome 3145, Gemmiger sp900539695, and [file 40168_2022_1385_MOESM2_ESM.zip › Supplementary_Figure_S28.pdf]

Fig. S3

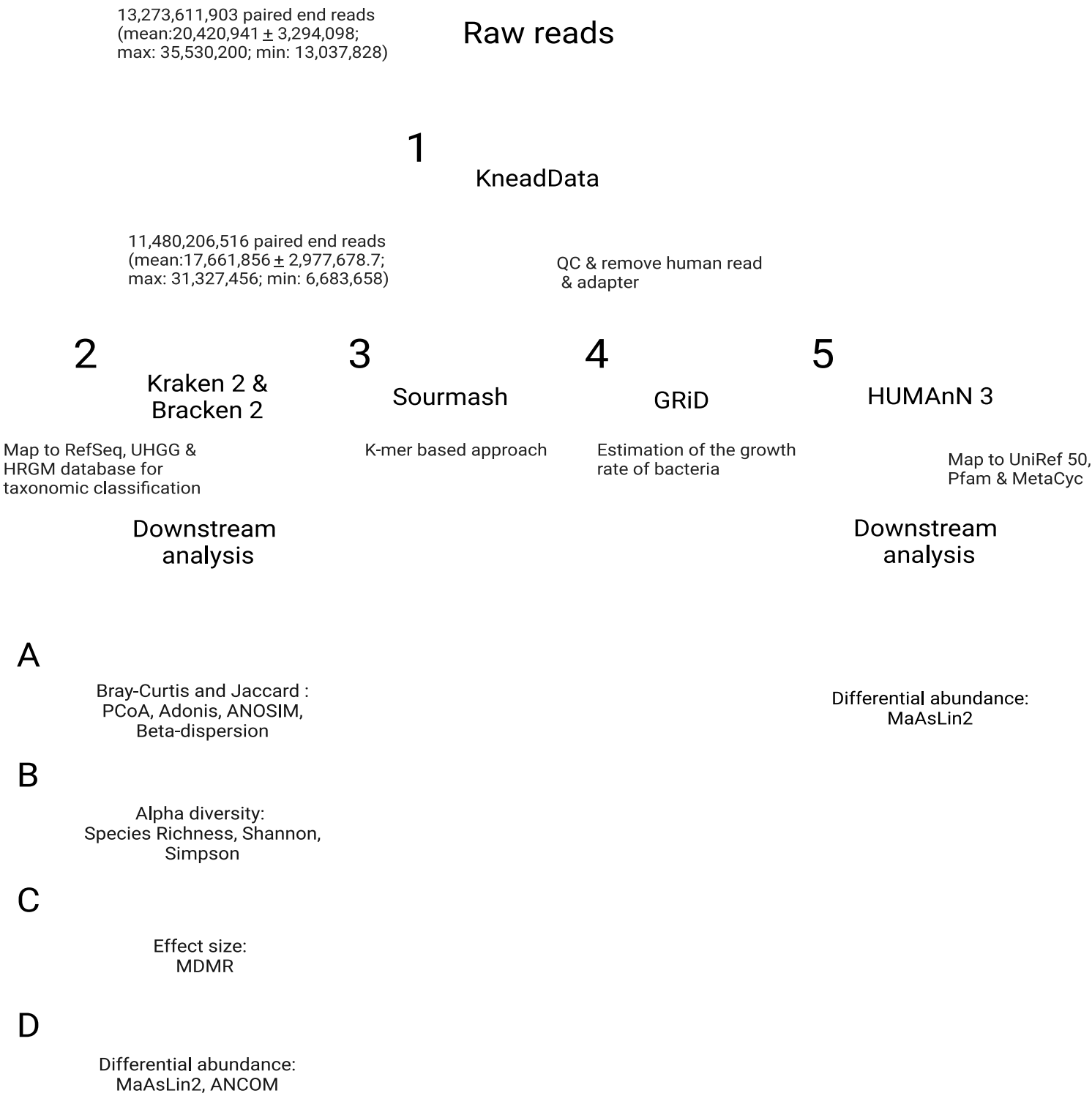

Supplement: Supplementary file 3 — Additional file 2: Figure S1. A geographic map showing the locations of each village and the Kuala Lumpur city in Peninsular Malaysia (stars and numbers) together with a table with other information including states, tribes and subtribes. Figure S2. A flow diagram of the total number of subjects (Orang Asli and urban citizens from Kuala Lumpur) involved in both the pre-anthelmintic and post-anthelmintic of this study. Figure S3. A flow diagram summarizing the bioinformatic analysis from raw reads, 1) Quality filtering, remove human reads and adapter (KneadData), taxonomic classification (Kraken2 and Bracken2), 3) K-mer based approach (Sourmash), 4) Estimation of bacterial growth rate (GRiD) to downstream analysis (A–C) such as beta diversity, alpha diversity, effect size estimation and differential abundance, and 5) Functional genes and pathways analysis using HUMAnN v3.0 and its UniRef 50, Pfam, and MetaCyc pathway databases. Figure S4. Difference in the composition of core microbiota between Orang Asli cohort and KL cohort in different taxonomic rank, which include: A Class, B Order, C Family, D Genus, and E Species. Figure S5. Difference in the composition of core microbiota between different geographical location in different taxonomic rank, which include A Family, B Genus, and C Species. Figure S6. A Bar plot of the top 20 species that best predict the difference of the core gut microbiota between Orang Asli (OA) cohort and Kuala Lumpur (KL) cohort using a Random Forest classification model B and C box plots displaying the selected core microbial species that have high variation between Orang Asli (OA) cohort and Kuala Lumpur (KL) cohort based on the Random Forest analysis. The relative abundances of core microbial species between Orang Asli cohort and KL cohort were tested using Wilcoxon rank sum test. B Species with significant higher abundance in Orang Asli cohort than KL cohort, which include (from left to right): HRGM Genome 3145, Gemmiger sp900539695, and [file 40168_2022_1385_MOESM2_ESM.zip › Supplementary_Figure_S3.pdf]

Fig. S4

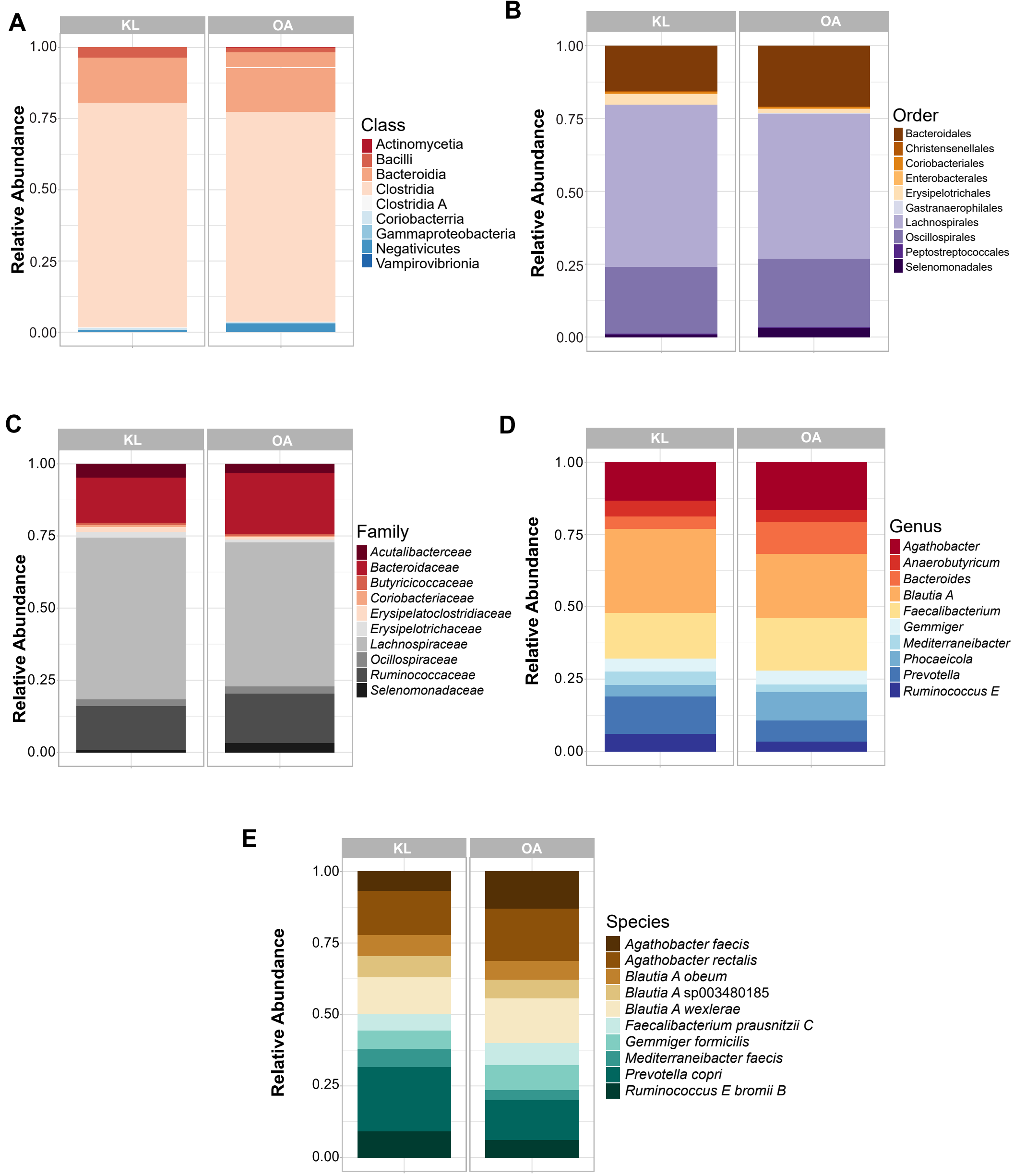

Supplement: Supplementary file 3 — Additional file 2: Figure S1. A geographic map showing the locations of each village and the Kuala Lumpur city in Peninsular Malaysia (stars and numbers) together with a table with other information including states, tribes and subtribes. Figure S2. A flow diagram of the total number of subjects (Orang Asli and urban citizens from Kuala Lumpur) involved in both the pre-anthelmintic and post-anthelmintic of this study. Figure S3. A flow diagram summarizing the bioinformatic analysis from raw reads, 1) Quality filtering, remove human reads and adapter (KneadData), taxonomic classification (Kraken2 and Bracken2), 3) K-mer based approach (Sourmash), 4) Estimation of bacterial growth rate (GRiD) to downstream analysis (A–C) such as beta diversity, alpha diversity, effect size estimation and differential abundance, and 5) Functional genes and pathways analysis using HUMAnN v3.0 and its UniRef 50, Pfam, and MetaCyc pathway databases. Figure S4. Difference in the composition of core microbiota between Orang Asli cohort and KL cohort in different taxonomic rank, which include: A Class, B Order, C Family, D Genus, and E Species. Figure S5. Difference in the composition of core microbiota between different geographical location in different taxonomic rank, which include A Family, B Genus, and C Species. Figure S6. A Bar plot of the top 20 species that best predict the difference of the core gut microbiota between Orang Asli (OA) cohort and Kuala Lumpur (KL) cohort using a Random Forest classification model B and C box plots displaying the selected core microbial species that have high variation between Orang Asli (OA) cohort and Kuala Lumpur (KL) cohort based on the Random Forest analysis. The relative abundances of core microbial species between Orang Asli cohort and KL cohort were tested using Wilcoxon rank sum test. B Species with significant higher abundance in Orang Asli cohort than KL cohort, which include (from left to right): HRGM Genome 3145, Gemmiger sp900539695, and [file 40168_2022_1385_MOESM2_ESM.zip › Supplementary_Figure_S4.pdf]

Fig. S5

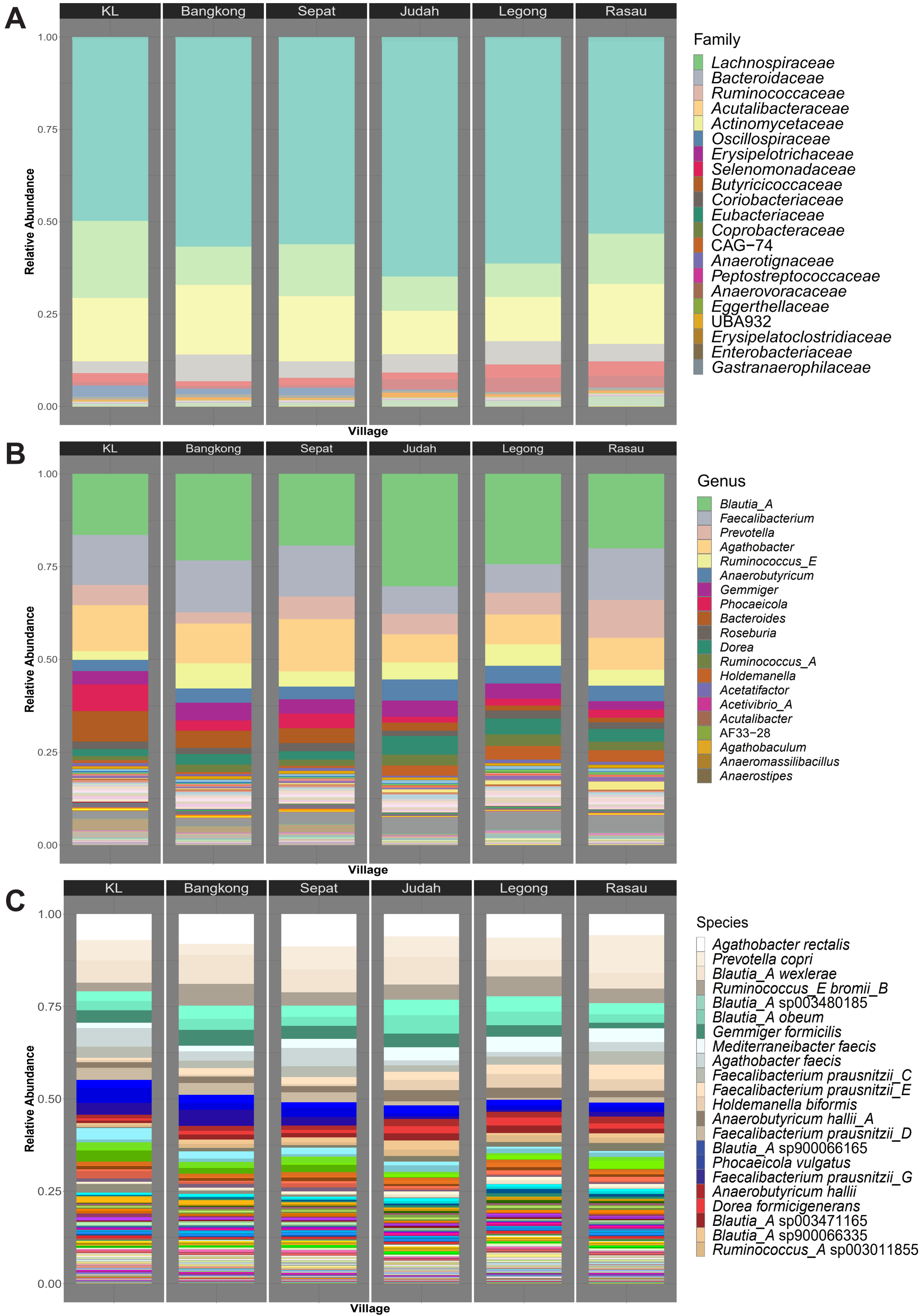

Supplement: Supplementary file 3 — Additional file 2: Figure S1. A geographic map showing the locations of each village and the Kuala Lumpur city in Peninsular Malaysia (stars and numbers) together with a table with other information including states, tribes and subtribes. Figure S2. A flow diagram of the total number of subjects (Orang Asli and urban citizens from Kuala Lumpur) involved in both the pre-anthelmintic and post-anthelmintic of this study. Figure S3. A flow diagram summarizing the bioinformatic analysis from raw reads, 1) Quality filtering, remove human reads and adapter (KneadData), taxonomic classification (Kraken2 and Bracken2), 3) K-mer based approach (Sourmash), 4) Estimation of bacterial growth rate (GRiD) to downstream analysis (A–C) such as beta diversity, alpha diversity, effect size estimation and differential abundance, and 5) Functional genes and pathways analysis using HUMAnN v3.0 and its UniRef 50, Pfam, and MetaCyc pathway databases. Figure S4. Difference in the composition of core microbiota between Orang Asli cohort and KL cohort in different taxonomic rank, which include: A Class, B Order, C Family, D Genus, and E Species. Figure S5. Difference in the composition of core microbiota between different geographical location in different taxonomic rank, which include A Family, B Genus, and C Species. Figure S6. A Bar plot of the top 20 species that best predict the difference of the core gut microbiota between Orang Asli (OA) cohort and Kuala Lumpur (KL) cohort using a Random Forest classification model B and C box plots displaying the selected core microbial species that have high variation between Orang Asli (OA) cohort and Kuala Lumpur (KL) cohort based on the Random Forest analysis. The relative abundances of core microbial species between Orang Asli cohort and KL cohort were tested using Wilcoxon rank sum test. B Species with significant higher abundance in Orang Asli cohort than KL cohort, which include (from left to right): HRGM Genome 3145, Gemmiger sp900539695, and [file 40168_2022_1385_MOESM2_ESM.zip › Supplementary_Figure_S5.pdf]

Fig. S6

A

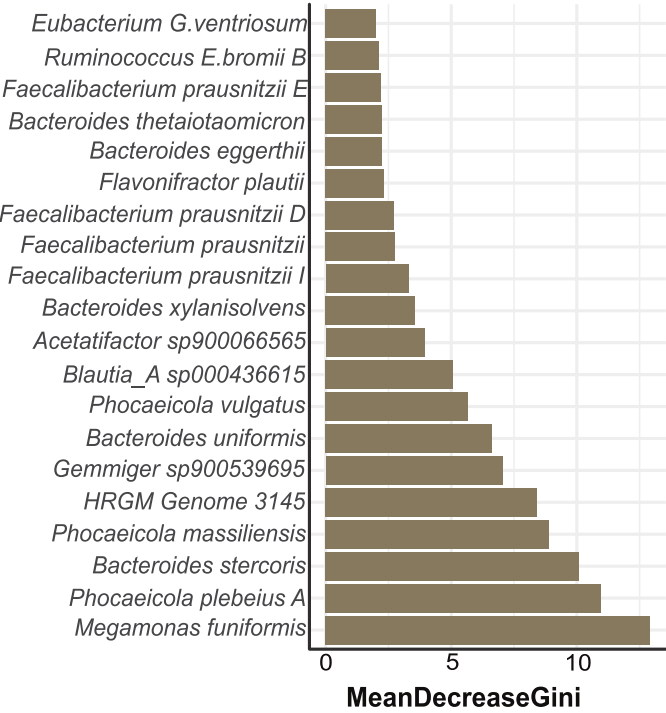

B

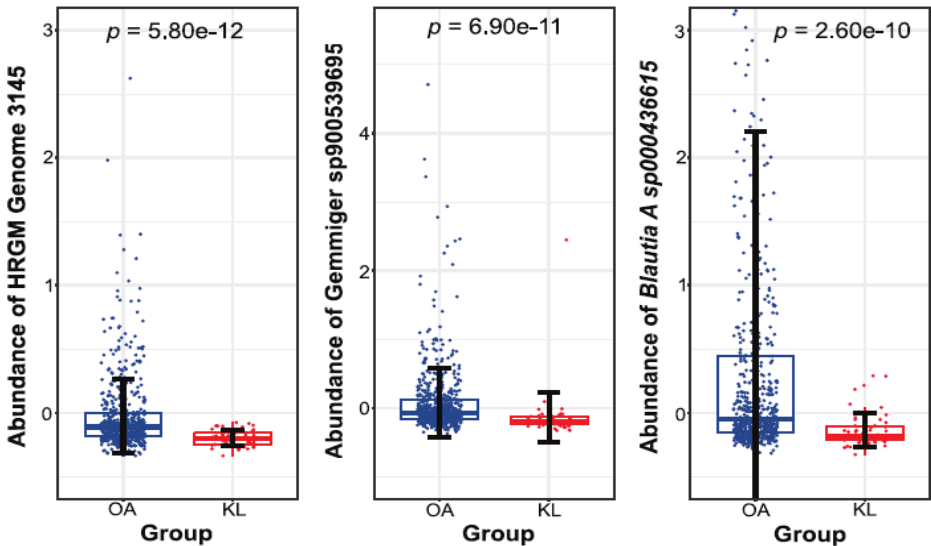

C

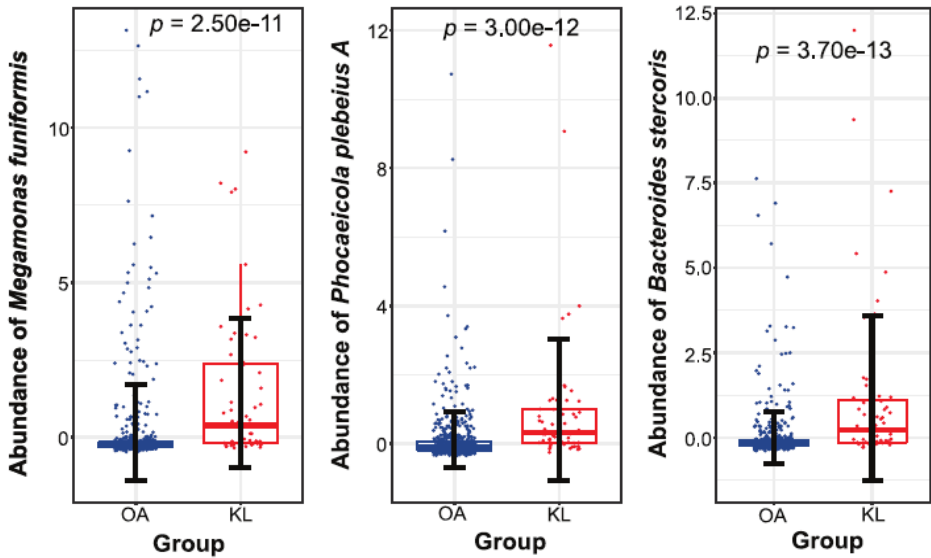

Supplement: Supplementary file 3 — Additional file 2: Figure S1. A geographic map showing the locations of each village and the Kuala Lumpur city in Peninsular Malaysia (stars and numbers) together with a table with other information including states, tribes and subtribes. Figure S2. A flow diagram of the total number of subjects (Orang Asli and urban citizens from Kuala Lumpur) involved in both the pre-anthelmintic and post-anthelmintic of this study. Figure S3. A flow diagram summarizing the bioinformatic analysis from raw reads, 1) Quality filtering, remove human reads and adapter (KneadData), taxonomic classification (Kraken2 and Bracken2), 3) K-mer based approach (Sourmash), 4) Estimation of bacterial growth rate (GRiD) to downstream analysis (A–C) such as beta diversity, alpha diversity, effect size estimation and differential abundance, and 5) Functional genes and pathways analysis using HUMAnN v3.0 and its UniRef 50, Pfam, and MetaCyc pathway databases. Figure S4. Difference in the composition of core microbiota between Orang Asli cohort and KL cohort in different taxonomic rank, which include: A Class, B Order, C Family, D Genus, and E Species. Figure S5. Difference in the composition of core microbiota between different geographical location in different taxonomic rank, which include A Family, B Genus, and C Species. Figure S6. A Bar plot of the top 20 species that best predict the difference of the core gut microbiota between Orang Asli (OA) cohort and Kuala Lumpur (KL) cohort using a Random Forest classification model B and C box plots displaying the selected core microbial species that have high variation between Orang Asli (OA) cohort and Kuala Lumpur (KL) cohort based on the Random Forest analysis. The relative abundances of core microbial species between Orang Asli cohort and KL cohort were tested using Wilcoxon rank sum test. B Species with significant higher abundance in Orang Asli cohort than KL cohort, which include (from left to right): HRGM Genome 3145, Gemmiger sp900539695, and [file 40168_2022_1385_MOESM2_ESM.zip › Supplementary_Figure_S6.pdf]

Fig. S7

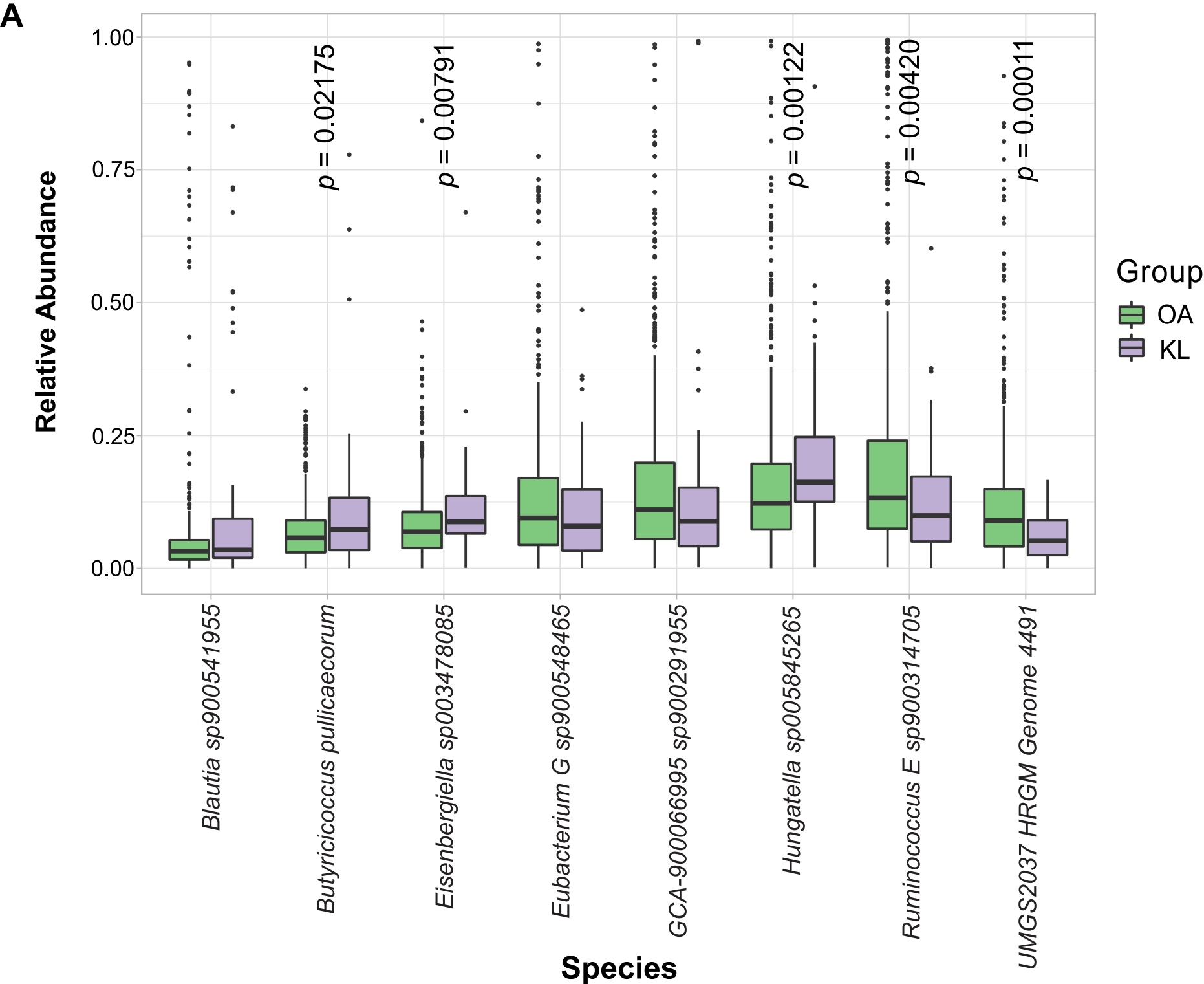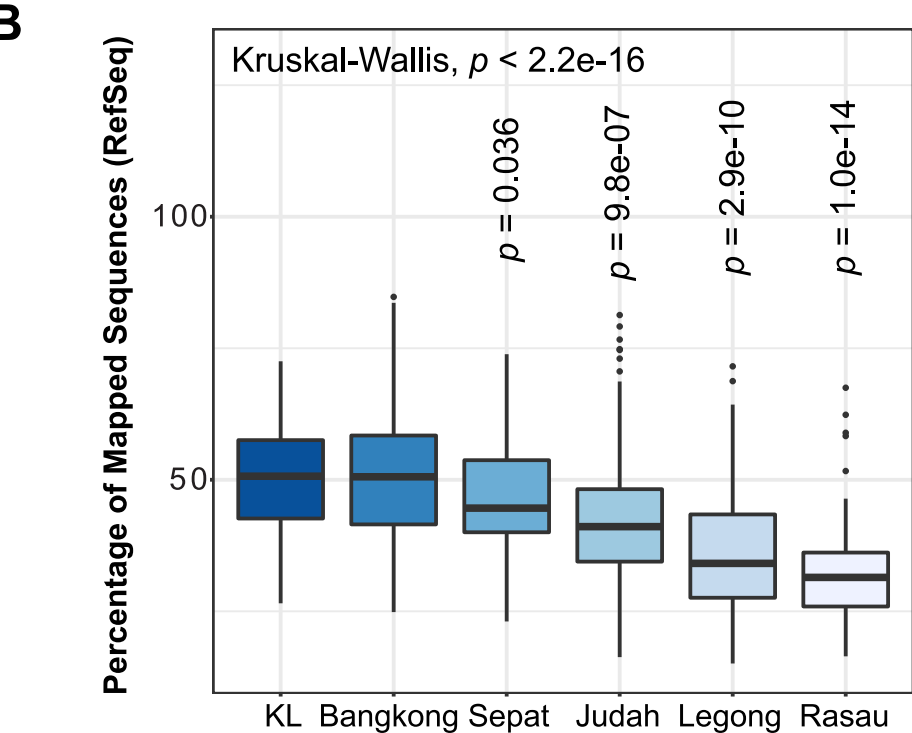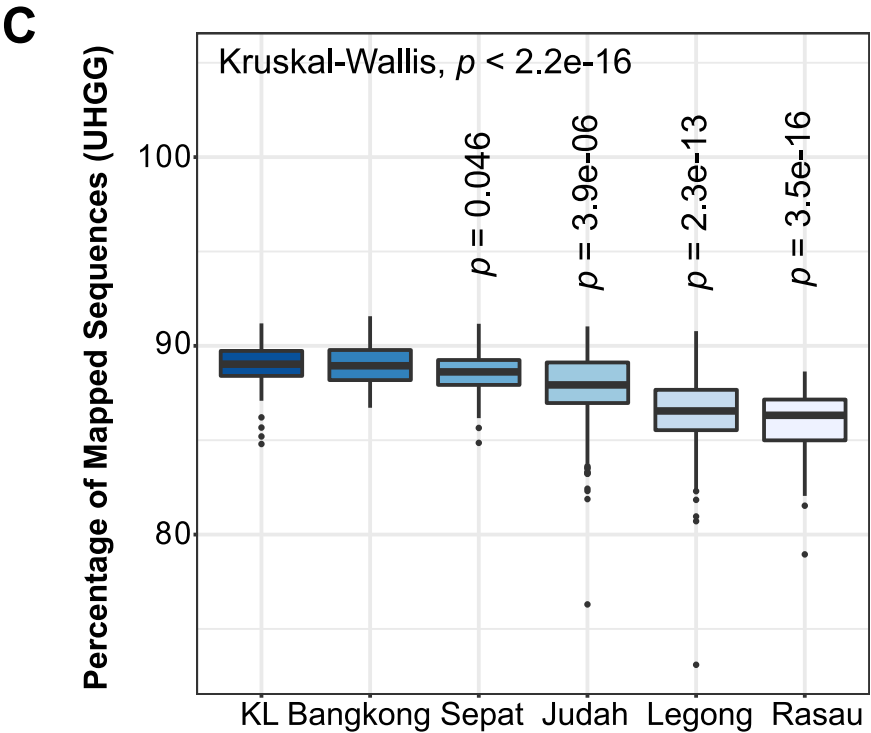

Supplement: Supplementary file 3 — Additional file 2: Figure S1. A geographic map showing the locations of each village and the Kuala Lumpur city in Peninsular Malaysia (stars and numbers) together with a table with other information including states, tribes and subtribes. Figure S2. A flow diagram of the total number of subjects (Orang Asli and urban citizens from Kuala Lumpur) involved in both the pre-anthelmintic and post-anthelmintic of this study. Figure S3. A flow diagram summarizing the bioinformatic analysis from raw reads, 1) Quality filtering, remove human reads and adapter (KneadData), taxonomic classification (Kraken2 and Bracken2), 3) K-mer based approach (Sourmash), 4) Estimation of bacterial growth rate (GRiD) to downstream analysis (A–C) such as beta diversity, alpha diversity, effect size estimation and differential abundance, and 5) Functional genes and pathways analysis using HUMAnN v3.0 and its UniRef 50, Pfam, and MetaCyc pathway databases. Figure S4. Difference in the composition of core microbiota between Orang Asli cohort and KL cohort in different taxonomic rank, which include: A Class, B Order, C Family, D Genus, and E Species. Figure S5. Difference in the composition of core microbiota between different geographical location in different taxonomic rank, which include A Family, B Genus, and C Species. Figure S6. A Bar plot of the top 20 species that best predict the difference of the core gut microbiota between Orang Asli (OA) cohort and Kuala Lumpur (KL) cohort using a Random Forest classification model B and C box plots displaying the selected core microbial species that have high variation between Orang Asli (OA) cohort and Kuala Lumpur (KL) cohort based on the Random Forest analysis. The relative abundances of core microbial species between Orang Asli cohort and KL cohort were tested using Wilcoxon rank sum test. B Species with significant higher abundance in Orang Asli cohort than KL cohort, which include (from left to right): HRGM Genome 3145, Gemmiger sp900539695, and [file 40168_2022_1385_MOESM2_ESM.zip › Supplementary_Figure_S7.pdf]

Fig. S8

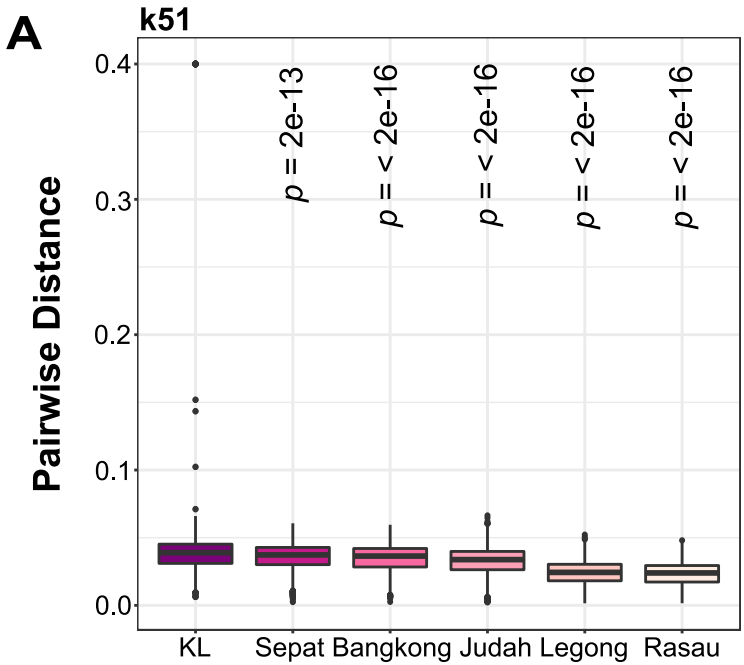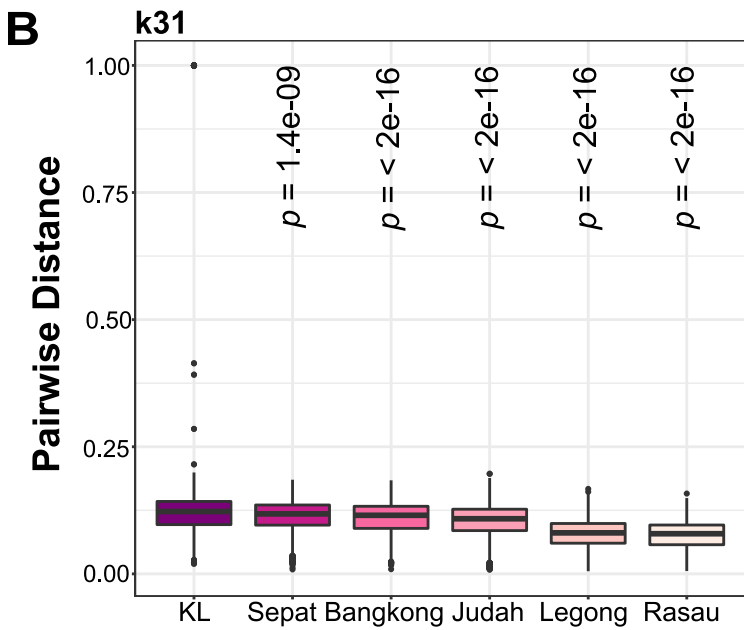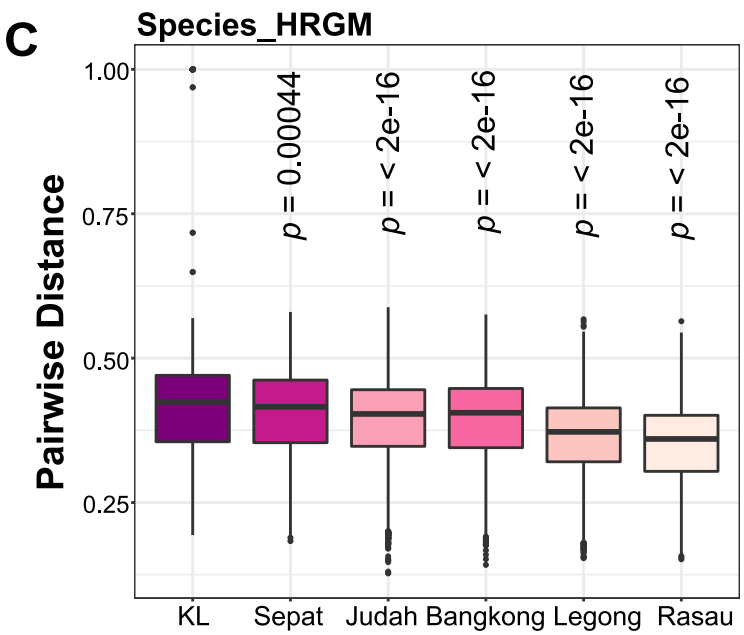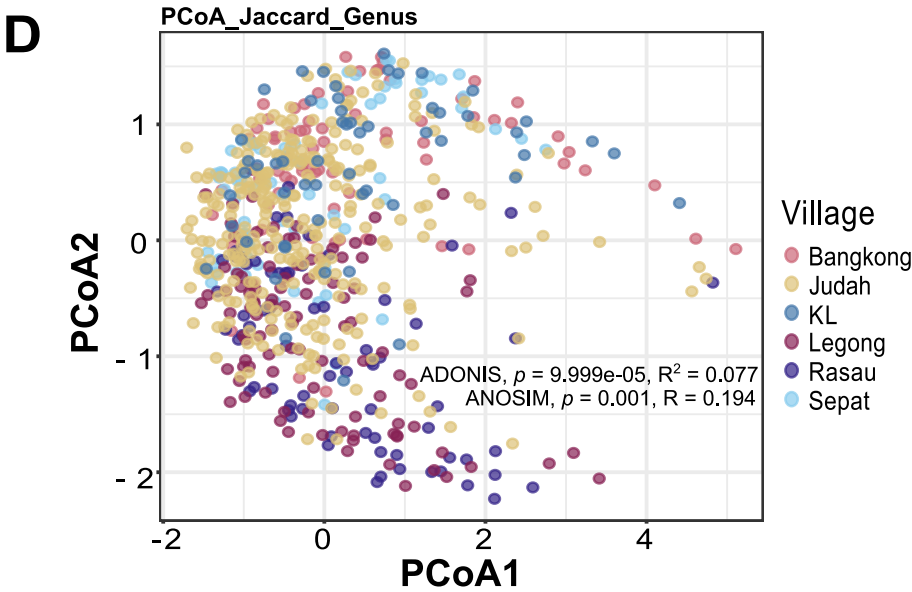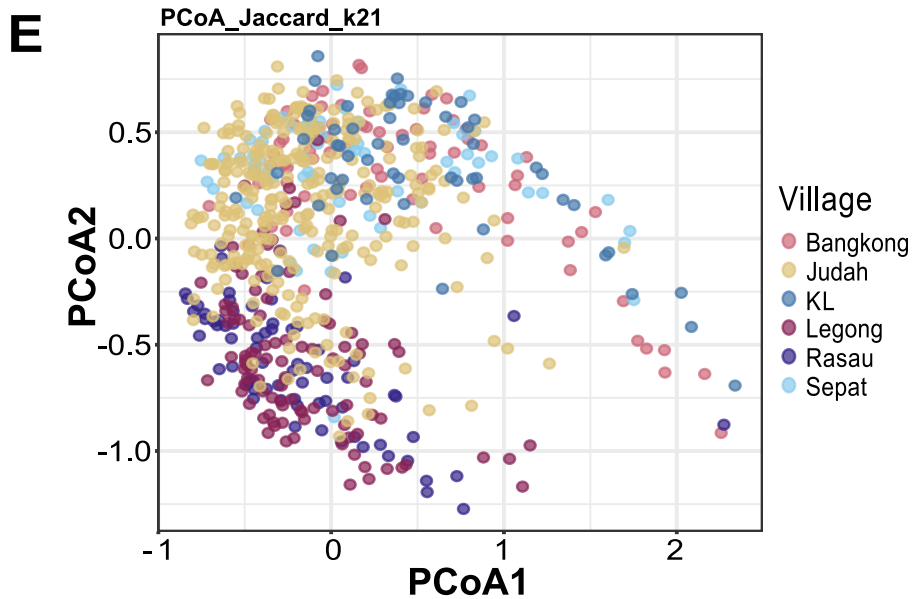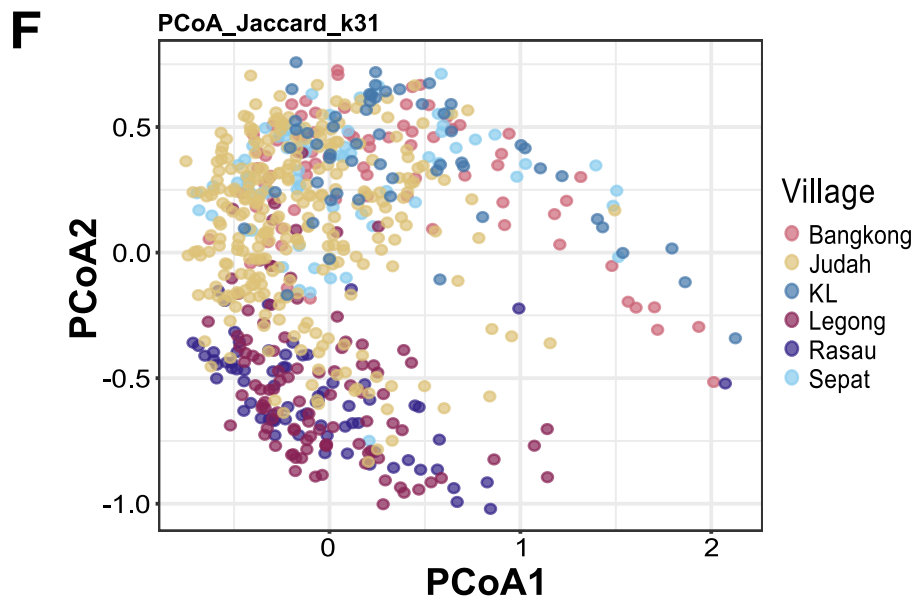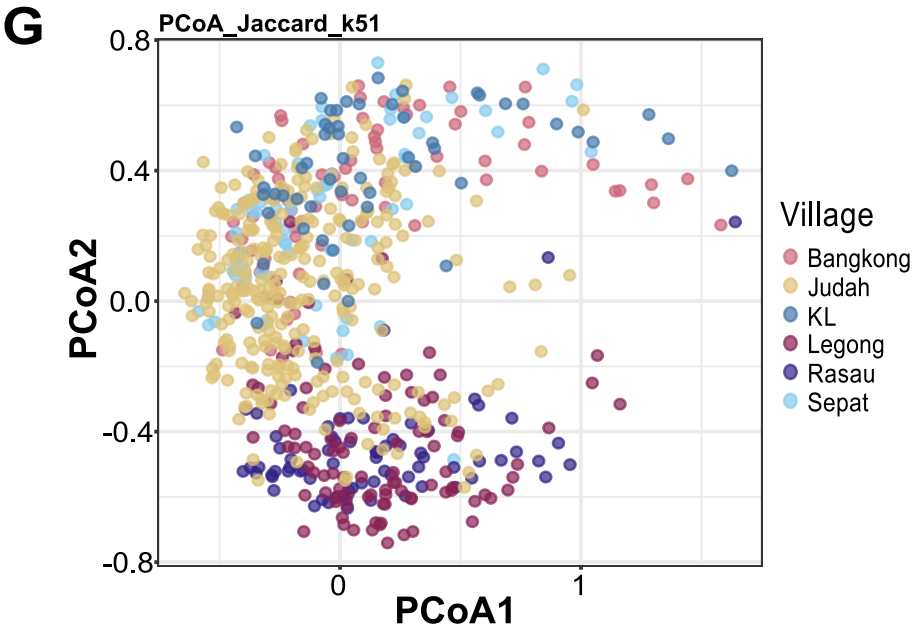

Supplement: Supplementary file 3 — Additional file 2: Figure S1. A geographic map showing the locations of each village and the Kuala Lumpur city in Peninsular Malaysia (stars and numbers) together with a table with other information including states, tribes and subtribes. Figure S2. A flow diagram of the total number of subjects (Orang Asli and urban citizens from Kuala Lumpur) involved in both the pre-anthelmintic and post-anthelmintic of this study. Figure S3. A flow diagram summarizing the bioinformatic analysis from raw reads, 1) Quality filtering, remove human reads and adapter (KneadData), taxonomic classification (Kraken2 and Bracken2), 3) K-mer based approach (Sourmash), 4) Estimation of bacterial growth rate (GRiD) to downstream analysis (A–C) such as beta diversity, alpha diversity, effect size estimation and differential abundance, and 5) Functional genes and pathways analysis using HUMAnN v3.0 and its UniRef 50, Pfam, and MetaCyc pathway databases. Figure S4. Difference in the composition of core microbiota between Orang Asli cohort and KL cohort in different taxonomic rank, which include: A Class, B Order, C Family, D Genus, and E Species. Figure S5. Difference in the composition of core microbiota between different geographical location in different taxonomic rank, which include A Family, B Genus, and C Species. Figure S6. A Bar plot of the top 20 species that best predict the difference of the core gut microbiota between Orang Asli (OA) cohort and Kuala Lumpur (KL) cohort using a Random Forest classification model B and C box plots displaying the selected core microbial species that have high variation between Orang Asli (OA) cohort and Kuala Lumpur (KL) cohort based on the Random Forest analysis. The relative abundances of core microbial species between Orang Asli cohort and KL cohort were tested using Wilcoxon rank sum test. B Species with significant higher abundance in Orang Asli cohort than KL cohort, which include (from left to right): HRGM Genome 3145, Gemmiger sp900539695, and [file 40168_2022_1385_MOESM2_ESM.zip › Supplementary_Figure_S8.pdf]

Fig. S9

A

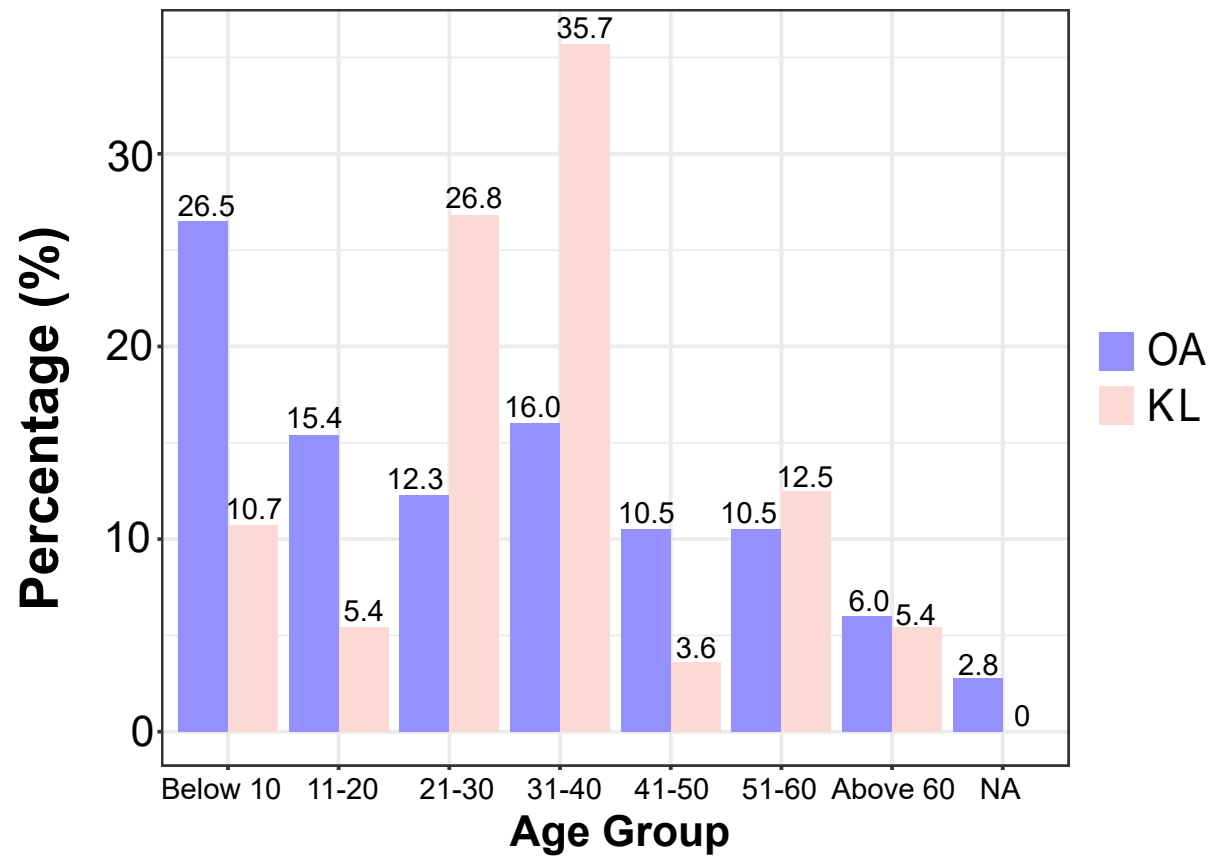

B

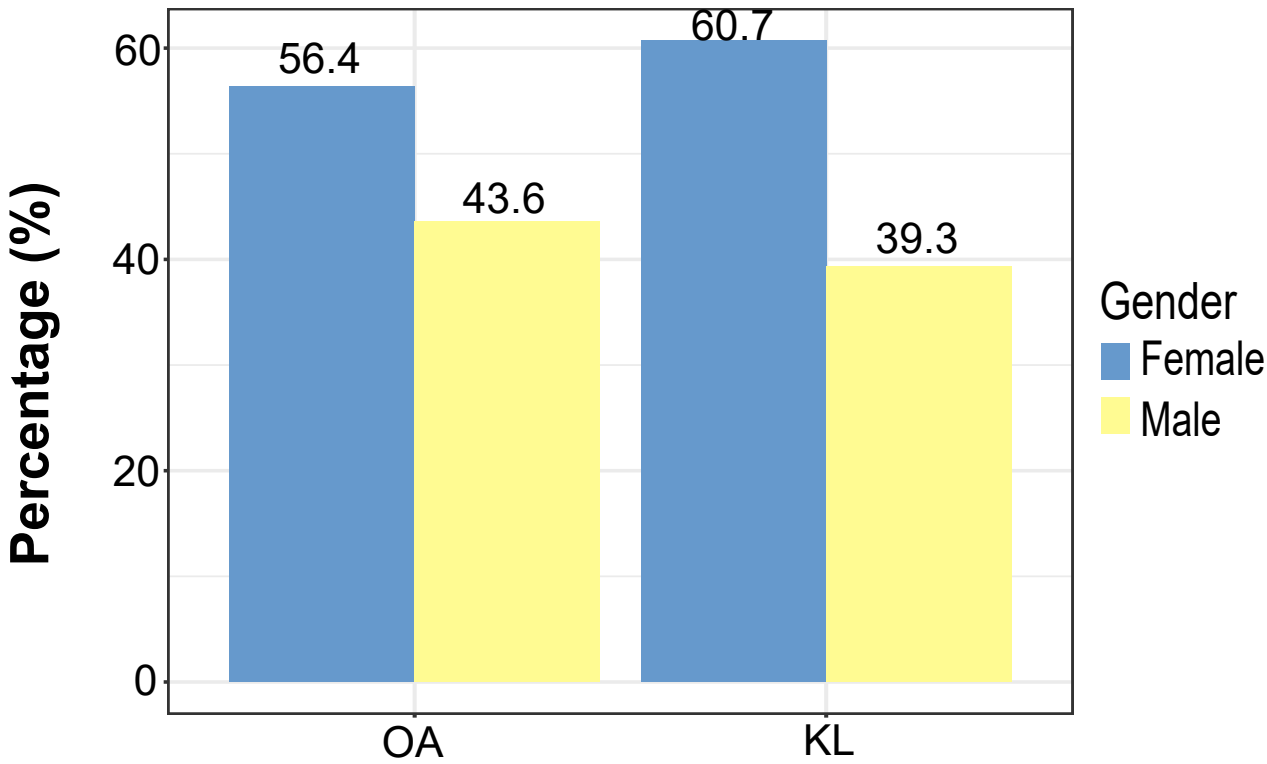

C

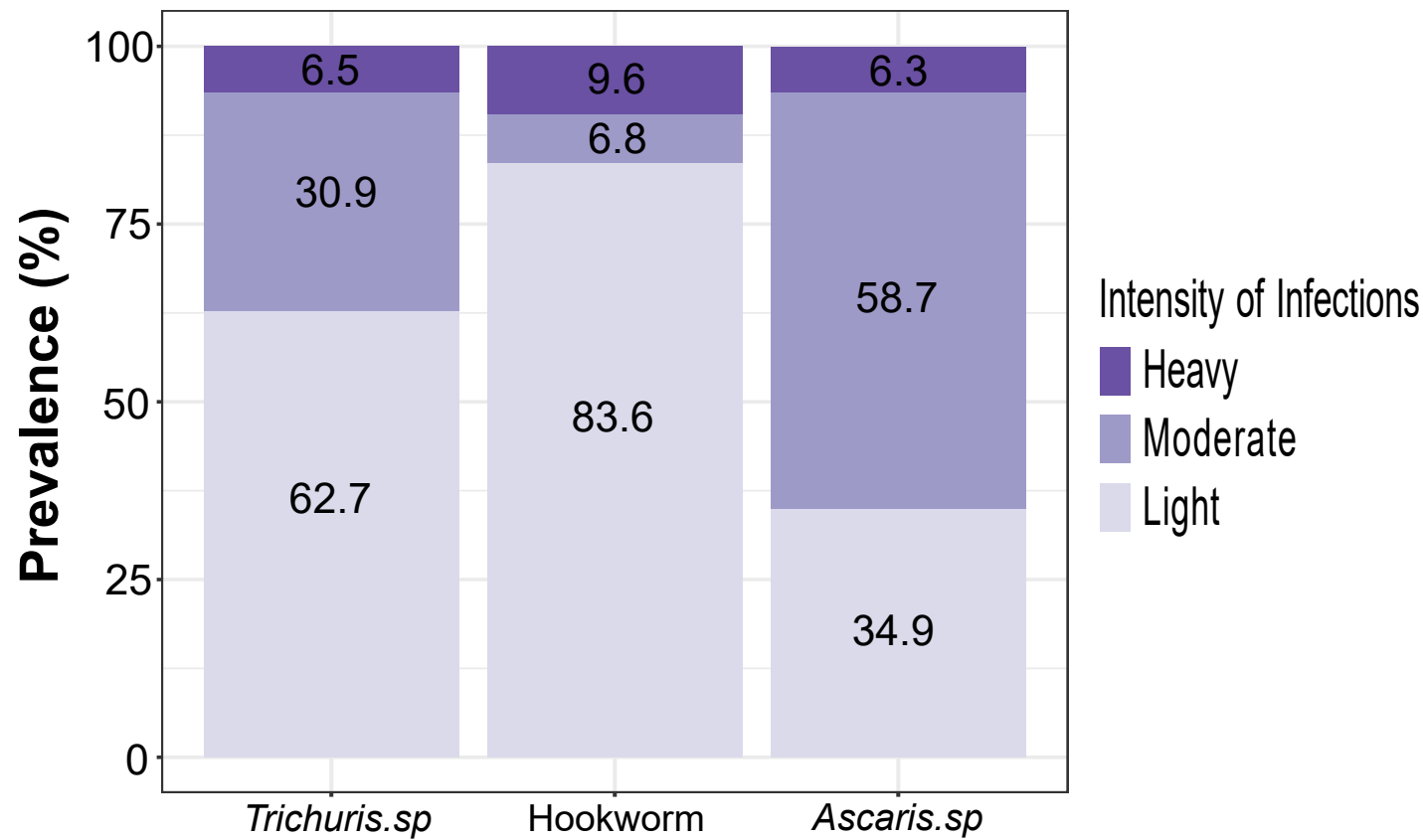

Supplement: Supplementary file 3 — Additional file 2: Figure S1. A geographic map showing the locations of each village and the Kuala Lumpur city in Peninsular Malaysia (stars and numbers) together with a table with other information including states, tribes and subtribes. Figure S2. A flow diagram of the total number of subjects (Orang Asli and urban citizens from Kuala Lumpur) involved in both the pre-anthelmintic and post-anthelmintic of this study. Figure S3. A flow diagram summarizing the bioinformatic analysis from raw reads, 1) Quality filtering, remove human reads and adapter (KneadData), taxonomic classification (Kraken2 and Bracken2), 3) K-mer based approach (Sourmash), 4) Estimation of bacterial growth rate (GRiD) to downstream analysis (A–C) such as beta diversity, alpha diversity, effect size estimation and differential abundance, and 5) Functional genes and pathways analysis using HUMAnN v3.0 and its UniRef 50, Pfam, and MetaCyc pathway databases. Figure S4. Difference in the composition of core microbiota between Orang Asli cohort and KL cohort in different taxonomic rank, which include: A Class, B Order, C Family, D Genus, and E Species. Figure S5. Difference in the composition of core microbiota between different geographical location in different taxonomic rank, which include A Family, B Genus, and C Species. Figure S6. A Bar plot of the top 20 species that best predict the difference of the core gut microbiota between Orang Asli (OA) cohort and Kuala Lumpur (KL) cohort using a Random Forest classification model B and C box plots displaying the selected core microbial species that have high variation between Orang Asli (OA) cohort and Kuala Lumpur (KL) cohort based on the Random Forest analysis. The relative abundances of core microbial species between Orang Asli cohort and KL cohort were tested using Wilcoxon rank sum test. B Species with significant higher abundance in Orang Asli cohort than KL cohort, which include (from left to right): HRGM Genome 3145, Gemmiger sp900539695, and [file 40168_2022_1385_MOESM2_ESM.zip › Supplementary_Figure_S9.pdf]

Fig. S24

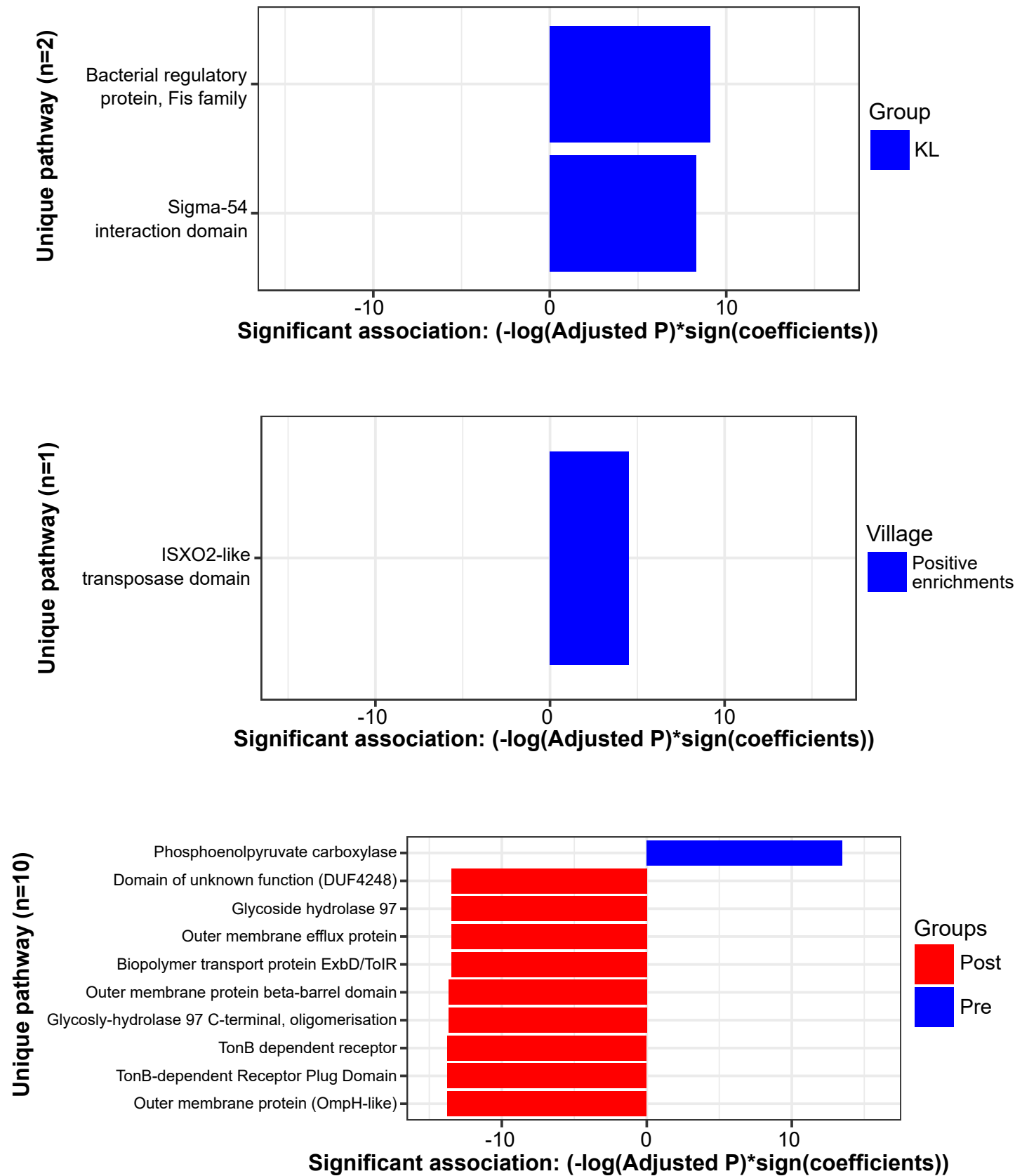

Supplement: Supplementary file 3 — Additional file 2: Figure S1. A geographic map showing the locations of each village and the Kuala Lumpur city in Peninsular Malaysia (stars and numbers) together with a table with other information including states, tribes and subtribes. Figure S2. A flow diagram of the total number of subjects (Orang Asli and urban citizens from Kuala Lumpur) involved in both the pre-anthelmintic and post-anthelmintic of this study. Figure S3. A flow diagram summarizing the bioinformatic analysis from raw reads, 1) Quality filtering, remove human reads and adapter (KneadData), taxonomic classification (Kraken2 and Bracken2), 3) K-mer based approach (Sourmash), 4) Estimation of bacterial growth rate (GRiD) to downstream analysis (A–C) such as beta diversity, alpha diversity, effect size estimation and differential abundance, and 5) Functional genes and pathways analysis using HUMAnN v3.0 and its UniRef 50, Pfam, and MetaCyc pathway databases. Figure S4. Difference in the composition of core microbiota between Orang Asli cohort and KL cohort in different taxonomic rank, which include: A Class, B Order, C Family, D Genus, and E Species. Figure S5. Difference in the composition of core microbiota between different geographical location in different taxonomic rank, which include A Family, B Genus, and C Species. Figure S6. A Bar plot of the top 20 species that best predict the difference of the core gut microbiota between Orang Asli (OA) cohort and Kuala Lumpur (KL) cohort using a Random Forest classification model B and C box plots displaying the selected core microbial species that have high variation between Orang Asli (OA) cohort and Kuala Lumpur (KL) cohort based on the Random Forest analysis. The relative abundances of core microbial species between Orang Asli cohort and KL cohort were tested using Wilcoxon rank sum test. B Species with significant higher abundance in Orang Asli cohort than KL cohort, which include (from left to right): HRGM Genome 3145, Gemmiger sp900539695, and [file 40168_2022_1385_MOESM2_ESM.zip › Supplementary_Figure_S24.pdf]

Fig. S23

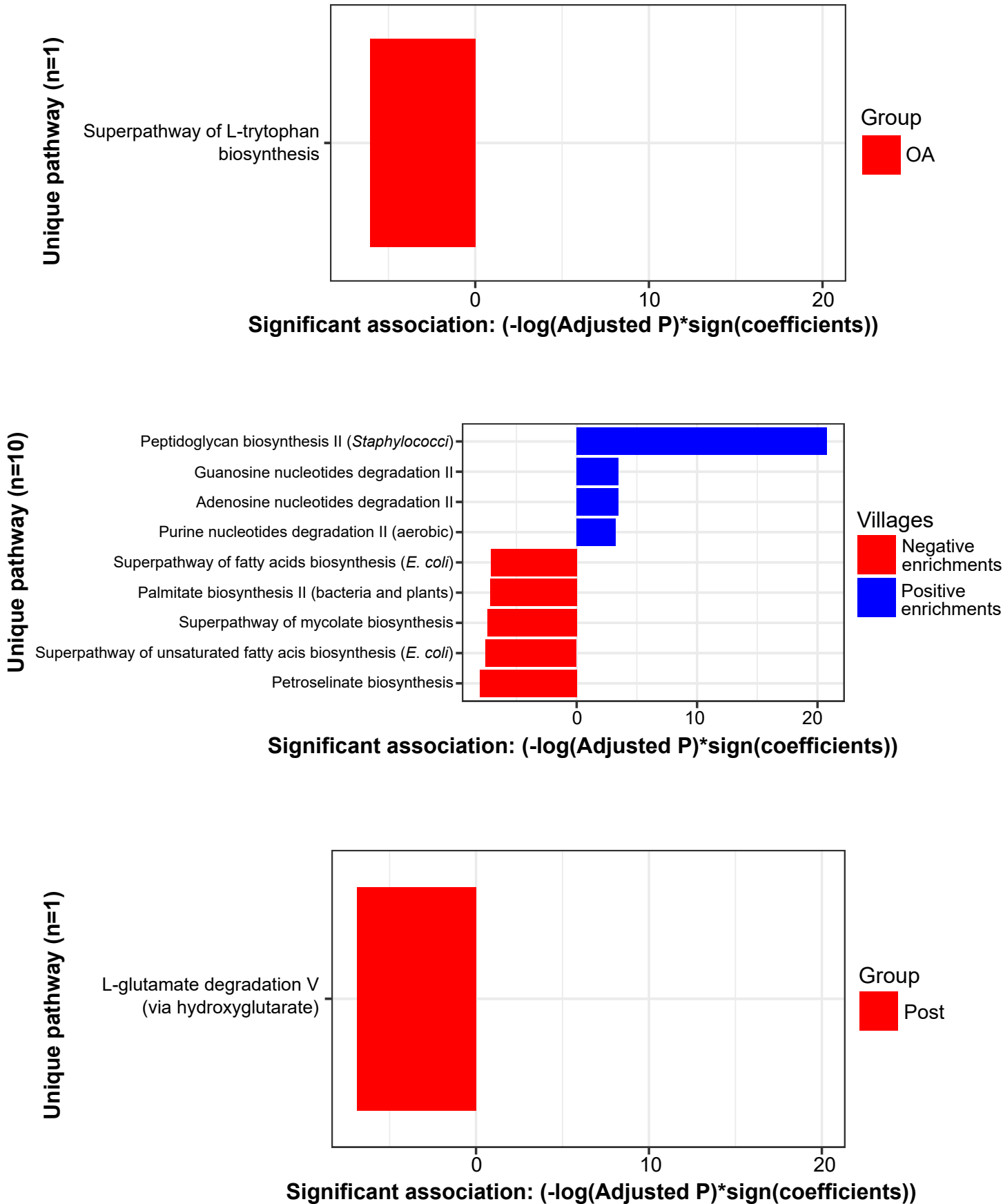

Supplement: Supplementary file 3 — Additional file 2: Figure S1. A geographic map showing the locations of each village and the Kuala Lumpur city in Peninsular Malaysia (stars and numbers) together with a table with other information including states, tribes and subtribes. Figure S2. A flow diagram of the total number of subjects (Orang Asli and urban citizens from Kuala Lumpur) involved in both the pre-anthelmintic and post-anthelmintic of this study. Figure S3. A flow diagram summarizing the bioinformatic analysis from raw reads, 1) Quality filtering, remove human reads and adapter (KneadData), taxonomic classification (Kraken2 and Bracken2), 3) K-mer based approach (Sourmash), 4) Estimation of bacterial growth rate (GRiD) to downstream analysis (A–C) such as beta diversity, alpha diversity, effect size estimation and differential abundance, and 5) Functional genes and pathways analysis using HUMAnN v3.0 and its UniRef 50, Pfam, and MetaCyc pathway databases. Figure S4. Difference in the composition of core microbiota between Orang Asli cohort and KL cohort in different taxonomic rank, which include: A Class, B Order, C Family, D Genus, and E Species. Figure S5. Difference in the composition of core microbiota between different geographical location in different taxonomic rank, which include A Family, B Genus, and C Species. Figure S6. A Bar plot of the top 20 species that best predict the difference of the core gut microbiota between Orang Asli (OA) cohort and Kuala Lumpur (KL) cohort using a Random Forest classification model B and C box plots displaying the selected core microbial species that have high variation between Orang Asli (OA) cohort and Kuala Lumpur (KL) cohort based on the Random Forest analysis. The relative abundances of core microbial species between Orang Asli cohort and KL cohort were tested using Wilcoxon rank sum test. B Species with significant higher abundance in Orang Asli cohort than KL cohort, which include (from left to right): HRGM Genome 3145, Gemmiger sp900539695, and [file 40168_2022_1385_MOESM2_ESM.zip › Supplementary_Figure_S23.pdf]
